# Supplementary material for: “Without a man’s decision, nothing works”: Building resilience to Rift Valley fever in pastoralist communities in Isiolo Kenya
Source: PLoS One. 2025 Jan 28;20(1):e0316015. doi: 10.1371/journal.pone.0316015 (PMC11774392; doi:10.1371/journal.pone.0316015)
Supplement: S1 Dataset — (ZIP) [file pone.0316015.s001.zip › Supporting Information Files/File 1.docx]

**E**: **The first thing I would like us to discuss is what kind of livestock you keep?**

**R7**: Cows, Goats

**E**: **Please raise your voice when you speak so that it can be heard clearly.**

**R7**: Cows, goats, donkey, and chicken

**R8**: Chicken

**E**: **Chicken**

*(Birds chirping)*

**E**: **Is there something else that she might have forgotten to mention?**

**R5**: There are camels too.

R3: sheep

**E**: **Among these animals, I would like you to tell me which of them belongs to men and which ones belong to women.**

**R2 & R8**: They both own all the livestock mentioned.

**E**: **R2 tell us what men own and what women own.**

**R2**: Women own goats.

**R7**: Donkeys also belong to women.

**R5**: Women also own goats and look after them. Men own cows and camels and they look after them.

**R1:** Men own big animals like cows and camel and look after the cows and camels while women own and look after small animals like goats and chicken

**E**: **R6 what do you think?**

**R6:** Men own cows, goats, and camel and they look after them.

**E**: **Do women own cattle?**

**R4**: What?

**E**: **Do women own cows?**

**R4**: Yes they have.

**R2**: They have even if it is one.

**R6**: Yes women have cows even if it is one which she was given as her dowry.

**R3**: She has one that was given to her as dowry.

**E**: **The first question I would like to ask is which type of diseases attack your livestock**

**R1**: The disease that kills them is Kala-azar and mosquitoes

*(Sound of a child calling his mum)*

**R2**: Ticks

*(Sound of motorbike passing)*

**R1**: The worst disease is Kala azar. It infects the animals. They urinate blood, they become sick and cannot walk. They just sit. Also, in humans. When it infects the children, they have bloated stomach, fever and they become weak.

**E**: **Okay, do we have any other disease?**

**R2**: Yes, there is, stomachache and fever.

**E**: **Please let us speak in turns so that the person who will be listening to these recording will be able to hear what we are saying. What were you saying R5?**

**R5**: I said people suffer from stomachaches, like typhoid.

**E**: **What about animals?**

*(Sound of someone coughing)*

**R5**: Even in Animals, they have fever

**E:** **R1, what do you think?**

**R1**: When animals go to graze, they are bitten by Mosquitoes and tsetse fly

*(Motorcycle passing)*

**R4**: Yes, also Kala Azar

**E**: **R6**: Like she said kala Azar, Mosquitoes

**E**: **Other than Kala azar and mosquitoes are there any other diseases that affect livestock?**

**R6**: foot and mouth, CCP (Contagious Caprine Pleuropnemonia)

**E**: **Foot and Mouth, CCP. And have you had of RVF?**

R6: yes, it also affects the animals

R2: The fever is brought by mosquitoes

R3: Fever is brought by Mosquitoes

| ID | RVF Disease signs livestock | ID | RVF Disease signs in humans |
| --- | --- | --- | --- |
| R3 | Diarrhea | R5 | Fever/shivering |
| R6 | Abortion, bloody urine | R3 | Swollen stomach |
| R4 | Death | R8 | Vomiting |
| R7 | Diarrhea | R1 & R3 | Yellow eyes |
|  | Yellow meat | R8 | headache |
| R10 | Fever | R4 | Bloated stomach |
| R2 | Diarrhea |  |  |

**E: What happens to humans or livestock when they get infected by RVF?**

R1: The goat dies

*(Sounds of birds chirping)*

**E; the Goats die**

R2 :Yes they get fever, they are tied to a tree, they are vaccinated, and some survive some die

**E: Other than death how else does it affect livestock**

*(Sounds of birds chirping)*

R3: They diarrhea

**E: They diarrhea?**

R5: They Shiver

*(Birds chirping)*

**E: Please tap her**

R8: Halim

R10: Yes

**(Birds chirping)**

**E: Please Move closer please tell us how livestock behave when they are affected by RVF**

R10: Pardon

**E: Tell us the behavior of an animal which has been infected by RVF**

R10: It shivers falls down and die

R6: how do you know?

**E: how else can you tell?**

R2: they diarrhea

R6: Yes, they diarrhea

R7: massive abortion for sheep and goats

**E: Why didn’t you say that?**

**E: How does RVF affect human OR what are the signs and symptoms of RVF in humans**

*(Sound of a child playing)*

*Women talking in chorus cannot be heard clearly*

*(Sound of a child calling out someone)*

R7: Humans experience fever, they vomit and diarrhea

R5: They ran a fever

R3: They have bloated stomach

R4: They vomit and diarrhea

R3: they vomit is yellow in color

R1: the eyes turn green, yellow

R2: Also, in animals the meet turn color

**E: Are we still talking about RVF?**

R1 & R2: yes, it is RVF these are the symptoms brought by the mosquitoes

R6: I’m saying cows start shivering and fall and die

R8: RVF has caused a lot of deaths to our livestock they urinate blood

*(Voice of a child playing and women discussing in low tones)*

*(Birds chirping)*

**Second question**

**E: We are going to discuss our second question now I would like you to tell me how humans and livestock get RVF infection**

R8: animals get infected after they are bitten by the mosquito

R1: when it rains is when the mosquitoes and the tsetse fly infect the area

R2: sometimes it comes with the seasonal river. They use the water as their breeding place they bite both humans and livestock

**E: Is there something else R5**

R5: yes, when are bitten by mosquitoes they run a fever

**E: Ehhh!**

R5: When it rains there are a lot of mosquitoes, and they bite you

R3: tsetse fly is also in plenty in the area, so they cause RVF

*(Women discussing)*

**E: We are talking about RVF, and I would like you to tell me how humans and livestock get infected with RVF**

R5, 7&3: when they are bitten by mosquitoes

**E: R8 Can humans get infected by RVF from animals?**

R8: yes, you can get it from animals

R1: you can get it from animals when you eat their meat and drink their milk

R6: when you drink tea made with milk from an infected animal

R7: If that animal is sick and starts to bleed from the mouth the urine also has blood

R8: if you eat meat from that animal you are going to be infected

**E: is there any other way the infection is passed?**

R1; I don’t know

**E:**

**E: How can you tell if a person has been infected with RVF?**

R7: when the mosquitoes start breeding is when the infections also start, we take people to the hospital

R8: when you go to the hospital the doctor examines you and diagnosis the infection

R3: In humans it is the doctor that tells you

**E: Let’s listen to one person at a time**

R6: They come to inspect the animals, vaccinate them, give them oral medicine and we are advised not to drink milk from them for a period we are also advised not to eat their meat

**E: R2 what’s your opinion**

R2: we are told not to eat or drink milk from infected animals, or we will get infected

**E: Is there any other way you learn about RVF**

Chorus – no

**E: when you look after the sick animals who take care of other household chores that you normally do?**

R7: if the children are at home, they do the other chores if they are at the location of the sick animal they will take care of them in short whoever is present will help the animals

**E: are there any other changes in the care?**

R3: we move the animals from the infection prone area, vaccinate them and spray them

R6: during the dry season, we have dig well for them

**E: you dig well for them?**

Chorus – yes during the dry season

**E: even in the presence of the disease**

Chorus – yes

**E: who digs the well?**

R7: the men are the ones who dig the well or you pay people to do it for you. Men are strong and energetic

*(Motorbike passing)*

**E: Please repeat, I couldn’t hear you clearly because of the noise from the motorbike.**

R8: During the dry season, the animals are moved to places where they can get food.

**E: Who moves with them?**

R3: My children and I. I cannot construct a shelter, so my children do it for me.

**E: Our fifth question is how is this disease treated? When you answer please raise your voice**.

R1: They are taken to the hospital.

R3: yes, the patient has to see a doctor. Unlike animals, human beings cannot be slaughtered. We just take them to the hospital. If there isn’t any medicine at the facility, they are prescribed for you. If they are not able to treat you, they refer you to Garbatulla or Habaswein.

**E: Is there any other way they can get assistance?**

R5: There is nothing else we do. We don’t know about medicine.

**E: Do you go to public of private hospital?**

R4: We go to the hospital in our area. We don’t pay for services.

**E: Are there people who use traditional medicine?**

R2: Yes, there are people who use herbal medicine.

R3: The doctors here warned us not to bring people who have been treated by herbalist to them in case the herbals backfire.

**E: So the doctors warned you?**

R5: Yes, they did.

R7: If they take herbal medicine and get cured, they don’t need to seek medical attention. If they take the herbal medicine and their situation deteriorates, you take them to the doctor. The doctor asks you questions, and they don’t like it when he hears that he was treated by an herbalists.

**E: Do you use herbal medicine?**

R3: Nobody uses herbal medicine these days.

**E: Other than going to the hospital, is there any other way you can prevent yourself from getting infection.**

R5: When the mosquitoes start breeding, we use mosquito nets.

**E: Is there any other way other than mosquito nets?**

R3: Veterinarians visits us and spray the area with pesticides.

**E: Please let’s give each other chance to speak.**

R4: By burning the bushes to prevent the mosquito from breeding.

**E: Is there any other way?**

R8: That’s all.

*(Motorbike passing)*

**E: You earlier mentioned that you can get infections from animals’, right?**

All respondents: Yes

**E: What measures do you put in place to avoid getting infection from animals?**

R1: When an animal is diagnosed with the infection, we don’t drink their milk or eat the meat.

R3: The milk can be boiled and used later but we cannot eat the meat.

**E: What else do you do?**

R8: The animals are moved away from the homestead to a far place with the herder or any household member

R4: we move to the areas which are not infected to avoid losing all the animals.

**E: You earlier mentioned that that when these animals get infected, they end up having miscarriages right?** **How do you dispose off the fetus, and other organs?**

R3: We use our bare hands to dispose it.

**E: What are the risks of using bare hands?**

R7: You cannot get infected after disposing it. You wash your hands well.

R2: We also assist the animals to give birth, with our bare hands

R6: No, we don’t.

R4: You cannot get infected. You wash your hands well with soap and you are good to go.

**E: Do you drink milk from the animal that has been vaccinated?**

R2: Not on the first day of vaccination. We drink milk after a day or two.

R3: The doctors who vaccinate the animal also advises us not to eat meat from a vaccinated animal until one week is over.

**E: You have told me on how you prevent yourselves from getting infections from the animals. You have mentioned boiling milk, moving your animals, vaccination and not eating the meat. From the above measures, which one tops your list as the best prevention method?**

R2: Vaccination.

**E: Why do you say that?**

R2: Vaccination prevents the spread of the diseases. It prevents the animal from the deadly infection.

**E: Vaccination is the topmost. What follows it?**

R3: Moving animals to other areas where there are no infection.

*(Women discussing)*

*(Motorbike passing)*

*(A child crying)*

*Women speaking in low tones.*

**E: I would like now to tell you a story. We will discuss some questions later. Please listen carefully. There is a man whose name is Boru. He is married to Amina. They are pastoralists. They keep cows, camels, goats and sheep. In 2023, there was a disease outbreak in their area. Are we together? These disease affects both humans and livestock**.

**E: The first question I would like you to answer is does Amina have control over the animals in case they get infected?**

**Scores**

Amina-5

children-

Other family members-2

**Reasons for Amina**

**E: I am asking if Amina has control over the animals to the extent that she can sell them if she wants to.**

R2: Yes, she has. She can separate the animals. The ones that are sick should be separated from the healthy ones.

R3: She must consult from other family members because she may need help.

R1: she is the household head, and she makes the decision regarding to her household

R6: If she widowed, separated, then all the household decisions are made by the woman especially if the children are still young

R3: You don’t need to consult anyone to make a decision on what you own. You are the one who knows what to go through. You don’t have to consult your children especially if they are younger.

R5: She can and she cannot, at the end of the day, she is the one who takes care of the family. She plans for them so she doesn’t need to consult anyone.

Reasons for other family members

R7: If a woman is married, it doesn’t matter what she owns, she doesn’t have control. The husband is in control. And if the husband is not there and the children are young, the family members make decisions regarding to the resource in the household.

R8: It is important to consult people before making decisions. If you have children, it is even better. You discuss and make better choices. Same applies to when you consult your husband, He will advise you better and you make a good choice.

**E: What if Amina becomes sick, does she have to consult anyone before going to the hospital? Please show me using the cards what you think. Please tell me why you chose the card that you have in hand.**

**Scores**

Amina-0

children-6

Other family members-2

**Reasons for children**

R1: I said I would consult my children because they say that one hand doesn’t give a bath unless you use two hands.

*(Women discussing in low tones)*

*(Motorbike passing)*

R1: It is important to consult and give hope to each other and that is how we help each other.

R3: I’m used to consulting my children.

R5: It is important to consult your children because something might happen to you while you are there. Also, after the treatment you can tell them what you need, and they are able to help.

R7: The reason why I involve my children is that we are doing life together. Before I make any decision, I must consult them. I might go to the hospital and die and whatever I have belongs to them. It is important for us to know each other’s movement.

R2: I have to consult my children.

R2: My mistake, I’m sorry. I always consult my children.

**Reasons for other family members**

R2: When I leave my home to go the hospital, I will live my child with the person living with me. My child is young and needs to be cared for. I have to consult the person living with me so that she can take good care of her. On case anything happens, I have left my child and livestock with the guardian.

R4: The reason why I have to consult the person living with me is because I might need to be cared for in case anything happens to me while I’m at the hospital.

**E: My other question is, Can Amina sell her livestock and start a business? By show of cards please tell me if she can do it alone, consult her children or consult the people that live with her**.

**Scores**

Amina-0

children-7

Other family members-1

**Reasons for children**

*(Women discussing)*

R3: You need to consult your children. When you sell of part of your properties, you need to call them, and you decide together on how the money is going to be used. We discuss and come into an understanding. If they convince me that I am making a bad choice, I leave it. All of them. We sit together.

R: 2 She doesn’t have control. She has to sit with the children and agree together to avoid conflicts

R7: She is alone, and she cannot work alone. If she can’t work alone, then she cannot make her decisions alone. If that is what you mean, I’ll give you an example. I might have an idea in my head. Then I decide to call my children who are grown up and tell them about my idea. I sell it to them. We discuss and sometimes children have better ideas, and we agree to change something and in return we come up with a brilliant plan.

R5: If you make your decision without consulting them, they become very angry and they can even stop assisting you.

**E: I have another short story. There is a woman called Shariffa. She is 50years old. She is a widow. Let’s continue with our story. Shariffa is 50 years old and she owns livestock. She owns camel, cows, goats and sheep. She acquired the livestock together with her husband when he was alive. Have you all understood that?**

**E: Since her husband passed own, she lives with her children at her in-laws. There was an outbreak of a disease in their area. The disease is said to infect both humans and livestock. Shariffa was called to attend a training which related to the disease. They were going to be trained about how to protect themselves and also teach her people about the disease. Since she lives with her in-laws and her children, does she have the power to go and attend the meeting without consulting her children and in-laws? Or who should she consult? Please show me using the cards.**

**E: Why did you say that she should consult her children?**

**Scores**

Sharifah-1

children-7

Other family members-0

**Reasons for children**

R5: She should consult her children because they will be left in charge of the livestock. She cannot just wake up and leave without saying anything to her children.

R4: I have the same opinion.

R3: Before I do anything or go anywhere, I need to consult my child.

R6: You need to consult with your children. They are your immediate family.

Reasons for sharifah

R8: the mother is the household head and she has the responsibility to take care of her family

**E: How do you prevent yourself from getting infected by RVF as women?**

R1: We use mosquito nets.

R4: We move to a different place if our area is affected.

R3: If we are affected, we go to the hospital.

**E: Is there any other way that you prevent yourself from being infected?**

Chores: No.

**E: How do you get information about RVF?**

R4: When infected you buy yourself medicine.

**E: Are women allowed to attend the seminars or it is for men only**?

R8: Both men and women attend seminars.

**E: How did you learn about the RVF?**

R3: Doctors tell us about it.

**E: Human or veterinary doctors?**

R1: Veterinary doctors

R7: The veterinary doctors tell us about the disease.

**E: The veterinarians give you the information?**

R7: Yes, they do tell us about it.

R6: They used to come before the rainy season starts. They give us all the information that we need to know.

R3: There is a certain pesticide that is sprayed in our compounds which kills the mosquitoes.

**E: They spray the compound?**

R3: Yes, they spray the whole village.

**E: Are there other ways of getting information about the disease?**

R5: No

**E: Do you have radio?**

R6, R7& R2: We don’t own radios.

**E: You don’t listen to radio or there is no frequency here?**

R3: We don’t understand the language even if we owned one but we don’t have radios.

**E: Don’t you have relatives who call you and tell you about the infection and sometimes warn you?**

R6: Whatever we want to know, we are being told by the veterinary doctors who comes to visit.

**E: So you get information only from the veterinary doctors?**

R5: Also the human doctors.

R7: When you fall ill frequently, they advise you to stop taking milk for a while.

**E: What are some of the challenges you face while protecting yourselves from RVF infections.**

R1: In animals or in humans

**E: In both.**

R1: There are times when the market value for livestock is high. On those days, you can sell part of it, get money and by yourself Pesticide and Spraying can and you spray around your compound.

**E: That is financial constraint.**

R1: Yes.

**E: What else?**

*(women discussing)*

R6: Some people light firewood and the smoke which comes from it sends away the mosquitoes.

**E: How about people who lack knowledge about the disease?**

R5: Those who know about it are better, they know how to protect themselves. Those who lack the information might even lose their lives from it.

**E: What about the age?**

R2: Old people don’t really know about what’s happening same applies to young children. Most of the time they are the ones who get infected the most.

*(Women discussing)*

**E: You had mentioned earlier that it is the doctors and veterinarian that give you the information right. My question is what do they tell you?**

*(A woman calling someone)*

R3: When the veterinarians come around to check on the animals, if he finds the sick ones he vaccinates them and advises us on what to do. For example, not to drinks its milk for a period of time.

**E: What about the human’s doctor?**

R5: He advises us too on the safety of drinking boiled water and boiled milk.

**E: When the veterinarian doctor identifies the presence of RVF in the animals around the community and he comes to the community and tells them the news about the disease, what are some of the challenges you face as a community in trying to protect yourselves from getting infected.**

R2: The animal might have been already infected and the mosquito has already bitten you. You all become sick.
