# Supplementary material for: “Without a man’s decision, nothing works”: Building resilience to Rift Valley fever in pastoralist communities in Isiolo Kenya
Source: PLoS One. 2025 Jan 28;20(1):e0316015. doi: 10.1371/journal.pone.0316015 (PMC11774392; doi:10.1371/journal.pone.0316015)
Supplement: S1 Dataset — (ZIP) [file pone.0316015.s001.zip › Supporting Information Files/File 10.docx]

Enumerator: what type of livestock do you have?

Respondent: I say my name and mention it?

Enumerator: R2.

Respondent 2: we have cows, goats, sheep, and some people have camels.

Enumerator: anything she hasn’t mentioned? R6

Respondent: 6 We have hens.

Enumerator: anything that hasn’t been mentioned by the two? R1

Respondent 1: there is nothing.

Respondent: 3 Donkey.

Enumerator: donkey. Another thing is, do women own livestock?

Respondent: 3 yes.

Enumerator: R2 have said they have. R3 Do women have livestock?

Respondent 3: they have?

Enumerator: which ones?

Respondent 3: they have goats.

Enumerator: goats. Do they have another livestock? R8.

Respondent: 8 we have hen. Donkeys.

Enumerator: Donkeys. How do they own the livestock?

Respondent: 7 They get as dowry.

Enumerator: how else do they own without getting as dowry?

Respondent: 1 they are given by their parents.

Enumerator: given by parents, R1.

Men own which livestock?

Respondent: 2 cows.

Enumerator: cows and what?

Respondent: 3 they also have goats.

Enumerator: anything else?

Respondent: 4 They also have donkeys. Also, hens.

Enumerator: have said, men.

Respondent: 5 ooh you were saying men?

Enumerator: yes.

Respondent: 6 They have camels.

Enumerator: cows and camels can be recognized with who in the past?

Respondent: All Men.

Men used to herd it.

Enumerator: I mean who owns it?

Respondent: 2 Camels used to be known with men.

Enumerator: which disease commonly affects human beings and livestock in your community? You say your name and mention the disease.

Respondent: 8 According to me, there is *frosti* mainly affects us.

Respondent: 7 It affects goats and personally am affected since I cannot walk properly.

Enumerator: R6. Any other disease?

Respondent: there is Contagious Caprine Pleuropneumonia in livestock. Human beings are mostly cancerous.

Enumerator: R5 is there another disease?

Respondent 5: there is kala-azar.

Respondent 4: fever. Livestock and human beings become weak.

Enumerator: can you explain what type of fever?

Respondent 4: there is yellow fever.

Enumerator: yellow fever, anything else? R3.

Respondent 3: Rift Valley Fever.

Enumerator: Rift Valley Fever.

Respondent: 6 Another disease that human being’s contract from livestock is brucellosis.

Enumerator: brucellosis. Anything else?

Respondent: 7 During the rainy period there is anthrax, rabbis and there is Rift Valley Fever.

Enumerator: How do you call Rift Valley Fever in the local dialect?

Respondent: 5 qando *bini.*

Enumerator: is it *qando bini or qando bifti?*

Respondent:3 They are different. Yellow fever is *qando bifte* and Rift Valley Fever is *qando bini.*

Enumerator: now I will understand that during our discussion. Any other disease?

Respondent: 2 There is diarrhea in livestock and humans

Enumerator: now our second question is, what are the signs and symptoms of RVF?

Respondent: 1 they urinate blood.

Respondent: *all livestock* shiver

Enumerator: what are the signs for Rift Valley Fever? *Give me one pen please.*

Respondent: 3 they shiver.

Enumerator: shivering.

Respondent:6 they don’t eat grass.

Enumerator: they lack appetite. What is the other sign when they have Rift Valley Fever?

Respondent: *inaudible response.*

Enumerator: R5 what are the other signs when they have Rift Valley Fever?

Respondent 5: they have diarrhea.

Enumerator: how does diarrhea look?

Respondent 5: they are green in color.

Enumerator: R1.

Respondent 1: they are watery.

Enumerator: anything else? R2.

Respondent 2: we have said there is a fever.

Enumerator: is there another sign on livestock that has Rift Valley Fever?

Respondent: 4 They limp.

Enumerator: limping, anything else?

Respondent: 5 It has yellow meat when the livestock is slaughtered.

Enumerator: any other sign that you can know with?

Respondent: Massive death

Respondent: those who consume the meat from the affected livestock may also die.

Enumerator: is there another one?

Respondent: 4 some even act as if they have *Coenurosis.*

Respondent: 7 stillbirths in livestock

Respondent 2: when they have fever and they get stillbirth.

Enumerator: Any other sign?

What are the signs when the person is affected with Rift Valley Fever?

Respondent:3 they have fever.

Enumerator: remember to say your name.

Respondent:6 it causes bloating of the stomach.

Enumerator: any other sign on human beings?

Respondent: 7 yellow vomit.

Respondent: 8 losses of appetite.

Enumerator: any other sign?

Respondent: 1 some might even die.

*Coughing.*

The major signs of RVF in livestock are mass stillbirths, death, yellow meat, fever, and loss of appetite.

Enumerator: signs and symptoms in humans bloating of the stomach, fever, vomits, death, and loss appetite

Enumerator: we now move to our next question. My question is how do human beings and livestock get Rift Valley Fever?

R3 How does someone gets affected?

Respondent 3: when s/he is bitten by a mosquito.

Respondent 1: he gets affected when bitten by a mosquito.

Enumerator: when the mosquito bites him. Any other way someone will contract the disease?

Respondent 4: when mosquitos bite you.

Enumerator: without a mosquito bite is there another way someone will contract the disease?

Respondent 1: when a mosquito bites the livestock, and you consume the livestock products like meat and milk that’s when you will contract it.

Respondent: you will contract it when you drink the milk and eat the meat.

Enumerator: how do livestock gets affected?

Respondent: 2 when livestock is bitten by mosquitos and kala-azar.

Enumerator: is mosquito or kala-azar? R5

Respondent 5: mosquitos

Enumerator: how did you know that someone is affected by Rift Valley Fever and with all signs that you gave me?

Respondent: 3 when there is an outbreak. During the rainy period, livestock will move to where there is water and they spread. Later the veterinary officers will tell us not to consume anything from the livestock since there is an outbreak of RVF

Respondent: 6 hospitals.

Enumerator: R1.

Respondent 1: you will be examined, and you will be told about the disease by the medical officer

Enumerator: our fifth question is, how do you treat those who are affected by Rift Valley Fever?

Respondent: 4 we take them to the hospital.

Enumerator: R4.

Respondent 4: we take them to the hospital.

Enumerator: is it a private or government hospital?

Respondent: 1 Whichever is near you be it private or government hospital.

Enumerator: mostly you take them to which hospital?

Respondent: 2 The government hospital.

Enumerator: is there another way you treat the person without taking him to the hospital?

Respondent: 3 we give them traditional medicine.

Enumerator: which are these traditional medicines?

Respondent: 4 *bires* if he has yellow fever.

Enumerator: I mean Rift Valley Fever. R8 what are the other traditional medicine you give to the affected person?

Respondent 8: *waldena*

Enumerator: is there another way you treat the affected person?

Respondent: 5 how can you treat yourself? It’s your family who treats you.

Enumerator: What are the other measures you put in place to prevent Rift Valley Fever?

Respondent 8: you put mosquito nets.

Enumerator: what else?

Respondent: 1 clearing the bushes and burning dirt.

Enumerator: what else?

Respondent: 2 covering water containers and cutting grass.

Enumerator: what else?

Respondent: 4 draining stagnant waters.

Enumerator: What else?

Respondent: 3 buying mosquito repellants drugs.

Enumerator: what else?

Respondent: 5 spraying insecticides and pesticides in the cowsheds.

Enumerator: what else?

Respondent: 6 applying oil on the livestock body.

Respondent: *chorus responses* all we know is that if you know anything else you can tell us.

Enumerator: does vaccination help the livestock?

Respondent: 7 we get vaccination once in a blue moon, but we also buy and vaccinates our livestock.

Enumerator: so, vaccination doesn’t help?

Respondent: 8 Majority of us don’t know anything about vaccination.

Respondent: 1 Not every livestock gets vaccination.

Enumerator: does it help livestock that gets vaccination?

Respondent: 2 it helps.

Enumerator: is there another way you prevent this disease? You said you get from milk?

Respondent: 4 Not everyone milks the livestock but those who have died we know they haven’t died by consuming livestock products.

Enumerator: any other way? R2 you told me earlier that you help livestock that have stillbirths and you can contract the disease.

Respondent 2: yes.

Enumerator: how do you prevent it?

Respondent 2: by wearing hand gloves and helping it. I have it.

Respondent: we make a local one and use it.

Respondent 2: you can look for it if you need it.

Enumerator: if we compare everything. Use of nets, clearing bushes, draining stagnant water, use of mosquito repellant drugs.

Respondent: 5 vaccinations can be helpful if given on time.

Enumerator: have you agreed that vaccination is the most effective?

Respondent: 4 Yes, vaccination is the most important.

Enumerator: why vaccination?

Respondent: 5 Those who have studied know the disease and they come to vaccinate the livestock.

Respondent: 6 to prevent the spread of the disease.

Enumerator: who makes the decision for the livestock to be vaccinated?

Respondent: 7 the father.

Enumerator: why father?

Respondent: 8 Because he is the household head, he makes the decision.

Enumerator: who takes for vaccination?

Respondent: 7 we all take to vaccination, if he asks for help we go together.

Enumerator: what’s next after vaccination? Use of nets, bush clearing, use of repellant drugs

Respondent: 6 spraying insecticides.

Respondent: 1 I think bush clearing is next because not everyone can afford to buy insecticides.

Enumerator: why bush clearing?

Respondent: 2 you can use your energy to do that.

Enumerator: who makes the decision for clearing the bushes?

Respondent: 3 nobody, that’s your willingness.

Enumerator: who in the household?

Respondent: 3 the wife.

Enumerator: if the wife makes the decision, who clears the bush?

Respondent: 5 Both the husband and wife can do it.

Respondent: 6 Not every household can have both couples some have fathers and some have mothers alone. Mother and her children can clear the bush if the household is headed by the mother.

Enumerator: what’s next?

Respondent: 8 we said number two is bush clearing.

Enumerator: the remaining ones area use of nets, spraying insecticides, use of hand gloves, applying mosquito repellants. What’s next?

Respondent: 7 use of nets.

Enumerator: why the use of nets?

Respondents: 2 everyone can afford the net. It is given to those who have children and pregnant mothers. There is no household that will miss mosquito nets.

Enumerator: who makes the decision for the use of mosquito nets?

Respondent: 3 There is no decision, that can be done by the wife/mother.

Enumerator: it’s one person in the household who will say you use mosquito nets.

Respondents: *all.* The wife

Enumerator: what next? The remaining ones are use of insecticides, applying mosquito repellants,

Respondent: 3 uses of insecticides.

Enumerator: who makes the decision for the use of insecticides?

Respondent: 2 household head.

Enumerator: why household head?

Respondent: 6 when I say household head, women can also be the household head.

Enumerator: if the household head makes the decision, who will spray the insecticides?

Respondent: 4 whoever have it will start.

Enumerator: is it men or women?

Respondent: 5 men.

Enumerator: why men?

Respondent: *6* Its men who mostly migrate with the livestock.

Enumerator: the remaining ones are the use of hand gloves and applying mosquito repellants. What’s next?

Respondent: now this one everyone can say which is next. Personally, I use mosquito repellants.

Enumerator: R2 tells us about the gloves.

Respondent 2: you must use gloves when your livestock has stillbirth.

Enumerator: who makes the decision for the use of gloves?

Respondent: 5 personally, I protect myself I have to use it. If it’s my children, I will tell my children to use it.

Enumerator: children touch the still birth?

Respondent: 6 yes, the teenagers.

Enumerator: they throw it away.

Respondent: 4 so I tell them to wear gloves.

Enumerator: who makes the decision on anything concerning the livestock?

Respondent: 1 if you have a husband, he is the one who makes the decision. I mean the one who have husband. They have discussion together, but he makes the decision. Their wife cannot make decision she just tells him that children need school fees. If the household is headed by a mother, she makes the decision. She cannot ask for a permission anything to do with chicken.

Enumerator: what about money?

Respondent: 2 the husband sells the livestock, and they will discuss what to do with the money which they can use for school fees and other for household needs.

Enumerator: so, money it’s the husband who makes the decision. Why is the father making decision on money?

Respondent: 4 we are talking about different household heads. If it’s fifty thousand, we can use this amount for school fees and also this amount for household needs and we can save this amount. She can ask what to do next if the money depletes.

Enumerator: maybe children are sick or even the parents, who makes the decision for someone to be taken to hospital?

Respondent: 5 you ask the father which hospital you can take the children.

Enumerator: why ask the father?

Respondent: 6 he is the head. He manages both human beings and livestock.

Enumerator: we now move to section C *inaudible conversation and laughing.* What are effects of the Rift Valley Fever?

Respondent: 7 it reduces the number of livestock. If you used to have fifty now you will have twenty. Since are pastoralists if our livestock dies, we also go into losses.

Enumerator: losses

Respondent: 2 if the herder gets sick you will have to use the money you have to take care of the affected person. Taking him the o hospital is another expense.

Enumerator: R3 what are the other effects of this disease?

Respondent 3: that’s it. There is nothing else.

Enumerator: What are the other effects of this disease?

Enumerator: R8 what are the other effects of Rift Valley Fever?

Respondent: 8 it causes death.

Enumerator: how does it affect men and women?

Respondent: 3 It affects all of us the same.

*Chorus conversation.* This disease affects women mostly because when all livestock dies the husband will move away since he cannot cater to the household needs so it’s the mother and children who will be affected. Children will ask for school fees, books and everything their mother and she doesn’t have everything she used to have since all livestock have died. It will lead to SGBV.

Enumerator: how will it affect the knowledgeable and unknowledgeable?

Respondent: 5 repeat it.

Enumerator: how will it affect the knowledgeable and unknowledgeable?

Respondent: 6 the knowledgeable know how to protect himself will those who doesn’t have the knowledge will just die with it without knowing.

Respondent: 7 for example, I will use protective gears when handling livestock but elders in our village they don’t know anything about it.

Enumerator: what about those who have resources and those who don’t have how does it affect them?

Respondent 5: the resourceful ones will go to hospital for treatment while those who don’t have resourceful will not be able to go to hospital they will just die with the disease.

Enumerator: how does it affect the old and the young, teenagers are included in the young.

Respondent: 4 young people have strong immune while old people have weak immune.

Enumerator: is there another way except the immune one?

Respondent: 3 the young ones might not be resourceful.

Enumerator: you told me how to prevent disease. We will just remind each other, why did you say vaccination?

Respondent:1 prevention is better than cure.

Enumerator: I will narrate a short story and am going to give you cards whereby we don’t talk but we use the cards to respond. There are two people, let’s listen carefully, they are husband and wife. The husband is called Boru, and the wife is called Amina.

Enumerator: There is Amina and Boru; they are husband and wife. They are pastoralist, they have cows, camels, goats and sheep. In the year 2023 there was an outbreak of disease that affects both human beings and livestock. We have understood each other up-to there. Enumerator: my first question is and remember we don’t talk we use the cards. How will the resource of Boru and Amina help in preventing the disease? Does Amina have the power to sell the livestock when there is an outbreak of disease? Or its Boru who have the power? Or its both of them? This picture is Boru, this is Amina, and this is both of them. Have we understood each other? Does Amina have power to sell livestock in case of an outbreak

Scores

Amina-0

Boru-7

Both-1

Reasons for both

Respondent 6: because they are one people and when one is not available one can make the decision.

Reasons for Amina

Respondent 5: he is the head of the household.

Enumerator: why is the head making the decision?

Respondent 5: because the buck stops with him.

Enumerator: R4 don’t repeat what she has said, why is Boru making the decision?

Respondent 4: he is the head.

Enumerator: R2.

Respondent 2: he is the head of everything. When there is a problem in the household, he makes the decision.

Enumerator: R1.

Respondent 1: he has all the household responsibilities that’s why he makes the decision.

Enumerator: R3 why does he make the decision?

Respondent 3: it’s his responsibility.

Enumerator: R8.

Respondent 8: because all responsibilities are on him be it livestock or the household.

Enumerator: sometimes people exchange livestock.

Does Amina have the power to exchange livestock? Or it’s Boru the husband who has the power or do they discuss it together?

Scores

Amina

Boru

Both-1

Reasons for both

Respondent:7 They discuss together.

Respondent:2 Maybe she wants a male one or a female.

Respondent 3: There might arise a misunderstanding if you are not going to tell him anything.

Enumerator: R4, what type of misunderstanding?

Respondent 4: he will ask you how you did that without telling me.

Enumerator: how will you solve the misunderstanding?

Respondent: 5 We will sit together and solve everything slowly.

Enumerator: what if you won’t be able to solve it?

Respondent: 6 you will add someone.

Enumerator: who is that someone?

Respondent: it can be a family member, elder, or even neighbor. You will them that you have a misunderstanding, and you want them to help you solve it.

Enumerator: can it surpass the elders?

Respondent: no, it won’t.

Enumerator: R5 why are they discussing together?

Respondent 5: they are one and the same thing.

Enumerator: R6 why are they discussing together?

Respondent 6: they are the same people.

Enumerator: any other reason?

Respondent: they are the same people. Anything won’t work without each other.

Enumerator: R1 why are they discussing together?

Respondent 1: they are the same people.

Enumerator: Does Amina have the authority to go hospital?

Scores

Amina-0

Boru-1

Both-7

Respondent: she can’t.

Enumerator: we use pop-the-cards.

Respondent: repeat it.

Reasons for Amina

Respondent 5: he is the household head, and nothing can work without him.

Enumerator: R6 why are they making decision together?

Respondent 6: the reason they are making decision together is that not every time that the father is around so I will have to take him to hospital unless when am told he is referred to other hospital and money is needed that’s when I will call him and inform him about it.

Respondent 2: because they are there for the wellbeing of their family and the livestock is theirs too. Livestock also belongs to the wife not only the husband, but they must also have discussion together for the betterment of their family. The husband cannot make decisions alone and wife cannot make decisions alone.

Respondent 1: she cannot go without him.

Enumerator: they have a small amount of money and Amina wants to start a business. Does Amina have the power to use the money to start the business? Show me with cards. Everyone raises the cards and don’t look at each other’s card. 6 Boru 3 Both. R6 Why can Boru make the decision alone?

Scores

Amina-0

Both-6

Boru-2

Reasons for boru

Respondent 6: he is the one with livestock, so as Amina, I will have to call him and tell him about the business idea I have so that he can sell the livestock and send me the money. Without him, I cannot take the money and start the business.

Enumerator: R1

Respondent 1: without him, I cannot start the business.

Enumerator: why can’t you start the business?

Respondent 1: he is the head.

Respondent 9: he is the owner. Maybe I inquire into loss and when I want help maybe I won’t be able to get it.

Reasons for both

Respondent 4: they must share everything together

Respondent 2: business is part of the development, and they have to share the ideas. Want is your suggestions I want to start business. If it’s something that will uplift the family status, he must have said in it.

Respondent: 5 they are one and same thing. Livestock belongs to both of them so any development they have to discuss together. It’s not must husband to know everything there are other wives who are also bright.

Enumerator: I will narrate another short story. There are two people who are husband and wife. The husband is called Adan who is 45 years old, and his wife is known as Sharifah who is 40 years old. They have been married for three years. They are pastoralists. They have Cows, camels, goats, and sheep. In the last four years, there was a disease that keeps occurring. Sharifah was invited to the seminar so that she can tell them about the disease in their area and to gain knowledge and create awareness. The cards have changed, this is Sharifah and Adan.

Respondent: Sharifah and who?

Enumerator: Sharifah and Adan. And this is Sharifah alone and this is Adan alone. Does Sharifah have the power to go to the seminar? Or it’s Adan have the power? Or they will discuss it together? Show me with cards.

Can Sharifah make a decision to go to the seminar?

Scores

Both-7

Adan-1

Sharifah-0

Reasons for Adan

Respondent 5: he wants the knowledge, and he doesn’t have the livestock. He wants her to attend the seminar to gain knowledge and come back to educate the community.

Reasons for both

Respondent 2: she asked, you cannot go without his permission, if he declines what would you do?

Respondent 9: she cannot go until they have a discussion with the husband.

Respondent 6: they are same people, and the disease will affect both of them, one person have to go to seminar.

Respondent 6: he must give her the permission.

Respondent 1: they are same people why will have a discussion.
