# Supplementary material for: “Without a man’s decision, nothing works”: Building resilience to Rift Valley fever in pastoralist communities in Isiolo Kenya
Source: PLoS One. 2025 Jan 28;20(1):e0316015. doi: 10.1371/journal.pone.0316015 (PMC11774392; doi:10.1371/journal.pone.0316015)
Supplement: S1 Dataset — (ZIP) [file pone.0316015.s001.zip › Supporting Information Files/File 11.docx]

**E: Which types of animals do you keep? Raise your voice when you speak.**

R1: Cow, goats, chicken

**E: Is there any other animal that has not been mentioned?**

Chorus: Donkey.

R4: We don’t have camels.

**E: Do women own livestock?**

R7: Women have livestock.

**E: Which types of animals do they have?**

R7: Donkeys, goats and some own cows.

**E: Where do they get the animals from?**

R7: When they get married, they are given as bride price.

*(Birds chirping)*

R2: The person who is in charge of our welfare is a woman. I own cows, goats, and donkeys. When I got married to my wife, I married her because of the livestock. When we go into the livestock business, whatever money we get, we hand it over to our women. Even if I go as far as Isiolo to sell the animals, when I get the money, I hand it over to her. Sometimes I can take out some money and buy something for the family, but the rest of the money is given to her. She runs everything in the household. But there are women who are not straightforward and can mess up the resources she is given. If you get a good wife who has borne children for you, everything belongs to her. We refer to her as the mother of the house. Sometimes if a man wants to be made an elder, and there are some qualities that he lacks, the other elders will look at his wife and if she is seen as hardworking, generous and respectful, the man can be accorded the position because of her.

**E: Whom does the chicken belong to?**

R3: It belongs to the mothers and their children.

**E: What about donkeys?**

R4: It is hers; she uses it in doing her house chores.

**E: Which animals are known to be owned by men?**

R2: What is known about men in the past, not the men who have gone to school is going out to get livestock. I have never gone to school. We used to go and fight other men and animals and it is the women that take care of our families.

**E: Okay. So which animals are known to be owned by the men?**

R2: Cows belong to men. Goats, sheep, and donkeys belong to women. No one can sell the cows if the father has not authorized it. A woman can consult her children and can sell the goats. It is not a must for us to be involved.

R3: Cows are animals that are known to be owned by men. Goats and sheep belong to women and children.

**E: What are the common diseases that affect humans and livestock in your community?**

R4: There are so many sand flies in the region and they really bite us. There are so many mosquitoes too. There were mosquitoes during the drought season and now they are more because of the rains.

**E: R5, tell us about the diseases that affect animals.**

R5: Tryponosomiasis.

R6: Tick infestation.

R1: Fever and typhoid.

R4: Yellow fever.

R5: RVF fever.

Chorus: CCPP.

**E: Does the RVF disease affect humans?**

Chorus: Yes, it does.

**E: Is there a difference between yellow fever and RVF disease?**

R4: There is a mix-up in these diseases, in the past as we moved with our animals, the main type of disease we used to encounter is trypanosomiasis which was caused by an insect. When it bites an animal, we use a red kind of medicine that is made into a solution and the infected animal is injected with it. These days there is this mathenge tree which has caused a lot of harm to our animals. Even the grass that grows under that tree is dangerous to our animals. It has brought about so many diseases. Even the mosquitoes breed in that tree. In the past, the disease that affects us is TB which infects a few people. If the infected person dies, we used to burn that house down. These days there is cancer which is brought by the mathenge tree.

**E: Do you know about RVF disease?**

R4: This is caused by mosquitoes. It affects all the animals.

R5: It is caused by mosquitoes; you get fever and then diarrhea. Some survive after diarrhea and others die.

**E: Do you understand the RVF disease?**

Respondent: All, yes.

**E: In our subsequent discussions, we shall be discussing the RVF disease. Don’t get confused by the other diseases.**

**E: What are some of the symptoms of the RVF disease in animals?**

R1: It has goosebumps, stands alone, and does not eat.

R5: When you see the animal in that condition, people who know say that it has been infected by the disease and find its medication.

**E: Is there any other symptom?**

R2: I used to look after European animals, I worked there for a whole year. I learned that if an animal who has goosebumps and doesn’t eat does not get immediate medication, the animal dies. I can easily tell an animal who has been infected.

R4: Its urine color changes from yellow to brownish. It also has heavy saliva.

R2: Don’t mix the symptoms, we are talking about the RVF disease.

R4: The symptom is yellow fever.

R2: Yes, we don’t want to discuss yellow fever but RVF disease. The lady wants us to discuss the RVF disease which kills both humans and animals.

**E: Is there any other symptom of RVF disease?**

*(Men discussing)*

R5: The animal dies.

R1: Reduction of milk production.

**E: Does the animal aborts if it is infected by the RVF disease?**

Chorus: Yes, it does abort.

**E: What about the humans?**

R3: You feel nauseated and experience headache.

R1: You feel nauseated and vomit.

**E: Is there any other symptom?**

R4: You feel hot, your temperatures are high, and you start sweating a lot.

R5: After eating meat from an infected animal, you will have headache, fever and you start to bleed from mouth and nose.

*(Birds chirping)*

*(Men talking)*

*(Chicken clucking in the background)*

**E: signs and symptoms of RVF disease in humans.**

R2: Bleeding from mouth and nose and vomiting. When blood starts to come from your mouth and nose, there is not much hope of staying alive.

R4: Bleeding because another type of infection can make you vomit.

R3 death

R7: Fever.

R5: Fever.

R6: headache

R1: Headache can be a symptom of any infection.

**Signs and symptoms of livestock**

R1: Loss of appetite.

R4: 4Abortion.

R3: The teary eyes show that it has been infected.

R2: The death of the animals.

R5: Milk reduction

R7: Abortion.

R6: Death.

**E: How do humans and animals get infected with the RVF disease? How do humans get infected R3?**

R3: They get infected from being bitten by the mosquitoes.

R4: From drinking milk is from an infected animal.

R5: From eating meat that is from an infected animal.

**E: How did you learn that an animal has been infected by the RVF disease?**

R2: We get infected mainly from drinking milk and eating meat. We don’t normally boil milk. We drink the milk raw. If the animal is infected, you will definitely get infected and eventually die.

R4: We slaughter animals who are almost dying, and they might have been infected by the RVF disease. We eat the meat and that is how we get infected by the RVF disease. Lack of knowledge is what is killing us.

**E: You have diverted a bit from my question. My question is how did you learn the infection was from the RVF disease and not any other disease?**

R4: We are a community that depends highly on meat and milk. When the disease infects the animal, we know that the disease can be transmitted through milk and meat and that is how we know.

R3: We know about the disease because the animals will show the symptoms. From the symptoms, we know that the animal has been infected. Sometimes we give the animals medication but there is no positive outcome.

**E: How do you treat people who have been infected by the RVF disease?**

R2: We used to give them herbal medicine but these days we take them to the hospital.

**E: Is the hospital private or public hospital?**

R5: It depends with hospital which is near you and also if you have money.

R2: If you have money, you can go to a private hospital.

**E: Is there any other way of treating the sick? Do you use herbal medicine?**

Chorus: Yes, we do.

R4: There is an herbal medicine from Kisii which I don’t know the name which cures the RVF disease.

R5: There are certified herbalists who sell medicines that are very effective.

R1: There are borana herbs, but we cannot really rely on them because no one has ever gotten a cure from them.

R2: In any emergency case, a patient can be picked up using an ambulance but the problem we have with our hospital is the lack of medicine.

*(Men discussing)*

R3: There are many herbs like *walthenna*, *mwarobaini* and also *arsa* but all these are not effective.

**E: Other than going to the hospital to seek medical attention, how else do you prevent yourselves from getting infected by the RVF disease? R3, you earlier mentioned that you put on protective gear when milking and helping an animal give birth, right?**

R3: Yes

**E: How else can you protect yourselves from getting infected?**

R3: Boiling of milk before drinking.

R4: Cutting the meat and washing your hands well. Bring the meat to boil then pour out the soup. Add water again then bring to boil. You can eat the meat after it is well cooked.

R1: Put lids on top of all the open water containers which have water.

*(Birds chirping)*

R6: We move the animals to a different area away from the infestation.

**E: Do you get vaccinated got animals?**

*(Men speaking in low tones)*

R3: It is not the vaccine that is the problem. The problem is the lack of veterinary doctors who could advise us better.

R2: What R3 is saying is a past thing. These days, there are no vaccines. The government has not sent anything our way in quite some time.

**E: Of all the preventive measures we discussed, which is the most efficient?**

Chorus: Cleanliness.

**E: Which is?**

R2: Put lids on open water containers, and clean the environment to prevent breeding of the mosquitoes.

**E: Why would you do that?**

R2: Mosquitoes and flies don’t breed in a clean environment.

**E: Who authorizes the cleaning and covering of open water containers?**

Chorus: It is the mother.

R5: It is the father.

R1: It is the mother because she is responsible for the house chores.

R5: Yes, she does the work, but the father is the one who gives directions on how things are done in the household.

**E: Why him?**

R5: Because he is the man of the house, and the family is his responsibility.

R2: The woman stays at home and carries on with her daily routine but the father walks around town and he might get information in case there is a disease outbreak, and he can come back and pass the information about the disease and how they can prevent themselves from getting infected.

**E: Which measure follows cleanliness?**

R5: Boiling of milk and proper cooking of meat.

**E: Why?**

Chorus: That is what we depend on as our main food.

**E: Who gives authority for that to be done?**

R2: The father directs the mother on what to be done because the children don’t know what is safe to be drunk or eaten so it is her responsibility to ensure that the children eat safe food.

R1: The father is the one who takes care of the animals so he can differentiate a sick animal from the rest so he knows where the meat and the milk come from.

**E: Which measures follow that?**

*(Birds chirping)*

R4: Putting on gloves while helping the animals give birth.

**E: Why would you put on gloves?**

R4: To protect yourself from getting the infection.

**E: Who authorizes the use of the gloves?**

R2: The father

**E: Why him?**

Chorus: He is the head of the household.

**E: Who puts on the gloves the most?**

R5: Everyone. When the woman, man, or children go to milk the animals.

**E: Who assists the animals to give birth?**

Chorus: Men.

**E: Why not women?**

R8: women fear.

**E: What is the importance of changing cowshed as a measure of preventing the animals from getting the infection?**

R2: To maintain their cleanliness.

**E: Who gives authority of changing the shed?**

R4: The father.

**E: Why him?**

R4: It is his responsibility.

**E: Who changes the shed?**

R6: The father.

**E: Why can’t a woman do that work?**

R4: she doesn’t have the knowledge and the strength to do that.

R1: The household chores are too many she has to concentrate on that.

**E: Who makes the decision on livestock like selling the animals?**

R7: The father.

**E: Why him?**

R7: The animals belong to him.

**E: Who makes the decision on how money is going to be spent?**

R-All: The father.

**E: Why the father?**

R5: It is his responsibility.

**E: What about the decision of going to the hospital?**

R5: The father.

**E: Why him?**

R5: Because he is responsible for the well-being of his family.

R7: The hospital is the responsibility of the woman. Because, if it is the children, the woman is the one who takes care of them and takes them to the hospital.

R1: Some families are led by mothers, and it is their responsibility to take the children or themselves to the hospital.

**E: What are some of the effects of the RVF disease?**

R7: Poverty

R5: Becoming sick.

R1: Hunger.

R3: You need to go to the hospital, but you don’t have money.

R5: Loss of lives and children are left to be orphans.

**E: How does the RVF disease affect men and women differently?**

R3: A woman might be pregnant, and her blood level might be low. Because she cannot eat meat and liver which can boost her blood level, she might end up losing her child or her life.

(Cock crowing)

**E: We will use these cards to answer the questions. There are two people. They are husband and wife. The husband’s name is Boru, and the wife’s name is Amina. They own cows, goats, sheep, and camels. In the year 2023, there was a disease outbreak that affects both humans and livestock. My first question is, Can Amina sell the animals without consulting her husband? This card is Amina, this other one is Boru and this other one is both.**

*(Birds chirping)*

*(Men discussing)*

Will Boru make the decision to sell livestock, Amina or Both

Scores

Amina-1

Boru-7

Both-0

Reasons for Amina

R1: Amina is able to give authority because she is the one who handles all the household affairs. She looks after the children and ensures that they have gone to school.

*(Birds chirping)*

Reasons for Boru

R6: He is the father and the leader of the household.

R7: He is the owner of the livestock

R2: Everything concerning household ownership falls under the man’s authority.

R3: The husband is the one who makes the decision, he just shares his thoughts with his wife, but he is the one who takes the animals to the market. If it is taking the child to school, he is the one who will give his wife the money for school fees.

R8: It is the father who is responsible for the family and their resource. He makes all the decisions.

**E: Using cards to who has the authority over changing livestock?**

**Scores**

**Amina-0**

**Both-1**

**Boru-7**

*(Men discussing)*

Reasons for both

R3: They need to consult each other on that matter so that the husband doesn’t decide alone because if he makes a sole decision, there will be conflict and the conflict can only be resolved when he returns the livestock.

Reasons for Boru

R4: you cannot enter the house with the neck first. You have to use the head to enter a house likewise, women are necks in the households therefore they cannot make any decisions concerning household resources. Men make the decisions.

R8: He is the household head and all the decisions come from him, not the woman

R2: Men are the final decision makers simply because everything that happens within the household belongs to the man and not the woman

R3: We marry women and they come with nothing and because of that, they cannot make decisions on resources that they don’t own

R5: We men are the final decision makers in the household because even women belong to men and they are our responsibility, thus we cannot allow them to make decisions because they are under men.

**E: Scores on access to health services**

Scores

Amina-2

Boru-3

Both-3

Reasons for both

R1: Most of the time, it is the child who is sick, they have to consult each other on which hospital they have to take the child to because the children belong to both of them

R8: The man is concerned about the well-being of his wife and that is why they discuss the suitable treatment option

R7: Once you marry, the man and woman become one so all the decisions in the household have to be decided amongst themselves.

Reasons for boru

R6: He is in charge of the house. Their well-being is his responsibility.

R3: It is the husband who will give out money for the hospital, so he is the one to decide whether they go to the hospital or not.

R4: All family members are under the man because he is the household head. Therefore, all house decisions are made by him

Reasons for Amina

R5: Women also own livestock that was given to them during marriage. Because of this reason, if a woman falls sick and wants to go to the hospital, she can decide to sell her livestock to solve the problem.

R2: When men grow old, they often depend on women and all decisions come from the woman because as a man you no longer have the energy to do a lot of things

**E: Scores venture in other business**

Scores

Amina-3

Boru-

Both-2

Reasons for both

R3: It has to be discussed because both of them have responsibilities over their families. The husband is looking after the animals while the mother takes care of the household chores. They have to plan.

R1: They have to make a decision on who will take care of the business and running of the other chores.

Reasons for boru

R2: When I sold the animals, I had decided on what to do with the money. The money is given to the woman to run the business because men don’t value businesses like women do.

R4: The man is the leader of the house, and he is the one who makes all the decisions. He makes the decision for the business to be started and the woman can start.

R6: If the woman is a divorcee or a widow, she is at liberty to do what she wants to do. But if a woman is married, she has to consult her husband to start her business.

Reasons for Amina

R5: when it comes to business, women are best at it because men don’t take good care of income as compared to men. So, if there is an outbreak and we need to venture into business, women are in a better position.

R8: Yes, women can be allowed to open other businesses so that the family doesn’t lose all the livestock and resources. As men are looking after the remaining of livestock, women can open small shops to look after the family

**E: I have another short story. There are two people who are husband and wife. The husband’s name is Adan, and he is 45 years old his wife’s name is Shariffa and she is 40 years old. They are livestock keepers. They own cows, goats, sheep, and camels. There has been a disease outbreak that affects both humans and animals. The disease has been recurring for the past four years. Shariffa has been invited to attend a seminar about the disease. My question is, *can Sharifa attend the training without consulting her husband? Does Adan decide or do they decide together? Use the cards to answer the question.***

*(Men discussing)*

E: Decisions on participation in seminar/training on RVF

Scores

Sharifah-1

Adan-1

Both-6

Reasons for both

R6: She has to consult her husband before leaving for the training.

R3: She has to ask for permission from her husband to go to attend the training. If it is something that is beneficial to her, she will be allowed to go.

R1: As Muslims, they have to sit and discuss. There is no way a man can make the decision on her behalf, and she cannot make any decision without her husband’s consent.

Reasons for Sharifah

R2: I choose her because it is something beneficial to her and to the family. She is leading in everything in society.

Reasons for boru

R4: Adan is the household head and he decides whether the wife should go for seminars/ any journeys or not

**E: Do you ever get invited to attend seminars?**

R2: No, it is not conducted in our community but in other areas, but they pick a representative.

**E: Between men and women, who attends these seminars the most?**

R1: Women

R4: Men.

**E: Why women?**

R4: Men are not really interested but women love to learn, they want to be knowledgeable.

R3: Women share the knowledge with the rest of the community. Men will talk among themselves it does not go further than that.
