# Supplementary material for: “Without a man’s decision, nothing works”: Building resilience to Rift Valley fever in pastoralist communities in Isiolo Kenya
Source: PLoS One. 2025 Jan 28;20(1):e0316015. doi: 10.1371/journal.pone.0316015 (PMC11774392; doi:10.1371/journal.pone.0316015)
Supplement: S1 Dataset — (ZIP) [file pone.0316015.s001.zip › Supporting Information Files/File 12.docx]

Enumerator: what livestock do you have? You will say your name. R8

Respondent 8: we have goats and cows. Donkeys.

Enumerator: is there another livestock?

Respondent: 8 except goats and cows?

Enumerator: yes.

Respondent 8: I don’t know, you can ask others.

Enumerator: is there another livestock R6?

Respondent 6: we have hens.

Enumerator: is there another livestock? R2.

Respondent 2: I don’t have anything. we have goats, cows, and hens.

*Phone vibrating*

Respondent: 4 there is a donkey

Enumerator: do women own livestock?

Respondent All: *chorus responses* they have.

Enumerator: tell me one by one.

Respondent 8: they own

Enumerator: they have which livestock?

Respondent 4: they have cows, goats, and hens.

Enumerator: where do women get it from or how do they get it? R6

Respondent 6: some are dowries and some we are given by relatives, that’s which give birth to each other.

Respondent 5: by doing business you can buy like two or three and then this multiplies.

Enumerator: how about men?

Enumerator: R8 you have said you have cows, goats, and donkeys. Men own which livestock?

Respondent All: *chorus responds* they own everything.

Enumerator: you name it, please.

Respondent 3: they have cows, goats, and sheep, and they have money because some have salaries.

Enumerator: my first question is, which disease affects both human beings and livestock?

Respondent 8: livestock has died mostly because of this fever.

Enumerator: can you explain this fever?

Respondent 8: I have not examined to know but the fever is Rift Valley Fever, kalazar.

Respondent 7: Rift Valley Fever, kala-azar

Enumerator: these are two fevers.

Respondent 7: Rift Valley and Kala-azar. The other one is fever from *Mathenge*

Enumerator: is there another disease? R3

Respondent 3: The diseases are fever and kala-azar. Livestock dies mostly because of these two diseases.

Enumerator: there is *frosti,* is there another disease?

Respondent 7: there is *gasdor* which affects goats. You will just see goats dying. They have a fever and after two hours they die, and this mostly affects goats.

Respondent 6: There is vomiting and diarrhea in humans.

Enumerator: those who have not talked can now talk, R4.

Respondent 4: there is stomachache and flu among the people.

Enumerator: R7 is there another disease in livestock?

Respondent 7: goats and cows have a fever but the most affected is the donkeys.

Enumerator: it’s affected by which disease?

Respondent 7: it’s called *frosti.*

Enumerator: from trees?

Respondent 7: yes.

Enumerator: R9, is there another disease that affects human beings and livestock?

Respondent 9: there is Coenurosis.

Enumerator: you have mentioned Rift Valley Fever, does everyone knows it?

Respondent All: yes, we know.

Enumerator: when we are having a discussion do not confuse Rift Valley Fever with Kala-azar.

Respondent: *chorus conversation.*

Enumerator: how would you know when livestock is affected by Rift Valley Fever?

Respondent 8: when the livestock is affected with rift valley fever, first there is diarrhea, running nose, shivering, watering eyes, and fever.

Enumerator: anything she hasn’t mentioned?

Respondent 1: when livestock is affected with rift valley fever there is stillbirth in livestock.

Respondents 2,6, and 4: stillbirth usually occurs in large numbers.

Enumerator: anything else?

Respondent 8: goats can stay up to three years without giving birth when it’s affected by Rift Valley Fever. It affects the number of livestock since there is no increase.

Enumerator: any other effects?

Respondent 8: fever and delayed conception.

Enumerator: what about human beings? How do you tell that humans have RVF

Respondent 8: mostly we are with livestock outside, nets cannot even prevent the mosquitos.

Enumerator: what are the signs of RVF in human beings?

Respondent 8: vomiting, diarrhea, high fever, lack of appetite.

Enumerator: R8 when I ask the question you answer the last one, the rest are looking at you they don’t talk.

*Murmuring.* Is it okay?

Respondent 8: it’s okay.

*Coughing, door opening, the child crying.*

Table showing signs and symptoms of RVF in animal

|  | Diarrhea | Fever | Mocus from the nostril | Lacumation | Abortion | Infertility | Scores | Ranks |
| --- | --- | --- | --- | --- | --- | --- | --- | --- |
| Diarrhea |  | Fever | Diarrhea | Diarrhea | Abortion | diarrhea | 3 | 3 |
| Fever |  |  | Fever | Fever | Abortion | Fever | 4 | 2 |
| Mocus from the nostril |  |  |  | M.N | Abortion | M.N | 2 | 5 |
| Lacumation |  |  |  |  | Abortion | Lacumation | 1 | 5 |
| Abortion |  |  |  |  |  | abortion | 5 | 1 |
| Infertility |  |  |  |  |  |  | 0 | 6 |

Enumerator: you have highlighted that someone is affected with Rift Valley Fever when he i) losses appetite, followed by ii) vomiting, fever, and lastly iii) diarrhea.

My second question is asking, how do people and livestock get affected by Rift Valley Fever?

Respondent 4: when bitten by a mosquito.

Enumerator: R5 when do people get affected by Rift Valley Fever?

Respondent 5: when there are a lot of mosquitos that’s when he is affected.

Enumerator: except for bitten by mosquitos do people get RVF from different ways?

Respondent 6: the other fevers are different from Rift Valley Fever but once you are bitten by mosquito majority will be affected.

Respondent 8: As community we have mosquitos from January to December. During and night is just the same.

Enumerator: can someone be affected in another way without mosquitos?

Respondent 2: when you don’t clean your utensils, cover your containers, cleaning the dirt in the compound these also causes the fever.

Enumerator: how does livestock gets affected?

Respondent: 8 they get affected from the mosquitos once they are bitten.

Respondent 3: when bitten by the mosquitos.

Respondent 5: through the mosquitos.

Enumerator: How can human beings be affected by RVF?

Respondent 2: when livestock gets back from herding, we milk it and we drink the milk without boiling. When livestock dies from this disease, we slaughter it and consume the meat. Some people don’t even cook the meat properly that’s how people get affected.

Enumerator: how are humans/livestock with RVF treated?

Respondent 3: we go to the hospital and the doctors examine the affected person. That’s how we know.

Enumerator: do the doctor tell you that you have Rift Valley Fever?

Respondent 4: they examine your blood, and they say you have a fever.

Enumerator: I know but do they tell you it’s a Rift Valley Fever?

Respondent 5: no, they don’t.

Enumerator: so, they don’t say it’s Rift Valley Fever? How did you know?

Respondent 6: we know it's Rift Valley Fever because we bitten by mosquitos every time, so when we get sick in our mind, we just say it’s normal fever.

Respondent 7: it’s a big mosquito, it’s bites us daily. If you want to see it even now, it’s many in the nets.

Enumerator: mosquitos causing Rift Valley Fever are these big ones.

Respondent 8: *chorus responses* mmh.

Enumerator: how did you know that those big mosquitos are the ones spreading Rift Valley Fever?

Respondent 1: we believe that when it bites someone that’s when the fever is high.

Enumerator: R3 hat are you saying?

Respondent 3: you scratch your whole body.

**Signs and symptoms of RVF in human**

|  | Diarrhea | Fever | Mocus from the nostril | Lacumation | Abortion | Infertility | Scores | Ranks |
| --- | --- | --- | --- | --- | --- | --- | --- | --- |
|  |  | Fever | Diarrhea | Diarrhea | Abortion | diarrhea | 3 | 3 |
|  |  |  | Fever | Fever | Abortion | Fever | 4 | 2 |
|  |  |  |  | M.N | Abortion | M.N | 2 | 5 |
|  |  |  |  |  | Abortion | Lacumation | 1 | 5 |
|  |  |  |  |  |  | abortion | 5 | 1 |
|  |  |  |  |  |  |  | 0 | 6 |

Enumerator: How community to prevent the spread of the RVF disease? How do you prevent Rift Valley Fever?

Respondent: 3 there is no way to prevent it unless God does it for you. We go hospital.

Enumerator: I mean when you are healthy.

Respondent:4 we put the nets at night.

Enumerator: you put the nets. What else?

Respondent 8: you spray pesticides and insecticides in the household.

Enumerator: is there another way?

Respondent: 6 you clear the bushes and burn the dirt in the compound.

Enumerator: what about the livestock?

Respondent: 7 You spread insecticides.

Enumerator: you said you can be contract the disease from the livestock, how do you prevent it from spreading to human beings?

Respondent: 5 we don’t consume milk and meat.

Respondent 8: you stop the husbandry roles that may spread but since the livestock is yours there is no way you can leave it.

Respondent: 2 you cannot milk it until it gets better.

Enumerator: do you wear anything on the hands when handling the livestock?

Respondent: 1 we don’t wear anything on the hands we handle the livestock and then we wash our hands.

Enumerator: what about when you want to prevent the disease?

Respondent: 8 if are serious that we don’t what to be affected by the disease we wear gloves and mask but we cannot afford it. Hospitals cannot give you gloves and mask because you handling the livestock.

Enumerator: is there another way that we can prevent it?

Respondent: 7 there nothing else.

Enumerator: do you have something like vaccination?

Respondent: 7 previously they used to have vaccination.

Enumerator: does the vaccination help?

Respondent: 1 previously they used to help but now days there is no vaccination.

Enumerator: all these you have said, use of nets, clearing of bushes, spraying of insectides in the households, spraying of pesticides, not consuming meat and milk from the livestock, wearing of hand gloves, vaccination. If we compare everything which is the most effective?

Respondent: for livestock?

Enumerator: for both human beings and livestock. All those you have mentioned, which is the most effective?

Respondent: *chorus responses.*

Respondent: as R6 according to me the most effective is the use of mosquito nets.

Enumerator: why net?

Respondent: 6 to prevent the mosquitos.

Respondent 8: we use the nets at night in most households.

Enumerator: who makes the decision for the use of nets? R8

Respondent: 7 we use the nets because it prevents the disease.

Enumerator: who makes the decision for use of nets?

Respondent: All, we make our own decision nobody else makes for us.

Enumerator (Boru): Father or Mother?

Respondent: All, *chorus* the mother.

Enumerator: why the mother?

Respondent: 2 that’s the culture?

Respondent: 4 Father only looks for the food but the mother does all the household chores.

Enumerator: So, you make the decision for putting the nets. What’s next after use of mosquito nets?

Spraying of pesticides, clearing of bushes, not consuming milk and meat.

Respondent 8: we are done with nets?

Enumerator: use of gloves,

Respondent:8 for human beings and livestock?

Enumerator: for both.

Respondent 8: we are one and the same thing with the livestock, there are those which is around the area and the other outside. There is no where we can be separated from livestock, we cannot say vaccination is 100% effective.

Enumerator: then which is better for you?

Respondent: 5 vaccinating both human beings and livestock.

Enumerator: who makes the decision for the livestock to be vaccinated?

Respondent: 5 The father.

Enumerator: why the father?

Respondent: 4 he is the owner and is always around the livestock, he is with the livestock and during the vaccination, he is the one who takes it to be vaccinated.

Enumerator: those who haven’t talked can now talk because only two people are talking. What’s next after vaccination? We have removed net and vaccination. What’s next?

Respondent: cleanliness.

Enumerator: like what?

Respondent: clearing of bush and cutting of grass.

Enumerator: R6.

Respondent 6: cutting of the grass.

Respondent 8: draining of stagnant water after it had rained.

Enumerator: who makes the decision?

Respondent: 7 the mother, clearing of bush and draining of stagnant water.

Enumerator: why the mother?

Respondent: 7 because she is always around. The father’s job is to look for food and taking care of the livestock.

Respondent: 6 mosquitos bite the livestock and after two to three days there is sores in the mouth, then we spray pesticides and after another week we spray again.

Enumerator: who sprays the pesticides?

Respondent: 4 Father.

Enumerator: who make the decision to spray the pesticides?

Respondent: 4 he is the one who makes the decision.

Enumerator: what’s next after spraying the pesticides?

Enumerator: wearing gloves and not consuming meat and milk. Between the two which one is next?

Respondent 8: not to consume meat and milk from the affected livestock. When you realize that the livestock is affected you first inject it with antibiotics and when you read the instructions it has a duration in which you won’t consume meat and milk from the livestock.

Enumerator: you are not consuming meat and milk because of the injection or because of Rift Valley Fever?

Respondent: 1 we leave it because of Rift Valley Fever. You will inject antibiotics because of the other diseases.

Enumerator: the last one is wearing hand gloves. R5 why are you wearing hand gloves?

Respondent: 2 to prevent the disease that’s why I wear gloves.

Enumerator: to prevent the disease. Who makes the decision for you to wear the gloves?

Respondent: 3 The mother.

Enumerator: who makes the final decision?

Respondent: 3 mothers and father.

Enumerator: who makes the decision for exchanging the livestock, add the livestock or even sell it.

Respondent: 4 the household head.

Enumerator: R4 Why does the household head make the decision?

Respondent 4: he discusses with the wife what to do then he makes the decision.

Enumerator: who makes the final decision?

Respondent: 3 the household head.

Respondent 8: women also own livestock since they do business and they will reproduce, you will be given as a dowry or even by family. You will do want you want or you discuss with your husband what to do with it.

Enumerator: what about money? Let’s say you both have money, who makes the decision on what to do with the money?

Enumerator: can the husband make the decision alone?

Respondent: no, he can’t.

Respondent 8: when you have understating between each other he cannot make decisions by himself.

Enumerator: what will happen if he makes a decision alone?

Respondent: there will be conflict.

Enumerator: how will they solve the conflict?

Respondent 8: when there is conflict, you call the local elders and family, the one who have married you also has the family you call them and inform them.

Enumerator: can the matter be referred to someone else?

Respondent:8 no, it won’t.

Enumerator: what if he becomes hard-headed?

Respondent: 2 *chorus responses* you go to the kadhi court.

Enumerator: what next?

Respondent: 2 it won’t pass the kadhi court.

Enumerator: the other issue is when you want to take the children to the hospital, who makes the decision to go to the hospital?

Respondent: 3 the household head.

Enumerator: why the household head? R6

Respondent: 6 it is his responsibility.

Enumerator: R2.

Respondent 2: They will discuss and take the children to the hospital; one person cannot make the decision.

Section B

There are two people who are husband and wife. The husband is called Boru and wife is called Amina. They are pastoralists and own cows, camels, goats and sheep. In 2023 there was outbreak of disease in their area. It affects both human beings and livestock. I will ask you a question and please don’t talk we use the cards to talk. Have you understood my story?

Respondent: I haven’t understood.

Enumerator: have you understood my story?

Respondent: 2 There are two people, Boru and Amina. They have cows, camels, goats, and sheep. There was an outbreak of the disease in their area. Am I right?

Enumerator: yes. And I said this disease affects both human beings and livestock. Boru and Amina are husband and wife. They are pastoralists. Now, my first question is, how will the ownership of Boru and Amina help them prevent the disease? Does Amina have the power to sell the livestock? In this picture, this is Boru. *Laughing loudly.* I said don’t talk. Amina is this one and this Boru and Amina. Does Amina have the power to sell the livestock? If Amina has the power, you show me Amina’s. if Amina doesn’t have the power and Boru, has you raised Boru’s. If both have the power, you raise their card. Have we understood each other?

Respondent: mmmh.

Enumerator: Does Amina have the power to sell the livestock? Does she have the power? Everybody to raise the one is able. Where is yours?

Does Amina have the power? Everyone to show me the picture. 1,2,3,4 Amina. What’s your mama?

*Laughing loudly.*

Scores

Amina-3

Boru-3

Both-2

Reasons for Amina

Respondent 7: she is the household mother. She has the authority.

Enumerator: what about the household head?

Respondent:7 he has the power, but she has the authority because she takes care of the household responsibilities and because of these responsibilities, she can take the livestock to the market and sell.

Respondent 6: she equally takes care of the livestock, so she has the power to sell.

Respondent 8: she takes care of the livestock. She sells the livestock to take care of the household needs because children look up to the mother most of the time.

Reasons for both

Respondent 7: without discussion, no decision can be made because they participate in livestock rearing

Respondent 5: he owns the livestock, and he has responsibilities on his shoulder.

Reasons for boru

Respondent 4: he owns the livestock and takes care of it.

Respondent 3: he is the household head and am a wife, he brings the food and I do everything for them.

Respondent 1: because Amina has to understand that boru is the hh head and makes the ultimate decision.

*Inaudible conversation.*

Enumerator: Does Amina have the power to sell the livestock and go to the hospital? Don’t look at each other’s card.

Scores

Amina-3

Boru-3

Both-2

Reasons for both

Respondent 9: when she is sick the first person to be informed it’s him and he takes care of everything.

Respondent 2: when they won’t discuss nothing will move on. One person in the household cannot work alone.

Reasons for boru

Respondent: 5 The father is the final decision maker, he owns the livestock and if he decides to take them, I have no problem and I can also take them.

Respondent:3 This is because the man is the head, the sale of livestock brings about food and another home

Reasons for Amina

Respondent 6: she is the mother of the household. Children ask their mothers for everything e.g. fees, feeds. The woman also equally be in charge of livestock matters because she participates in all husbandry livestock affairs

Respondent 8: this is because women are looked up to in terms of food in the household than men

Enumerator: Does Amina have the power to exchange livestock? Don’t look at each

Scores

Amina-2

Both-0

Boru-6

Reasons for Amina

Respondent 6: Amina has power over the livestock that belongs to her and she can everything she wants.

Respondent 1: Because they co-own the livestock so she can also sell the livestock and attend to the needs

Reasons for Boru

Respondent: 4 he is the household head, and he can discuss with the wife then they can exchange but she cannot decide alone.

Respondent: 3 he owns the livestock, and he can decide anything. final decision is his.

Respondent:2 To avoid conflict in the household, the woman will allow him to make final decisions in the household

Enumerator: Does Amina have the power to sell the livestock and venture in other business? Don’t look at

Scores

Amina-0

Both-6

Boru-2

Reasons for both

Respondent 8: this is money and when you start a business, and you make a loss the blame starts coming but when you discuss and make a profit and loss, he is aware that will reduce complaints.

Respondent 2: this is because they co-own livestock so in case of any business, they have to consult each other.

Respondent 3: they discuss together to understand each other.

Respondent: 6 They do business together and share everything.

Respondent: 7 They will discuss it together; she won’t be able to use it without him because the business might backfire on you

Reasons for boru

Respondent: 4 A woman can’t do what a man hasn’t said. This is because women have less power to make decisions on the household asserts

Respondent: 3 he is the household head whatever he says that is what it will be, regardless of the discussion we hold together. He is the final decision maker

Respondent: 5 Yes, he makes the final decision. Whenever I want to go I won’t go without informing him.

Let’s listen I will narrate another story. There are other two people, they are husband and wife. The husband name is Adan and the wife is known as Sharifah. Sharifah is 40 years old and Adan is 45 years old. They have been married for the three years. They are pastoralists they own cow, camel, goats and sheep. There was an outbreak of disease which keep occurring after every four years. It affects both human beings and livestock. Sharifah was invited for a seminar so as to attend the seminar to gain knowledge and tell others about the disease in their area. Now we will use the cards. Does Sharifah have the power to attend the seminar? The cards have changed, this is Adan, and this is Sharifah and this is Adana and Sharifah. Have we understood each other? Does Sharifah have the power to attend the seminar? Can she make the decision alone? If she attends or not who makes the decision?

Scores

Adan-4

Sharifah-0

Both-4

Reasons for Adan

Respondent 5: Sharifah cannot go without her husband’s decision that’s why she informs him.

Respondent: 6 he doesn’t want you to go outside. you are his wife? Islamic culture won’t allow you to go outside alone.

Respondent 4: Adan is the household head you cannot do what he is not aware of.

Respondent 2: he is the household head. He makes the final decision. Other thing is respect.

Reasons for both

Respondent 1: she must request permission to avoid conflict. This is because in the Islamic religion, women are not allowed to move alone without their husbands. If a girl is not married, then she will have to move in with his brother.

Enumerator: As women how do have knowledge of Rift Valley Fever?

Respondent: All we have.

Enumerator: do you have knowledge about how to prevent RVF and how its spread?

Respondent:3,5,1 we don’t have the knowledge.

Enumerator: when there is training do women attend or its men only?

Respondent: 6 We have never been invited to a seminar.

Respondent: 2 Some are invited.

Enumerator: when invited is it men?

Respondent:8 mostly it’s men who are invited.

Enumerator: according to you why do they invite men?

Respondent: 7 it’s knowing each other.

Enumerator: beside knowing each other, why are the majority men and not women?

Respondent 8: they say women have no business to do with seminars and they go. Seminars have small tokens and they use that to buy miraa, they don’t even pass what they have gained from the seminar.
