# Supplementary material for: “Without a man’s decision, nothing works”: Building resilience to Rift Valley fever in pastoralist communities in Isiolo Kenya
Source: PLoS One. 2025 Jan 28;20(1):e0316015. doi: 10.1371/journal.pone.0316015 (PMC11774392; doi:10.1371/journal.pone.0316015)
Supplement: S1 Dataset — (ZIP) [file pone.0316015.s001.zip › Supporting Information Files/File 13.docx]

Enumerator: what type of livestock do you own?

R1 say your name and livestock that you own. R1 is your name.

Respondent: we own cows, goats, and donkeys.

Enumerator: anything else that she hasn’t mentioned and doesn’t repeat. R2.

Respondent 2: cows

Enumerator: do not repeat.

Respondent: 2 hens

Enumerator: who has said hen?

Respondent 2: me.

Enumerator: R2 has said hen.

Anything else? Livestock owned in the community are cows, hens, goats and donkeys.

Which livestock is owned by men and which is owned by female?

Do women own livestock? R1.

Respondent 1: Yes.

Enumerator: which one is owned by women?

Respondent: 1 the one which is mostly owned by women is donkey. (*Laughing)*

Hens.

Enumerator: what else? R6.

Respondent 6: Hen

Enumerator: which livestock is owned by men?

Respondent: 7 cows.

Enumerator: why is a donkey owned by women?

Respondent:7 it used for work like fetching water, carrying firewood.

Enumerator: R6 why are hens owned by women?

Respondent: 6 because it’s around the household and it cooked food in the household.

Enumerator: which livestock is owned by men?

R8, which livestock is owned by men?

Respondent 8: Cow.

Enumerator: Cows and what else?

Respondent: 2 sheep and goats.

Enumerator: why sheep and goats?

Respondent: 2 Women are considered to be weak, and they cannot go outside though there is time when they go outside, women can also own cows, sheep, and goats, and they also take care of it when it’s weak. He owns the livestock by virtue of being the household head.

Enumerator: we now proceed to our first question, which disease affects both livestock and human beings? Is there a disease that affects both livestock and human beings?

Respondent: all yes. (*Chorus)*

Enumerator: R3 tells one livestock disease.

Respondent 3: Fever.

Enumerator: Fever.

Respondent: 4 kalazar

Enumerator: which disease affects livestock?

Respondent: 5 Kalazar (*Chorus)*

Enumerator: R8 is there another disease?

Respondent: 2 There is GASDOR.

Respondent:5 Hoyale.

Enumerator: R5 Hoyale.

Respondent: it’s R4 speaking, there are so many livestock diseases, there is Gandhi, goats have gasdor, there is silisa, there is Hoyale, there is fever. During the rainy season there is a disease called yellow fever, whereby someone affected fever, bleeding the through mouth and nose.

Enumerator: which are these diseases that affect during the rainy period? R8

Respondent: R4, we just heard there is a disease called yellow fever you get affected from livestock.

Enumerator: is it yellow fever or Rift Valley Fever?

Respondent: 4 what is it called? Rift Valley Fever or what?

Enumerator: Rift Valley Fever is what you calling Yellow Fever?

Respondent: 4Yes.

Enumerator: so during rainy season you fear to be affected?

Respondent: 5 Yes. During rainy season there is disease called gasdor where goat have light waste faeces. There is silisa where it snores and have running nose throughout, *laughing.*

Enumerator: what about human diseases?

Respondent 1: diarrhea and vomiting and headache.

Enumerator: diarrhea and vomiting and headache. R2.

Respondent 2: Fever

Enumerator: R3

Respondent 3: Diarrhea.

Enumerator: R4

Respondent 4: mostly its diarrhea.

Respondent All: human diseases that mostly affects us is diarrhea

Enumerator: the diseases that you mentioned earlier are it affecting human beings?

Respondent: 5 yes, it is. There was period people were affected.

Enumerator: R6 is there any other disease?

Respondent: 6 there is nobody to examine the livestock meat for us, so we were affected. We were told you contract Rift Valley Fever from livestock, so we were not allowed to consume livestock meat and milk.

Enumerator: is there other disease that affects human beings? You told us about Rift Valley Fever, R4, how would you know that the livestock is affected with Rift Valley Fever?

Enumerator: what are the signs?

Respondent: 3 livestock will have fever,

Enumerator: fever

Respondent: 4 it lacks appetite,

Enumerator: lacks appetite.

Respondent: 5 yes, then we will know it is Rift Valley Fever. They will have running nose, other are urinating blood.

Enumerator: you have urinating blood?

Respondent: 6 yes.

Enumerator: what are the other signs of Rift Valley Fever?

Respondent: 1 *talking in low tone* they always have fever.

Enumerator: fever, running nose, urinating blood, lack of appetite, what else? R5

Respondent: 8 currently the most occurring disease is *frosti.*

Enumerator: we have written *frosti.*  Do you know Rift Valley Fever?

Respondent: 2 yes, I know.

Enumerator: what are the signs of Rift Valley Fever on livestock?

Respondent: 6 they shiver and sleep throughout, when they are affected with Rift Valley Fever they die the following day.

Enumerator: what else? Does the livestock have stillbirths?

Respondent: 3 they have stillbirths.

Enumerator: what about human beings? What are the signs of human beings?

Respondent: 4 human beings die.

Enumerator: there is death.

Respondent: 5 kalazar.

Enumerator: kalazar? We are talking about the Rift Valley Fever. What are the signs?

Respondent: 6 bleeding through mouth and nose.

Enumerator: bleeding through mouth and nose. Is there anything else?

Respondent: *murmuring in low tone*

Enumerator: mmmmh???

Respondent: 1 *speaking in low tone.* Change in skin color to yellow.

Enumerator: the whole body or parts?

Respondent: 7 face changes the color.

Enumerator: is there anything else?

Respondent: R4 speaking, where we’ve mentioned cow, goats, sheep, hen and donkey, there is another livestock.

Enumerator: which one?

Respondent: 5 in a center called Rig there is someone who own camels.

*Murmuring in low tone.*

Enumerator: is there another sign?

Respondent: 8 for what?

Enumerator: for Rift Valley Fever.

Respondent: 8 Eyes turns to be yellow.

Enumerator: you said it’s the skin that turns to be yellow.

Respondent: 5 not only the skin but also the eyes. Even the urine is yellow.

Enumerator: is there anything else? Now let’s look this side, you have told the livestock that is affected with Rift Valley Fever, you have said it has fever, still birth isn’t it.

Enumerator: you know Rift Valley Fever when someone dies, secondly when bleeding through mouth and nose, next is yellow urine, yellow body and lastly when someone has yellow eyes.

Next question: How do livestock and humans get affected with Rift Valley Fever?

Respondent 1: if someone is not sleeping under treated nets.

Enumerator: you have said it’s caused by mosquito bite. Any other way someone can be affected and livestock to be affected?

Respondent 3: *low tone.*

Enumerator: raise your voice.

Respondent: 5 kalazar

Enumerator: Does kalazar causes Rift Valley Fever?

Respondent: yes.

Enumerator: R6

Respondent 6: when livestock are in watery area they are bitten by kalazar.

Enumerator: is there anything else without mosquitos?

Respondent: 2 they are bitten by kalazar.

Enumerator: How are human beings affected? Without being bitten by mosquitos.

Respondent:1 drinking milk from infected livestock.

Respondent: 4 drinking milk from livestock, consuming meat, that’s how human beings are affected.

Enumerator: how did you know its Rift Valley Fever? How did you know that someone with the signs is affected with Rift Valley Fever? R8 come closer. How did you know? Where did you hear Rift Valley Fever?

Respondent: 8 long ago someone was affected and was examined that’s how we heard.

Enumerator: R4

Respondent 4: we were told to boil the milk and not to consume from affected livestock.

Enumerator: vet officers came or it’s health officers?

Respondent: 6 vet officers, they came and vaccinated the livestock *inaudible chorus responses.*

Enumerator: so you have heard from vet officers? Is there anything else except vet officers?

R7 tell us where you’ve heard from which haven’t been mentioned by others?

Respondent 7: *responding in low tone.* No.

Enumerator: R2 anywhere else? R5 anywhere else?

Respondent 5: no.

Enumerator: my third question is, what livestock husbandry can expose pastoralists to the risk of RVF.

Enumerator: you’ve said you contract Rift Valley Fever from livestock? What are livestock husbandry that you do can make you be affected with Rift Valley Fever?

*Murmuring.*

Can we talk since you want to go? If you keep quiet time is also moving.

Respondent: 4 Guys you’ve heard let’s talk. Vaccinating the livestock.

Enumerator: during vaccination can the person contract the disease if the livestock is affected?

Respondent: 5 no you cannot be affected but you can be affected if the livestock that you have vaccinated during the day and the evening it dies and you slaughter the livestock and eat the meat.

Enumerator: who slaughters the livestock?

Respondent: 7 the owner. We don’t have butcheries around.

If the livestock dies we make it halal very fast.

Enumerator: I want to know who slaughters the livestock.

*Chorus responses.*

Respondent: 6 We can call for a man or we even slaughter it.

Enumerator: All of you, you don’t have a husband, so slaughters it for you?

Respondent: we call our brother, uncles.

Enumerator: 8 A man.

Respondent: 8 Yes.

Enumerator: why a man? Why are women not slaughtering?

Respondent: 8 reason is, like cows we don’t have the strength to slaughter it.

Enumerator: so you don’t have strength.

You are saying something R4.

Respondent 4: am saying we are not capable of…..

Enumerator: you are not saying that I want to hear what you were saying.

Respondent: 2 *swearing* walai have just said that way.

Enumerator: you were saying something about culture.

Respondent: 3 *murmuring* its true culture doesn’t allow. If you look at how do you think can slaughter it? Women are naturally weak so they are not capable. *Murmuring and chorus conversation.*

Enumerator: don’t talk when someone is talking.

Respondent: 5 even goats and sheep women cannot slaughter.

Enumerator: any other husbandry roles? One is slaughtering and eating the meat. Any other husbandry roles?

Respondent: 6 *inaudible responses.*

Enumerator: you haven’t said anything.  *Laughing.* How can you prevent this disease?

Respondent: 7 There is no other way.

Enumerator: is there another way to prevent the disease?

Respondent 6: it’s caused by mosquitos and mosquitos are net.

Enumerator: how do you prevent livestock?

Respondent 6: I spray using pesticides

Respondent: 8 You can also light a fire around the house or the kraal where the livestock and humans sleep to chase away the mosquitos

Enumerator: R1. Let’s talk one by one.

Respondent: 1 you light fire around the boma and used as a mosquito repellant.

Enumerator: is there anything else R2? How else do you prevent this disease?

Respondent 2: there is a medicine you apply on your body to act as a repellant.

Enumerator: You told me you boil the milk.

Respondent: 7 boiling milks

Respondent: 3 vaccinations but we sometimes we lack resources for vaccination and it can help.

Respondent 5: we boil the meat and pour the soup because it is perceived that the soup has the virus but after boiling the meat is okay to be eaten

Enumerator: is there anything else?

*Murmuring and laughing.*

Let’s not laugh. You said you sleep under mosquito nets, spraying pesticides, applying repellant on body, boil milk, vaccination and boiling the meat and pouring the soup, if we compare all which is the most effective for you?

Respondent:  *chorus* net

Enumerator: show me by hand those who say net.

Respondent: nets are useful.

Enumerator: If we compare net, spraying pesticides, applying repellant on body, boiling milk, vaccinating livestock which is the most effective?

Respondent 4: nets are used by those who are with livestock.

Respondent:2 no, it also can be used at home.

Enumerator: which is the most effective?

Respondent: 1 net

Enumerator: why net?

Respondent: 3 children can sleep under net.

Enumerator: who makes the decision on the use of mosquito nets in your household?

Respondent: 4 there is nobody who makes the decision because I’m head of the household.

Enumerator: as a household wife, you make the decision

Respondent:5 because when your children sleep under mosquito net there is nothing you fear but when there is no net you have to prevent mosquitos from the children.

Enumerator: why are you making decision as the wife and not the children?

Respondent 2: the mother knows more than them

Enumerator: is there other reason as to why the mother makes the decision?

Respondent: 1 she is the head.

Enumerator: you are R6.

Respondent: 6 she is the head and takes care of the place. It’s her and God.

Respondent: 7 she is the head so she makes the decision.

Enumerator: we are through with the net, what’s remains are spraying pesticides, mosquito repellant, boiling milk, vaccinating livestock, boiling meat and pouring soup, which is the most effective?

Enumerator: reasons for vaccination in livestock?

Respondent: 8 you want your livestock to be healthy.

Enumerator: who makes the decision to vaccinate the livestock?

Respondent: 2 if it’s the husband, he makes the decision. If the livestock belongs to wife it’s her who makes the decision.

Enumerator: now according to me.

Respondent: it’s us who makes the decision.

Enumerator (Boru): when you speak, talk about something in your household.

Respondent: 3 so according to my household, my children are young so I make my own decision.

Enumerator: why are you making the decision?

Respondent: 3 Am the household head. Am the husband and wife.

Enumerator: who takes it to vaccination?

Respondent: 5 I take my own livestock, I don’t trust anyone if it’s about vaccination. Men use force to lock women out taking livestock for vaccination.

Enumerator: reasons for boiling milk. Why do we boil the milk?

Respondent 6: to kill germs.

Enumerator: who boils the milk?

Respondent: 6 Wife

Enumerator: who makes the decision of boiling the milk?

Respondent: 2 Mother.

Enumerator: why the mother? R2.

Respondent 2: she is the husband and wife

Enumerator: reasons for choosing spraying pesticides, applying repellent

Respondent: 6 spraying pesticides.

Enumerator: R1 why are you saying spraying of pesticides?

Respondent: 1 the reason is that there are pests and houseflies, so spraying pesticides will help the livestock to be healthy.

Enumerator: who gives the decision for spraying the livestock?

Respondent: 3 the mother.

Enumerator: why the mother?

Respondent: 3 she is the head of the household.

Respondent: 4 spraying of pesticides will help in prevention of ticks.

Enumerator: what next, we have removed spraying of pesticides. Only two things are remaining, boiling of meat and applying of mosquito repellant. What are the reasons for boiling the milk

Respondent: 5 boiling of meat.

Enumerator: boiling of meat. R1 why boiling of meat?

Respondent 1: boiling of meat helps in killing of germs

Enumerator: killing germs. Who gives the person for boiling the milk?

Respondent: 8 mothers.

Enumerator: why the mother?

Respondent: 8 she is the household head so she knows everything about the meat.

Enumerator: and lastly is the use if mosquito repellants. Why do you use mosquito repellants?

Respondent: 7 to help keep away the mosquitoes from biting you

Respondent: 1 during the rainy season, there are mosquitos so we apply the repellant and also apply to our children. Since its not everyone who can afford the nets.

Enumerator: my next question is concerning the livestock, if its selling or exchanging the livestock or anything that concerns the livestock who makes the decision?

Respondent All: The mother.

Enumerator: why mother?

Respondent: 2 she doesn’t have anyone else to make decision with. She is the head.

Enumerator: she is the head and doesn’t have anyone else to discuss with.

Why is this side silent? R8, R7 who has the power?

Respondent:8 the mother.

Enumerator: why the mother?

Respondent: 8 she owns the livestock.

Enumerator: R7. Take clothes from your mouth. You have put down the paper now you are using clothes.

*Laughing*

Remove the clothes. Who has the power? Raise your voice.

Respondent 8: the mother.

Enumerator: why the mother?

Respondent 8:  *inaudible response*

Enumerator: why mother? R5.

Respondent 5: because it’s her responsibility and she is the husband and wife. She gives her children amount of money to use.

Enumerator: what about hospital?

Respondent: 4 she is the one taking her children to hospital but that’s when there was livestock market.

Enumerator: who makes the decision?

Respondent: All *responds* the mother.

Enumerator: why mother?

Respondent: All because she is the head.

Enumerator: now I will narrate a short story, let’s listen to each other carefully *murmuring*. Are you sick?

Respondent: yes, she has fever.

Enumerator: sorry.

*Murmuring and phone vibrating.*

I will narrate a short story lets listen each other, there is a person called Amina, Amina is 45 years old. Amina has children. Amina’s husband died 7 years ago. Have we understood each other?

Respondent: mmmmh

Enumerator: Amina has children, she is 45 years old and her husband died 7 years ago. Amina owns livestock, she is a pastoralist, and she has sheep, goats, and cows. In 2022 there the was outbreak of the disease in their area, which affects both livestock and human beings. Have understood each other?

Respondents: mmmmh

Enumerator: we have said there are how many people? We have said there is Amina. And Amina is 45 years old; her husband died 7 years ago. Amina owns livestock, sheep, goats, and cows. In 2022 there was an outbreak of disease in their area which affected both livestock and human beings. My question is, if I would ask a question don’t speak but we speak using the cards. This is Amina and this is her children, have we understood each other?

Enumerator: it means she has teenage children. There is an outbreak of disease, would Amina be able to sell her livestock or who will sell the livestock? Have we understood each other? We shall use cards to answer this question (one card represents three card mothers, and children, and the last card represents other household members

Enumerator: the person who would be able to sell, you will show me with cards. Are we together up to there?

Enumerator: she has adult children who can make decisions. She can make decision and they can also make decision. Does Amina have the power to take the livestock to market and selling?

Everyone to show the white card, if you want to say children you raise it this way.

Respondent: we show you this side only? We show you this side or this side?

Respondent: you have said I show you this white side?

Enumerator: this is the mother, this the mother and her children, they will have discussion together, have we understood each other?

Respondent: mmmmh

Enumerator: this is the mother and this is the mother and children. Does Amina have the power to sell the livestock? Show me yours I have raised mine. Does she have the power? Don’t look at each other’s.

Respondent: we put this one down?

Enumerator: yes. Everyone to show her card. 1, 2, *Murmuring* raise it mama. *Inaudible murmuring.*

Scores

Amina 6

Children 2

Other household members 0

Reasons for the children

R6 tell me why would Amina discuss with her children?

Respondent: 6 as she is getting old, her children are growing up. If her children are capable she can have discussion with them if they are trustworthy.

Enumerator: R4 why would Amina discuss with her children?

Respondent:4 children will complain if they are not involved in the decision-making process because the children do a lot of herding.

Reasons for Amina

Enumerator: R8 Why would Amina make the decision by herself without discussing with the children?

Respondent: 8 It’s her children. They are under her so she will the decision.

Enumerator: R1

Respondent 1: She is the head so she makes the final decision that’s why I said it’s the mother.

R2: Amina is the head of the household and all the decisions come from her

Respondent 1: she is the head, both husband and wife that’s why she makes the decision.

Respondent:4 She gave birth and raised the children up to now. Children are not the same nowadays, some even sell livestock without their parent’s consent.

Respondent: 5 she takes care of the children so she makes the decision.

Enumerator: my next question is does Amina have the power to exchange the livestock? Sometimes we do exchange the livestock, right? *Child* `*crying.* Does Amina have the power to exchange the livestock? Show me by cards, I have raised mine.

*Child crying* *and murmuring*.

If she has the power, show me the cards, if she discusses with the children, show me her and the children.

Raise the cards, if she has the power or if she discusses it with the children

Enumerator: put that red down. *Murmuring.* Now show me, put it this way.

*Child crying.*

Score

Amina 2

Discus with children 6

And other household members 0

Enumerator: R4 why would Amina make the decision alone?

Respondent 4: she has raised the children so she knows everything about them.

Enumerator: R1 you said this right?

Respondent:1 She is the household head

Enumerator: who has said this?

Respondent 1: it’s me alone.

Respondent 2: she gave birth to the children and she wants them to obey her that’s why the make decision together.

Reasons for together

Enumerator: R6 Why would Amina and her children make decision together?

Respondent 6: the reason they make together is that she has raised her children, when they grow up they also own livestock so they make decision together.

Respondent 1: the reason she discusses with her children is that she might die so she has to make her children know everything.

Respondent:2 no. there is another reason, tell her, when the father was alive, we used to discuss everything together.

Respondent: she asking the next question. *Child speaking* continue sister. Keep quite.

Enumerator: does Amina have the power to sell the livestock to go hospital, maybe it’s her children or even her, does she have the power? If she has the power, you show me, if she discusses with children you show me. Who has the power? Hold this way, now everyone to show me.

Respondent 2: she is the head that’s why she has the power.

Enumerator: R1

Respondent 1: she makes her own decision

Enumerator: R2 what are you saying? Why does Amina have the power?

Respondent 2: she is the household head; she doesn’t have anyone else to discuss with

Enumerator: R1

Respondent: 1 whatever she says that’s what would happen that’s why she has the power.

Enumerator: R3 why does she have the power?

Respondent 3: mother has the power.

R8 why does she have the power?

Respondent 8: she has raised her children and gives directives; she has the power.

Enumerator: does Amina have the power to sell the livestock and use the money to do business?

Respondent: *All said amina* has.

Enumerator: we don’t talk; we use the cards to talk. Just one card. Now everyone show me the cards. Everyone says Amina has the power. R5 why does Amina has the power to use the money to start the business.

*Murmuring and yawning. Child coughing and crying.*

Respondent: 2 She is sick.

Respondent 5: she uses the money to start the business and also to take her children to school and hospital.

Respondent 4: the reason she has the power, is that she has raised the children with the help of God, she takes her children to school and cares about everything, that’s why she has the power.

Respondent 2: she owns the livestock, and she is the father and mother.

Respondent 7: she is the head and also, she raised her children, took them to school and cooks for them.

Respondent 6: mother is the head.

Enumerator: now put down the cards, I will narrate another short story, we are almost through. There is a mother called Sharifah

Respondent: Sharifah.

Enumerator: yes. She is 50 years old, she widowed, okay?

Respondent: mmmh.

Enumerator: Sharifah doesn’t have husband, her husband died, Sharifah owns livestock, she owns cows, camel, goats, sheep. She inherited the livestock from her husband. After her husband died she moved to the husband’s family, have we understood each other?

Respondent: mmmh *murmuring.*

Enumerator: she moved to her husband’s family with her children. There was an outbreak of disease in their area, this disease affects both livestock and human beings. Sharifah was invited for a seminar; Sharifah was called for?

Respondent: Seminar

Enumerator: seminar; she attends the seminar and tells the disease in their area and also gets the knowledge about the disease. The cards I will give you is for the other people in her household

Respondent: other people in her household?

Enumerator: yes, like her husband’s family. We said after her husband’s death she moved to her husband’s family with her children. The other card it’s her and her children.

Respondent: her children.

Enumerator: the other is her, this other is her husband’s family. Does Sharifah has the power to attend the seminar? If she can you raise Amina’s card, you show me this. We’ve said Amina was invited for a seminar, now does she has the power to attend the seminar?

If she has the power it’s this, if she discusses with her husband’s family you show me this. If she discusses with her children, you show me this. Are we together? Amina, Sharifah has the power to attend the seminar? I have raised mine, show me yours. Don’t look at each other’s card you seem to look at each other. Now show me. Show me yours. Two other family. 1, 2, 3, 4. Which have you raised?

Score participation

Amina 4

Discus with children 1

And other household members 3

Reasons to discuss with other family members

Enumerator: why would she discuss with husband’s family

Respondent 5: they are same roles that’s why they discuss together.

Respondent: 6 yes, after her husband’s death she moved to other side that’s why they discuss.

Enumerator: R7 why would she discuss with them?

Respondent: 7 they have same roles.

Respondent:7 they are same people.

Reasons for Sharifah

Respondent 2: she is the head of the hh and does not need any one’s opinions.

Respondent: 3 she is not with the other family, that’s why she makes decision alone.

Respondent 4: They share the same compound but household activities. the reason why she makes decision alone is that mother to orphan children have their own ways. There are in-laws who can even steal livestock from you. You share same compound so as not to be disturbed by other people. So she makes the decision alone so that nobody can steal her livestock.

Enumerator: My question is do women have knowledge on Rift Valley Fever?

Respondents:1 most single woman are denied to attend seminars

Respondent: 8 They attend seminar and have knowledge.

Respondent: 6 we don’t have.

Enumerator: R4 told me you have knowledge

Respondent 1: we have heard some little knowledge. You remember the last meeting we had together. Now this is the second time am hearing about Rift Valley Fever.

*Chorus and murmuring.*
