# Supplementary material for: “Without a man’s decision, nothing works”: Building resilience to Rift Valley fever in pastoralist communities in Isiolo Kenya
Source: PLoS One. 2025 Jan 28;20(1):e0316015. doi: 10.1371/journal.pone.0316015 (PMC11774392; doi:10.1371/journal.pone.0316015)
Supplement: S1 Dataset — (ZIP) [file pone.0316015.s001.zip › Supporting Information Files/File 14.docx]

**E: What kind of livestock do you keep as community**?

Chorus: Cows

**E: Please let us speak in turns.**

R4: We keep cows and goats. When I say goats, I mean goats and sheep.

R6: Cows and donkeys

**E: Is there any other animal kept here?**

R6: Chicken.

**E: Is there any other thing?**

Silence.

*(Someone coming in and greats the men in Kiswahili)*

*(A lady also comes in and greets them)*

**E: We’ve started a bit early so when you want to speak refer to yourself as R8.**

**E: our first question was to find out what kind of livestock is kept here. Do you keep Camel?**

Chorus: No, we don’t.

**E: Among this livestock, which ones belong to men and which ones belong to women? Do women own livestock?**

R6: Yes, they do.

**E: Which livestock belongs to them?**

R6: The donkeys belong to them.

**E: Do they own other animals?**

R2: They own goats and cows.

**E: How do they own the animals?**

R2: Some buy others are given by their husbands

**E: R7, how do they own the animals?**

R7: They are given as a dowry when they got married. They are given one cow and it multiplies.

**E: How do men own the animals?**

R8: Men are also given by their parents, grandparents, and uncles. Some men buy themselves the animals.

**E: What animals are they given?**

R8: In our area, people mainly own cows, goats, and donkeys.

**E: Which animals do men mainly own?**

R8: Cows and goats.

*(Donkey brays from a far)*

R3: Men are mostly given animals by their parents as it was mentioned earlier. Sometimes you find a woman owns more animals than a man. The woman’s livestock can multiply more as compared to the men. Since they live together as man and wife, they put their animals together and in case they separate, everyone takes what belongs to them.

R4: I would like to take you back a bit. My brother here said that women mainly own donkeys. It is true but also, they own other animals. The chicken is mainly owned by women. Some men might inherit livestock from their parents, while others work and buy for themselves. There are others who cultivate crops near the swamps, sell the produce and buy themselves livestock. So whatever belongs to the husband also belongs to the wife if they understand each other well. What is hers is mine and what is mine is hers. That’s all that I can say.

(Phone ringing)

**E: Please let’s put our phones on silent mood.**

**E: What are some of the common diseases that affect both humans and livestock in this community?**

R8: There are a lot of diseases that affect the animals but the main one that kills the animals fast is called *Anthrax and Mange.*

*(Birds chirping)*

**E: Move closer please and when you speak, please raise your voice.**

*(Donkey braying)*

R3: Chicken belongs to both men and women.

R5: I would like to add on what R8 has said. Both humans and animals fall ill. Since the time I have known myself and I have been living here, I have seen animals getting infected by Anthrax, fever, Foot and mouth disease. These diseases are common to the animals. They infect the animals in different times. The disease that we are fighting and we don’t know what to do about is the one that comes from the mathenge tree. The disease is so bad it kills all kinds of animals. This disease doesn’t have any kind of vaccination like the other common diseases.

R4: I would like to add on what has been said. There are diseases like CCPP and CBPP. These diseases infect the lungs of the animals.

**E: R2, is there any other disease?**

R2: Mange

R7: There is a disease called Foot and Mouth disease which makes an animal’s Abortion. When animals are sick, they don’t eat grass or take water.

**E: Is there any other disease?**

R4: Yes, there is. When the animals start to fatten during the rainy season, they are infected by a certain disease which brings with it very bad fever. When you slaughter that animal, its meat is yellow in color. (Qando birte)

R6: We don’t know what happens to the animals sometimes it just falls down and die.

**E: I would like to ask you R4, is there a difference between qando birte and the RVF?**

R4: What I would like to tell you is that mosquitoes bite animals. There are two types of mosquitoes. One doesn’t bring any harm to the animals and the other ones which are as big as houseflies. The big ones are the ones that harm the animals.

**E: Are the big mosquitoes sand fly?**

R4: No, they are aedes mosquitoes. It brings about RVF disease too. It bites humans too and they die. Same as Sand flies they make animals to nose bleed.

R6: When the animals are bitten by the mosquitoes, they get flu, they have running nose and their bodies turn yellow which is a sign of Kala azar disease.

R3: The disease attacks the animals and you cannot eat its meat because it turns it color and it is not safe to be eaten.

R4: There is something I forgot and I would like to add, when animals are infested with ticks, the animals develop bones disease and sometimes hinder their movements.

*(Men discussing)*

R3: No, the ones that affects the bones is TB of the bones.

R1: Humans too get TB from animals when they drink milk from an infected animal.

R4: There is another skin disease which an animal gets from sleeping on human feaces.

**E: I would like you to tell us some of the symptoms of the RVF diseases that you are familiar with.**

**E: Before you answer that, please tell me some of the human diseases.**

*(Birds chirping)*

R4: Humans get infected by different diseases. When humans drink milk from animals without boiling it, they are prone to be infected by TB. Without getting treatment, it might infect even the bones and eventually humans die. Others seek medical attention and survive. The other one if affected by the RVF disease without treatment, they die too. Sometimes bleed to death. People get infected with typhoid, dysentery, cholera outbreaks if you don’t maintain cleanliness. People start to diarrhea and vomit and if they don’t seek early treatment, they can also die.

R2: People also get infected by brucellosis disease which is caused by drinking raw milk.

R8: These days, our people are really affected by cancer which was not there in the past.

*(Phone ringing)*

**E: R4 mentioned that people can also get infected by the RVF disease, right?**

Chorus: Yes

**E: How do you know that a person has been infected by the RVF disease? What are some of the symptoms that are seen in humans?**

R2: Their urines are yellow in color.

R6: They bleed from their mouth and nose.

R5: Their eyes become yellow.

R2: Their stomachs are bloated.

**E: R1 please tell us your experience because you told us earlier that you are a survivor.**

R1: I was infected but it didn’t make me weak. I experienced high fever only.

R3: If you are infected, you look weak and dehydrated.

R8: It’s just sometimes your day to die hasn’t reached, otherwise that disease hits you so hard that you just die. It doesn’t waste time.

R4: When you get infected by the disease, you lose your appetite for food, you become very weak that you cannot stand up. You develop pain in the joints.

R3: Before you even know that you are sick, you lose appetite, you have bloated stomach.

**E: What are some of the symptoms that are seen in animals?**

R1: The animals urinate blood.

Chorus: They have boated stomachs.

*(Birds chirping)*

R4: Fever.

R8: Abortion

R3: The infection is easily transmitted to other animals and humans.

*(Men whispering)*

*(Birds chirping)*

*(Men discussing)*

*(Birds chirping)*

**E: You mentioned that when an animal has the RVF disease, they urinate blood right?**

Chorus: Yes.

**E: Between the blood urine and stomach bloating, which show outright that an animal has been infected by the RVF disease?**

Chorus: Blood urine.

**E: How many are saying blood in the urine?**

**E: Between an animal that urinates blood and the one that has fever, which of the two is the strongest symptom of the RVF disease?**

Chorus: Blood urine.

**E: Between the animal that urinates blood and abortion which the symptom that shows that an animal has been infected?**

R4: When you see the first symptom that is all the symptoms come out.

R2: Blood in the urine comes later.

**E: What comes out as the first symptom?**

R2: Fever.

*(Men discussing)*

**E: Let’s understand each other. I am not asking about the sequence of the symptoms. I want to find out the difference in symptoms and that which can inform you that an animal has been infected by the RVF disease. So between an animal that urinates blood and the one that aborts, which one shows that an animal has been infected?**

Chorus: Blood urine.

*(Men discussing)*

**E: Between an animal that has a bloated stomach and fever which is the symptom that clearly shows that an animal has been infected?**

Chorus: Fever.

Chorus: Bloating

**E: How many are saying bloating?**

**E: Between the animal that has fever and the ones that abort, which symptom shows that an animal has been infected by the RVF disease?**

Chorus: Abortion.

*(Donkey braying)*

**E: Between fever and abortion, which clearly shows that an animal has been infected by the RVF disease?**

Chorus: Fever.

*(Birds chirping)*

**E: From our discussion, you have said that the main symptom that shows that an animal has been infected by the RVF disease are; blood urine followed by fever and then abortion and lastly bloating of the animal. Is that what you’ve said?**

Chorus: Yes.

*(Men talking in low tones)*

*(Birds chirping)*

**E: I would like us to discuss the effects of the disease on humans. You said that human’s urine is yellow in color, right?**

Chorus: Yes.

**E: So, between the yellow urine and the bleeding from the nose and mouth, which shows that a person has been infected by the RVF disease?**

Chorus: Bleeding from the nose and mouth.

**E: How many people are saying bleeding from nose and mouth?**

R4: These two symptoms are not far apart but the serious one is the bleeding.

**E: Between the yellow urine and yellowing of the eyes, which shows that a person has been infected by the RVF disease?**

Chorus: Yellow urine.

*(Birds chirping)*

**E: Between the yellowing of urine and bloating, which symptom clearly shows that a person has been infected?**

R5: Urine.

**E: How many are saying yellowing of the urine?**

**E: Between yellowing of the urine and fever, which symptom shows that a person has been infected by the RVF disease?**

Chorus: Urine

R7: Fever

**E: How many are saying fever? How many are saying urine?**

**E: Between Yellowish urine and weakness of the body, which shows the symptom of the RVF disease the most?**

Chorus: Yellowing of the urine.

**E: Between Yellowing of the urine and lack of appetite, which symptom shows clearly that a person has been infected by the RVF disease?**

Chorus: Urine.

**E: Between the yellowing of the urine and a person dying, which symptom shows that a person has been infected by the RVF disease?**

*(Birds chirping)*

Chorus: The urine.

**E: Between bleeding from the nose and mouth and yellowing of the eyes, which symptom strongly shows that a person has been infected by the RVF disease?**

R4: Bleeding.

**E: How many are saying bleeding?**

**E: Between bleeding from mouth and nose and bloating, which symptom shows that a person has been infected by the RVF disease?**

Chorus: Bleeding.

**E: Between bleeding from mouth and nose and fever, which symptom shows that a person has been infected by the RVF disease?**

R4: Bleeding

R8: Fever.

**E: How many are saying bleeding? How many are saying fever?**

R4: When you start bleeding even your death is near.

**E: Between bleeding and the one whose body is weak, which symptom shows that a person is infected by the RVF disease?**

Chorus: Bleeding.

**E: Between bleeding from mouth and nose and loss of appetite, which symptom shows that a person has been infected by the RVF disease?**

R4: Bleeding.

**E: By show of hands how many are saying bleeding?**

**E: Between bleeding from the mouth and nose and a person’s death, which symptom shows that a person has been infected by the RVF disease?**

R4: It is bleeding that shows. If you start bleeding, you are in a very critical stage.

**E: Between yellowing of the eyes and bloating, which symptom shows that a person has been infected by the RVF disease?**

Chorus: The yellowing of the eyes.

**E: By show of hands, how many are saying yellowing of the eyes?**

**E: Between yellowing of the eyes and fever, which symptom shows that a person has been infected by the RVF disease?**

Chorus: Yellow eyes.

**E: Between yellowing of the eyes and weakness of the body which symptom shows that a person has been infected by the RVF disease?**

Chorus: Yellowing of the eyes.

**E: Between yellowing of the eyes and loss of appetite, which symptom shows that a person has been infected by the RVF disease?**

Chorus: Yellowing of the eyes.

**E: Between yellowing of the eyes and a person dying, which symptom shows that a person has been infected by the RVF disease?**

Chorus: The yellowing of the eyes.

**E: Between bloating and fever, which symptom shows that a person has been infected by the RVF disease?**

Chorus: Bloating.

**E: Between bloating and weakness, which symptom shows that a person has been infected by the RVF disease?**

Chorus: Bloating.

**E: Between bloating and loss of appetite, which symptom shows that a person has been infected by the RVF disease?**

Chorus: Bloating.

**E: Between death and bloating, which symptom shows that a person has been infected by the RVF disease?**

Chorus: Bloating.

**E: Between fever and body weakness, which symptom shows that a person has been infected?**

Chorus: Fever.

**E: Between fever and loss of appetite, which symptom shows that a person has been infected by the RVF disease?**

Chorus: Fever.

**E: Between fever and death, which symptom shows that a person has been infected by the RVF disease?**

Chorus: Fever.

**E: How many are saying fever?**

*(Men speaking in low tones.)*

**E: Between loss of appetite and body weakness, which symptom shows that a person has been infected by the RVF disease?**

R1: Loss of appetite.

**E: How many are saying loss of appetite?**

**E: Between body weakness and death, which symptom shows that a person has been infected by the RVF disease?**

R4: Before a person dies, he becomes weak probably from not eating well.

Chorus: Body weakness.

*(Birds chirping)*

*(Men discussing)*

**E: Between loss of appetite and death, which shows that a person has been infected by the RVF disease.**

Chorus: Death.

R5: After you have stopped eating is when you die.

**E: we are not asking which precede the other but what symptom strongly shows that a person has been infected by the disease.**

*(Men discussing)*

Chorus: Death.

*(Men talking and laughing)*

**E: According to our discussion, the symptoms that shows that a person has been infected by the RVF disease are; Bleeding from the nose and mouth followed by Yellowing of the urine then yellowing of the eyes which is followed by bloating, fever, loss of appetite and lastly body weakness.**

*(Birds chirping)*

**E: To our second question and I would like you to tell me how both animals and humans get infected and how it is transmitted.**

R1: Humans get infections from the livestock and they also transmit to other humans.

**E: R5, how is the disease transmitted among human?**

R5: The animals are bitten by the mosquitoes, they fall sick

R2: They get infection from the vaccines.

**E: Which kind of vaccine?**

R2: The ones that are injected to the sick animal. We eat the meat and that’s how humans get infected.

R4: Humans get infection mainly from the animals and just by themselves. During the rainy season there is plenty of water and the mosquitoes breed and bit both humans and animals and they get infected. If you don’t sleep under a treated mosquito net, the mosquitoes will bite you and you might die from the infection. Sometimes the animals are bitten by the mosquitoes when they go to drink water from the swamp. Humans drink milk from the animal without knowing that they have already been infected.

R6: Sometimes this disease can be transmitted from the human’s feaces. Grass might grow on them and the animals will feed on the grass and thus get infected.

*(Phone ringing)*

**E: Is there any other way? Please put your phones on silent mode.**

R5: You asked us how humans get infected right?

**E: Yes.**

R5: When an animal is infected by being bitten by the mosquitoes, the herder also is at risk of getting infected because he spends most of his time with the animals.

R8: We thank God because this disease is not something that comes all the time. It comes about once in a while. It affects the animals the most because animals gaze mostly in the wilderness. They are bitten by the mosquitoes.

*Silence.*

**E: How do you prevent yourselves from getting infected by the RVF disease as community?**

R8: We have never sat as a community and decided on the ways of preventing ourselves but at the individual levels there are precautions, we take.

**E: Please tell us what you do individually.**

R3: I use haltheeth, hawacho, walthena

R4: Marasisa

*(Cock crowing)*

R3: When you use these herbal medicines, there is no way you will get infected by any disease. Sarba roba too

R2: When you normalize using these medications, your immunity level is too high even the mosquitoes die after biting you.

*(Cock crowing)*

**E: Is there any other way that you prevent yourself from being infected by the RVF disease?**

R6: Sleeping under mosquito nets.

**E: R8, is there any other way?**

R8: Boiling of milk before drinking, sleeping under nets and most importantly there was a method which is now not in use because of the generational change and people learning new things from seminars is the use of herbal medicine. These days’ people neglect the traditional herbs because the doctors told us that it is overdosed, and you cannot measure how much is needed to treat a certain disease.

*(Cock crowing)*

R5: It is true what R8 has said, we used to depend on our traditional herbs and people rarely fell sick. Our immunity levels were very high, and you could barely see people visiting the hospitals. Nowadays, people would rather die at the hospital gates that use the herbs. All this is brought by the seminars and trainings that our people have been exposed to. People lived long. You could hear people dying at very old age like 90 years but nowadays it is the young people who die and leave the old ones behind.

*(Birdchirping)*

**E: Is there any other way?**

*(Cock crowing)*

R8: The meat should be tested first before people eat.

**E: Do you help animals give birth when they are due?**

Corus: Yes

**E: Do you put on protective gear?**

R7: Nowadays a few people use gloves but of us use our bare hands like we used to in the past.

**E: Can one get infected from touching the blood?**

Chorus: Yes, you can.

**E: How do you protect yourself from getting infected?**

R5: We look for gloves first.

R3: If you don’t have access to gloves, you wash your hands well with soap after delivering the baby.

**E: What about vaccination are the livestock vaccinated against for example the RVF disease?**

*(Cock crowing)*

R4: It is the government which sends doctors to come and vaccinate our animals if there is an outbreak.

**E: Does the vaccine help the livestock?**

Chorus: Yes.

R4: When healthy livestock are vaccinated against certain disease, its immunity becomes strong and they won’t be affected if there is the outbreak of the disease.

**E: Of all the protective and preventive measures, which one do you think is efficient to prevent the infection of the RVF disease?**

**E: R3 mentioned that a healthy person might just use the herbal medicine to boost his immunity. R6 said that boiling milk will reduce the risk of infection. R8 said that the meat should be tested first.**

*(Cock crowing)*

**E: R4 said that we should put on protective gears like gloves when assisting the livestock when they are giving birth. R8 also said that vaccination helps an animal in boosting its immunity. When we put them all together, which method works the most?**

R8: Vaccination is number one.

**E: Why vaccination?**

R8: I have cows and not all of them might have been infected. So I will protect them from getting infected by vaccinating them against the disease.

**E: What do you think R6?**

R6: Vaccination is important because it prevents the future spread of the diseases.

**E: What follows vaccination?**

R8: Boiling of milk is very important. We really depend on milk it’s only that this time round drought has finished up our livestock.

**E: Why are you saying boiling of milk?**

R8: When you boil milk, the heat kills all the infectious disease that might have been in the milk.

**E: What follows the boiling of milk?**

R4: Testing of the meat because those are food and humans highly depend on them.

R5: What follows is wearing protective gears when helping the animals when they are giving birth. I will give priorities to the livestock because people depend on them. When they are infected, people are likely to get infected too.

R8: It is followed by sleeping under mosquito nets. The nets protect people from being bitten by sand fly and also mosquitoes.

**E: The last one is the use of herbal medicine. How does it help?**

R8: The herbal medicine are known medicines and are effective. The medicine is stronger than the hospital modern medicine. It would have been better if they were researched on and advice people on the quantities they can use and not overdose.

**E: In this session, we won’t talk much instead we will use cards to answer the question.**

*(Cock crowing)*

*(Men talking and laughing)*

**E: I will give each one of you three cards each.**

*(Donkey braying from a far)*

**E: There are two people. They are husband and wife. The husband’s name is Boru and the wife’s name is Amina. They own livestock like goats, sheep, cows and camel. In the year 2023, there was a pandemic in their region. There was a disease outbreak that affects both animals and humans. My question is how will Amina and Boru use their source of livelihood to deal with the disease outbreak? Does Amina have control over the livestock like she can sell part of it on her own without consulting Boru. This card is Amina, this other one is Boru and the last one is both Amina and Boru. When I ask a question, you choose one card and then you will tell us the reason of your choice. Is that clear?**

Chorus: Yes.

**E: The first question is does Amina have control over selling of the livestock?**

R8: I would like to ask you a question.

**E: Please do.**

R8: The animals belong to both of them, right?

**E: They are a married couple.**

R8: Ok there is something there but let’s continue.

**E: The livestock belongs to both of them. The question is does Amina have a right to sell the livestock? By using the cards, please show me if she has the right and if she doesn’t, the person with rights.**

**E: Amina 2, Boru 3**

**E: R7, please tell us why you chose Amina.**

R7: They got married and they had the livestock together. She takes care of them. She is an equal owner of the livestock just like Boru. So, she has the right to sell it.

**E: Is there any other reason?**

R7: Yes, there is. She is the mother of the household and she has given birth to his children.

**E: R4, why did you choose Amina?**

R4: She has the right.

R5: Please fear your Lord and say the truth we are all fasting. We boranas, we know each other.

**E: Please let’s listen to each other.**

R4: She has the right because she is his spouse. She might consult her husband before making any decision. Only a person who isn’t considerate will make decision on their own.

**E: You didn’t choose the right picture.**

R4: ooooh I got confused.

**E: R8, why did you choose Boru as the one who has the authority?**

R8: I chose Boru because he got married to Amina. When Amina came to his homestead, she didn’t carry with her anything. All the livestock belong to the husband. In our culture, the Borana culture, every decision ends with the husband.

**E: R5, without repeating what R8 has said, please tell us why you think Boru has the power over the livestock.**

R5: The livestock belongs to the husband as he is the head of the family. Amina doesn’t have any right over the livestock.

**E: R1, why did you choose Boru?**

R1: The livestock belongs to Boru as much as they are married.

**E: R6, why would they decide together?**

R6: He is the head of the household and she is the one who runs the house business. They have to sit down and consult each other and come up with solutions together. She cannot make the decision on her own.

**E: What will happen if she makes her own decision?**

R6: He won’t accept because he is the head of that household and she needs to consult him.

**E: What if she doesn’t consult him?**

R6: There will be conflict between them.

**E: How is the conflict resolved?**

R6: They sit together and talk it out.

**E: What happens if they don’t come into an understanding?**

R6: They will call an elder to sit between them.

**E: What happens if the elder doesn’t get to solve the issue?**

R6: The matter is then taken to the religious leader.

**E: What if they don’t get a solution?**

R6: A solution must be achieved. It doesn’t go beyond the religious leader.

R5: Sometimes the matter is taken to the Chieff Kadhi.

**E: R4, please don’t repeat what has already been said. Tell us why you chose both of them.**

R4: They make the decisions together because they work together in harmony. He is the head of the household; she runs the household business. They sit to together because they are married and the welfare of their family depends on the decision they make. They sit and consult each other before making any decision. She might have come with her animals which she was gifted by her father. She brings the livestock to her husband’s homestead. The animals are kept together with his animals. As much as the animals are hers, she cannot sell them without his permission.

**E: She cannot sell the livestock that she came with from her home?**

R4: She cannot sell because she is under her husband’s jurisdiction. She also belongs to him.

I also want to add that in terms of livestock in our culture, it is the husband that has the authority and power and the wife cannot do anything without consulting him but running the household chores that is her work. She can give out foodstuff without consulting him and he doesn’t have to interfere.

**E: R3, tell us why you said that they will do it together?**

R3: They are married, they are considered to be one. They will make decisions together. This makes them have a great bond.

*(Birds chirping)*

**E: R2, why do they have to decide together?**

R2: The livestock belong to both of them and they consult and come up with better solutions.

**E: Does Amina have any right to change the animals?**

R4: There is a way they a person might change the livestock. A person might buy cows and sometimes exchange a cow with another herder maybe because he wants to use the cow for other purpose

**E: Using the cards please show me if Amina has the authority to change the animals?**

*(Birds chirping)*

*(Men talking and laughing)*

**E: Five, Amina. Three Boru.**

**E: R1, why did you choose Boru and not Amina?**

R1: The livestock belongs to him and if he wishes to change it will be changed otherwise even if she wants to change and he refuses, the stand that he takes is the final.

**E: R5 say something.**

R5: The husband is the head of the household. He will be consulted on everything that needs to be consulted on. Everything happens as he wishes.

R8: The husband is like the president.

*(Phone ringing)*

R8: His words are final. He is above the law of his household. He can consult his wife before making decision or he can make it on his own.

**E: R7, Why would Amina have the right to change the animals.**

R7: When a woman gets married, she is given a cow for her bride price and also if she was given cows by her father, all the cows belong to her and no one can take it from her. If for any reason they separate or divorce with her husband, she can take her animals and leave. She can change what belongs to her but not her husbands.

**E: R6, does she have a right to change?**

R6: Yes she can. Since they are married, they are one. She cannot do anything out of ordinary. Maybe she wants to change the livestock and get better ones.

**E: R4, why do you think Amina has the right to change the animals?**

R4: She has the right to do anything. It is only in this generation where people change and behave like strangers sometimes. In our times, when you get married, you are one being and you trust each other. When she sees an animal that isn’t doing well, she can exchange it without being asked because there no ill motive behind it.

**E: R3, why does Amina have a right to change the livestock?**

R3: They are married and they have equal rights and when she makes the decision, it is for their own benefit. If the husband is not around and she sees that exchanging the livestock is a good idea, she can do it because there is trust between them and when he comes back, she briefs him on what she did.

R2: They are one and, in his absence, she can make all the decisions.

*(Birds chirping)*

**E: Does she have a right to go to the hospital like can she sell a livestock and take herself to the hospital? Show your answers using the cards.**

*(Men laughing)*

**E: R4, why does Amina have the right to go to the hospital?**

R4: These people live together in good times and when she is sick, there is no problem of her selling the animal and going to seek medical attention. Even when he is not around, she can sell it and tell him why she sold it.

R2: In most cases, they have planned already. May she was told not to die because of an issue and she can help herself or save herself using the livestock.

R7: She can ask him if he is around and if he isn’t she has the right to sell the animals and take herself to the hospital.

**E: R5, why doesn’t she have the right?**

R5: The husband is the head of the household and she cannot sell the animals without his knowledge. She takes instructions from him.

R4: When we haven’t understood the question, people will answer anything. Myself I have understood. You have asked if Amina is able to take the animals to take to the markets. There is no way that a woman can make her own decision without consulting her husband. Even if she wants to buy food, clothing or going to the hospital. If the husband is in jail, she has no right to sell the animals. She must consult his people that is his parents. That is how it works in Borana culture.

**E: R2, why would the two of them make decisions together?**

R2: They are considered to be one and if she falls sick, she can ask her husband. They consult and she can sell the animal. If he is not around but reachable on the phone, she will also call him and consult. And if he is unreachable, since there is trust between them, she can sell the animal and help herself.

R1: She cannot sell it by herself, she must consult him and maybe he has also seen that she is not feeling well. That is when he can sell the animal.

**E: They might have some money at home. Does Amina have the right to use the money and start her own business? Use the cards.**

(Men talking and laughing)

**E: Amina 3, Boru 3 and both 2.**

**E: Why does Boru have the right?**

R1: The money belongs to the husband and Amina cannot use the money without asking him.

*(Birds chirping)*

**E: R5, why does Boru have a right?**

R5: Boru has the right because he is the head of the household. For any decision to be made, he will have to give his consent.

**E: Can Boru use the money without consulting anyone?**

Chorus: Yes, he can.

**E: R8, what do you have to say?**

R8: Mine is short.

*(Men laughing)*

R8: In his household he is the president. What he hasn’t assented to cannot be done. What a woman can do without any consultation is the household chores like cooking, fetching firewood, washing clothes and buying food for the hungry children. Things like buying of clothes, matters to do with livestock and running of business is under his jurisdiction. This is because when he married her he promised to look after her welfare.

**E: R3, why did you choose Amina?**

R3: Since they are married and trust each other, Amina will not make decisions that will affect her family in a negative way because she will suffer the consequences too. She can make decisions if it is good for the wellbeing of the family.

**E: We are talking about starting a business.**

R3: Yes. The business is an asset and brings in profit. If she has nothing else to do, and there is money and the husband is away for other duties, I don’t see it as a problem for her to start the business as she will still make money.

**E: Please tell us R4 what you think.**

R4: She can because we live in different times. People have to trust each other. For example, there was this time I had traveled and I left my family back at home. Though I was far, my wife took a cow and sold it at Habasweina market. She got money and she used the money to buy foodstuff, and clothes and she remained with money to sustain the family for that period.

**E: Did she ask you before selling the animal?**

R4: No, she did not. When I came back, I applauded her for the good decision she made. When people understand each other, there will never be a conflict.

**E: R7, tell us why Amina has the right.**

R7: Amina will not misuse the money. She will use the resources to get more money. When she engages in business, she is looking for profit and there is no harm in increasing our wealth.

*(Birds chirping)*

**E: R2, why will they have to consult each other?**

R2: If there is money, they can consult each other and open a business where she will be in charge.

R6: The couple must have consulted each other and set aside the money, so if they want to start something they must start after consultation.

R8: What she asked was if Amina can take the money and start her own business.

R6: She cannot do it by herself. They must consult each other.

*(Men talking and laughing)*

**E: I have another story, there are two people. They are husband and wife. The husband’s name is Adan, and he is 45 years old. His wife’s name is Shariffa, and she is 40 years old. They have been married for three years now. They own livestock like cows, goats, sheep, and camel. They live in a place called Saleti. There is a certain disease that recurs every year for four years now. This disease affects both humans and animals. Shariffa has been invited to attend a seminar about the disease. We will be using the cards again to answer our questions. Can Shariffa make the decision of attending the seminar on her own?**

*(Men discussing and laughing)*

**E: 3 Shariffa, 5 Adan.**

**E: R7, why does Shariffa has the right to make her decision?**

R7: She has been invited for the seminar and there she will get more knowledge, so it is okay if she makes the decision on her own.

**E: R3, please tell us why Shariffa has the right to make her decision.**

R3: She is going to learn about what is happening in the world. If you interfere some form of conflict will arise.

**E: So, conflict will arise?**

R3: Yes. These days women are leaders, you find that there are female presidents and MPs so if you refuse her to go, there will be problems in that household. Since there is nothing bad that she is going to do.

**E: So, he fears conflict that is why he lets her go?**

R3: No, he doesn’t fear, He lets her go learn and bring the knowledge home.

*(Men laughing)*

**E: R4, why can she make her own decision?**

R4: They are a married couple. If people don’t have an understanding, they won’t get married in the first place. When she goes for the training, she gains knowledge which is good for both of them. The knowledge will help them tackle the disease if it happens to attack their animals. So, she can make her own decision of attending the seminar.

**E: R2, why did you choose Adan?**

R2: He is the head of the household, and she cannot go anywhere without his permission. It is not accepted by the Almighty. He cannot refuse her to go but it is a must that she tells him first before going anywhere. If he doesn’t want her to go, she won’t go.

**E: R5**

R5: Like we said earlier, he is the head of the household, there is no decision that can be made without his consent. If he allows her to go, she will go otherwise she will stay back.

**E: R6**

R6: Everything happens according to his liking. When they first got married, every responsibility was placed on him so that means she is under his care. She cannot just wake up and leave without his permission. It is not permissible even in our religion. Leave alone going to attend the seminar, even going to the next-door neighbor without his permission is not halaal.

**E: R8**

R8: You have asked us so many questions and all of that has an ending. I have a lot of views on this story. As an elder, I will try to highlight a few. The husband is the main decision maker in the household. A woman cannot make her own decision without involving the husband. If she does without his consent, then there is a misunderstanding between them. It is not in our religion or in our culture.

**E: So you mean to say that a woman cannot decide on anything?**

R8: She will abide by the decision made by her husband.

*(Men laughing)*

**E: Is there any other reason?**

R8: That is the main reason. Even the Christians exchange rings showing the commitments and same as Muslims you make commitments during Nikkah. The only thing she cannot ask permission about is doing her house chores.

**E: That’s it?**

R8: Yes. There is no progress in anything that the husband hasn’t assented to it.

**E: So if she attends the seminar and comes back more knowledgeable, will you see its importance?**

Chorus: There is no importance there.

R5: In fact there is a problem because she left without my permission. Her parents gave her to me and she is my responsibility should anything happens.

R4: Shariffa as a grown woman. She runs her household well. She takes care of the children and her husband too. When she goes out to learn and brings knowledge home, he should be happy. Times have changed. We live in the modern world. Everything has changed. We need to support each other.

R3: You just call each other over the phone and tell each other your plans.

R4: Yes. It is even easier nowadays.

**E: When she tells him, she has already made her decision.**

R4: Yes she has.

*(Men discussing)*
