# Supplementary material for: “Without a man’s decision, nothing works”: Building resilience to Rift Valley fever in pastoralist communities in Isiolo Kenya
Source: PLoS One. 2025 Jan 28;20(1):e0316015. doi: 10.1371/journal.pone.0316015 (PMC11774392; doi:10.1371/journal.pone.0316015)
Supplement: S1 Dataset — (ZIP) [file pone.0316015.s001.zip › Supporting Information Files/File 16.docx]

Enumerator: what type of livestock do you own? R2.

Respondent 2: goats.

Enumerator: goats R2. Somebody else.

Respondent 5: I have goats and I don’t have but the rest of the community has though most have died as a result of drought.

Enumerator: any other livestock that you have except of cows and goats.

Respondent: 1we have camels.

Respondent: 4 donkeys.

Enumerator: donkey. When you talk say your name. R6

Respondent 6: there is hen and donkeys.

*Child crying.*

Enumerator: R6, do women own livestock?

Respondent 6: yes, we have.

Enumerator: which livestock do you have?

Respondent: 6 all livestock in the compound.

Enumerator: which are those livestock.

respondent 6: sheep and goats. That’s husband and children.

Enumerator: that’s owned by husband. Does she own livestock?

Respondent: 7 unless something like her dowry she doesn’t own livestock.

Enumerator: does she own livestock?

Respondent: 6 she doesn’t unless she was given money to buy for herself.

Enumerator: who own donkey and hen?

Respondent: 5 donkey belong to a man; he uses to look for food with it.

Enumerator: why do women own hens?

Respondent: 4 hens are small poultry that are meant for a household,

Enumerator: what do men own?

Respondent: 3 mostly goats and sheep belongs to them.

Respondents all: cows also belong to them.

Enumerator: which disease mostly affects your livestock?

Respondent: 5 fever.

Respondent 2: fever.

Enumerator: without fever is their other disease?

Respondent: 5 they diarrhea

*Coughing.*

Enumerator: R5 what are you saying?

Respondent 5: they have fever and diarrhea, and they might even die.

Enumerator: is there another disease R6?

Respondent: 6 there is Rift Valley where you are not allowed to consume its meat until its examined.

Enumerator: has it affected this area?

Respondent: RVF affected us once.

Enumerator: R7 is there another disease?

Respondent: 7 gasdor.

Enumerator: raise your voice.

Respondent: 7 there is gasdor.

Enumerator: What are human diseases? R6.

Respondent 6: vomiting and diarrhea. Currently it affecting a lot of people.

Enumerator: R3, human diseases.

Respondent 3: there is fever, yellow fever which affects children and eyes turns to be yellow. Kalazar outbreak.

Enumerator: R4

Respondent 4: kalazar causes bloating in children and whitening of the body.

Enumerator: R6, Rift Valley Fever that you have mentioned does it affect human beings?

Respondent 6: yes.

Enumerator: it affects human beings.

Respondent 6: human beings are affected when they consume milk and meat from affected livestock.

Enumerator: what do you call Rift Valley Fever in local dialect in Borana?

Respondent: 7 Birele.

Enumerator: Birele is yellow fever.

Respondent: 2 *qando bifte.*

Respondent: 4 yes, that’s it. That’s how we call it.

Respondent: All yes, qando bifte.

Enumerator: R1, what are the signs of Rift Valley Fever?

Respondent 1: yellow urine, causes weakness

Enumerator: R2 what are the other signs?

Respondent 2: headache, diarrhea and high fever in the evening.

Enumerator: R4 other sign of Rift Valley Fever on human beings.

Respondent 4: headache, pain in the neck, lack of appetite.

Enumerator: any other sign R5?

Respondent 5: it causes dehydration and causes before someone realizes.

Enumerator: R5 is their other sign that it hasn’t been mentioned?

Respondent 5: there is, joint pains, stomachache and even causes diarrhea

Enumerator: R6

Respondent 6: vomiting.

Enumerator: vomiting.

What are the signs of Rift Valley Fever on livestock?

Respondent: 1 shivering, lack of appetite

Enumerator: another sign? When you see that sign you say its Rift Valley Fever.

Respondent: 3 meat changes color.

Enumerator: which color?

Respondent: 4 Yellow.

*Whispering and murmuring.*

Enumerator: other signs that you know with?

Respondent: 5 there is. When you slaughter the livestock, hearts and liver are different sizes, kidney changes the color *child crying loudly* *and phone ringing.*

Enumerator: this is manyatta Ganna?

Respondent: *chorus* yes, Ganna.

Respondent: 6 kidney is white in color such as ashes

Enumerator: any other sign?

Respondent: 2 the other issue is, there is tree called *biskuti mjinga, which* causes a lot of diseases.

Enumerator: the *frosti* one.

Respondent: 5 we looked at it and saw the disease is called by that *frosti*

Enumerator: the one you talking is that when livestock eat dies healthy? What are the other sign of Rift Valley Fever?

Respondent: 7 stillbirths.

Enumerator: is it once or mostly?

Respondent: *chorus responses* mostly, they have still births and they give birth to weak calves which dies later.

Enumerator: when they have still birth do placenta fall?

Respondent: 1 some fall some don’t.

Enumerator: on livestock Rift Valley Fever it weakens, yellow meat, fever, swollen liver*, phone ringing,* diarrhea which has blood spots, still birth with retained placenta. If livestock weakens and yellow meat which shows that livestock is affected with Rift Valley Fever?

Enumerator: this are the signs on human beings. You said yellow urine, weakness, joint pains. Now yellow urine and joint pains if we compare which shows Rift Valley Fever?

Enumerator: how do livestock and human beings contract Rift Valley Fever? R5.

Respondent 5: you have headache.

Enumerator: R6

Respondent 6: when you eat meat from affected livestock or even using the milk.

Enumerator: raise your voice.

Respondent 6: when eating meat from the affected livestock. If not cooking properly.

Enumerator: where do livestock get from? R4

Respondent 4: they get from kalazar. When livestock are bitten by kalazar their meat tastes bitter

Enumerator: R1 where do livestock get Rift Valley Fever from?

*Clearing throat.*

Respondent 1: livestock will be affected when bitten by kalazar

Enumerator: is there anywhere else that livestock gets from?

Respondent 5: when the animals feed on frosti.

Enumerator: Where did you learn or hear about the Rift Valley Fever?

Respondent: 2 Mosquito feeds on livestock, that’s what we know.

Respondent: 4 from the medical officers

Enumerator: I mean Rift Valley Fever R1.

Respondent:1 we don’t know about we just know *qando bifte.*

Enumerator: is it medical doctors or vet officers?

Respondent: 3 medical doctors.

Enumerator: through affected person or what?

Respondent: 5 through affected person.

Enumerator: anywhere else?

Respondent: 2 through vet officers where he tells the community not to eat meat and butcheries are also closed.

Enumerator: we will go to our fifth question. How do you treat someone affected by Rift Valley Fever?

Respondent: 1 we go to the hospital.

Enumerator: How do you treat Rift Valley Fever?

Respondent: 3 he is taken to hospital.

*Man interrupts the session.*

Enumerator: R6 am asking how you treat yourselves.

Respondent 6: taken to the hospital and hospitalized where the doctor will take of him.

Enumerator: is it a private or government hospital?

Respondent:6 we take them to the government hospital.

Enumerator: is there another treatment method for treating the affected person? Is there any way you use to treat people who have RVF traditionally?

Respondent: 7 there is.

Enumerator: R7 raise your voice.

Respondent: 7 we use herbal medicines like *bires, burquqe, waldhena, marasisa*. The herbals are diluted and drink it.

Enumerator: is there anyone who treats himself when he is affected?

Respondent:3 no there is none.

Respondent: 5 someone affected you take them to the hospital and get treated.

Enumerator: how do you prevent the spread of this disease?

Respondent: 2 if someone is found to be affected in a family, they should be examined.

Enumerator: any other way to prevent the disease? R5

Respondent 5: when it’s found to be on livestock, you take the livestock far away from the area and check if it has spread to other livestock.

Enumerator: any other way to prevent this disease?

Respondent: 6 use of nets.

Enumerator: putting nets. Any other way?

Respondent: 4 boiling milk

Enumerator: Any other way?

Respondent: yes.

Enumerator: R6

Respondent 6: cutting and clearing grass, preventing stagnant water in the area to prevent mosquitos.

Enumerator: Any other way?

Respondent: 7 use of treated mosquitos net from the hospital so that you can put in your house,

Enumerator: Any other way R4 can prevent the disease?

Respondent 4: boiling the meat and pouring the soup.

Enumerator: boiling the meat and pouring the soup. why are you pouring the soup?

Respondent: 4 the reason why we pour soup is, meat will have germs from affected livestock.

Enumerator: Does the meat have germs when you pour the soup?

Respondent: 2 You wash the meat thoroughly and pour the water you used, and you cook to kill the disease.

Enumerator: any other way?

Respondent 4: there is no other way.

Enumerator: R3 any other way?

Respondent: 3 we get protective gear mostly from the hospitals.

Enumerator: Any other way you prevent this disease except wearing hand gloves, boiling meat and pouring soup, moving the livestock to another area, putting nets, cutting grass, boiling milk. How do you prevent this disease from the livestock?

Respondent: 2 we prevent this by not eating meat from the livestock.

Enumerator: any other way? How do you prevent livestock?

Respondent: 1 vaccination.

Enumerator: vaccinating the livestock.

Respondent: 3 moving from watery places.

Enumerator: which is the most effective of all that you have mentioned? Which is number one? Boiling meat and milk, vaccinating livestock, which is the most effective?

Respondent: 5 vaccination.

Enumerator: how many say vaccination? Why vaccination?

Respondent: All it prevents the disease

Enumerator: don’t talk at the same time.

Respondent: 6 it prevents the diseases, during an outbreak it cures the disease.

Enumerator: who makes the decision to vaccinate your livestock?

Respondent: 7 the household head.

Enumerator: household head. Why the household head?

Respondent: 7 he is the owner of the livestock so anything concerning livestock he must be asked.

Enumerator: R3 why the household head?

Respondent 3: he is the head.

Enumerator: who takes it to vaccination?

Respondent: 3 the one who has the power.

Enumerator: they take it together.

Respondent: 2 when there is an outbreak you moved the livestock to a different place

Enumerator: moving the livestock is next. Have you agreed?

Enumerator: why move the livestock?

Respondent: 1 to prevent the spread of affected disease to your livestock.

Enumerator: who makes the decision to move the livestock?

Respondent: 4 The owner (husband)

Enumerator: why the owner?

Respondent: 4 he is the head he makes all the decisions.

Enumerator: who moves the livestock if the owner gives permission?

Respondent: 3 Man, he gives permission and moves the livestock because the wife will take care of the children.

Enumerator: what next? You said vaccination, moving the livestock, what next?

Respondent 6: cutting grass and draining the water points.

Enumerator: who makes the decision for cutting the grass and draining the water?

Respondent: 6 Husband. he makes the decision even if it’s paying someone to do the job.

Enumerator: if he makes the decision who cuts the grass?

Respondent: 2 if the wife has a chance, she cut it, if he has the chance, he cuts it. Even if it’s paying someone else to cut the grass.

Enumerator: what next? What’s remaining is boiling the milk, use of nets, cooking meat, use of gloves. What next?

Respondent: 3 cooking the meat and milk.

Enumerator: R3

Respondent: 3 cooking the meat and boiling the milk. you pour the first water and then add another, cook the meat and pour the water.

Enumerator: why is this next?

Respondent: 1 it helps in killing germ and virus.

Enumerator: who gives the decision on boiling?

Respondent: 1 the mother.

Enumerator: why her?

Respondent: 7 she takes care of the kitchen affairs.

Enumerator: what next? What’s remaining are nets and wearing hand gloves.

Respondent: 6 It prevents mosquitos,

Enumerator: who makes the decision for everyone to sleep under the net?

Respondent: 4 the father.

Enumerator: why the father?

Respondent: 4 he is the household head. He commands children to sleep under nets.

Enumerator: if he makes the decision who put the nets?

Respondent: 5 Mother.

Enumerator: R5 why the mother?

Respondent 5: to prevent mosquitos from biting the children.

Enumerator: the remaining one is wearing gloves. Why do you wear hand gloves when helping livestock in giving birth? R7

Respondent 7: your body might have a wound, so we wear it to prevent germs.

Enumerator: who makes the decision to wear gloves when helping livestock in giving birth?

Respondent: 6 Father.

Enumerator: why the father?

Respondent: 6 he is the one taking care of the livestock.

Enumerator: who helps the livestock in giving birth?

Respondent: 5 fathers

Enumerator: why father?

Respondent: 5 he is with the livestock most of the time.

Enumerator: who makes the decision when you want to sell or exchange the livestock?

Respondent: 3 Father.

Enumerator: why father?

Respondent: 3 The mother also makes the decision, but she has to inform him first.

Enumerator: but who makes the final decision?

Respondent: 2 The father,

Enumerator: why him?

Respondent: 2 he is the household head.

Enumerator: what about the hospital, when the wife wants to go to the hospital, taking children to the hospital, who makes the decision?

Respondent: 1 Father.

Enumerator: why the father?

Respondent: 1 he is the household head.

Enumerator: what about money, let’s say there is money, and the wife wants to use it, who will make the decision?

Respondent: 3 husbands.

Enumerator: R3 raise your voice.

Respondent 3: he makes the decision she just shares the idea that she want to use for something.

Enumerator: who makes the final decision?

Respondent: 2 fathers.

Enumerator: why father?

Respondent: 2 he is the household and surpassing him is against the culture.

Enumerator: I will narrate a shot story lets listen carefully. There are two people who are husband and wife, one is Boru and wife is Amina. They are pastoralist, they have cows, camels, sheep and goats. There was an outbreak of disease in their area. It affects human beings and livestock. Are we together?

Respondent: mmmh.

Enumerator: There are two people who are husband and wife, one is Boru and wife is Amina. They are pastoralist, they have cows, camels, sheep and goats. There was an outbreak of disease in their area. It affects human beings and livestock. My question is, we don’t talk, I will give cards and we use the cards to speak. This is Amina, we said she is Boru’s wife, this is Boru the husband, this is them. Does Amina have the power to sell their livestock? If she has the power, you hold this. If the husband has the power, you hold the husband this way, if they discuss together you hold this. Have we understood?

Respondent: mmmh.

Enumerator: now tell me does Amina have the power to sell the livestock? Don’t show me just hold this way. Just raise one card the one who has the power. If you want to raise the two you can. Everyone to show me the card. R8 show me yours. R6 show me. Everyone to show me. 1,2, 3, 4,5,6,7.

Scores

Amina-1

Boru-2

Joint-4

Reasons for Amina

Respondent 5: the reason why she has power is she has children, if the husband is not around she can take the livestock since she also owns the livestock. This is because they own the livestock together

Reasons for Boru

R2, why does the husband have the power?

Respondent 2: he is the one responsible for the livestock and household head.

Respondent 4: he is responsible for everything in the household.

Enumerator: R4 has said that he is responsible for everything in the household.

Respondent 7: he is the head he is in control of the livestock

Reasons for joint

Respondent 3: he is the head, and the wife cannot surpass his decision which may cause disagreement, so to avoid that she discusses it with the husband.

Respondent 6: he is the head, so they need to discuss

Respondent 8: livestock belongs to the husband, so he has control.

Enumerator: does Amina have, sometimes people exchange livestock, does she have the power to exchange the livestock?

Scores

Amina-1

Boru-1

Both-5

Reasons for Amina

Respondent 4: she has the power over livestock that belongs to her but not the one that belongs to her husband.

Reasons for both

Respondent: 7 They have children together and they will use to raise children, they will discuss and then exchange it to benefit the whole family

Respondent 8: everything in the homestead is under the husband, if she wants something she has to tell her husband and take it. It may cause disagreement if she decides on her own.

Enumerator: if there will be disagreement, how would they solve it?

Respondent: solving will take a long time, sometimes they even go to the elders.

Enumerator: who are these elders?

Respondent: parents from both sides can bring them together and solve the issue.

Enumerator: what would happen if they don’t agree?

Respondent: sometimes they even separate.

Enumerator: where would they go if the discussion is beyond them?

Respondent: there is Kadhi.

Enumerator: before khadi what is there?

Respondent: there is elders.

Enumerator: who are these elders?

Respondent: religious elders. If it’s beyond them they forward to kadhi.

Enumerator: R5 why would they discuss together? *Child crying*

Respondent 5: the reason they will discuss together, they own livestock together, but the decision is made by the household head

Enumerator: R8 why would they discuss together?

Respondent 8: they own livestock together so they will discuss it together.

Reasons for Boru

Respondent 1: the reason he has power is that they have children together, they are husband and wife. What the husband disagrees with, the wife cannot make a decision.

Enumerator: R3 why does he have the power

Respondent 3: he is the household head and there is no way the wife would surpass the husband in our culture.

Enumerator: when Amina wants to go hospital does she have the power to take the livestock and go to the hospital?

Scores

Amina-2

Boru-0

Both-5

Reasons for Amina

Respondent 4: Amina has the household responsibility, it’s the husband and wife. If the children are sick they can take them to hospital.

Respondent 5: the reason she is able is that she uses the livestock to take care of the children. When the husband is not around, she is just like the father, she can take that responsibility.

Reasons for both

Respondent: 6 The reason they discuss together is that they are parents to the children *murmuring and coughing.* That’s why they discuss it together.

Respondent 7: they will discuss together because the husband is the household head, if she becomes sick she won’t take livestock on her own she will tell him.

Enumerator: when she tells him, does he agree or disagree?

Respondent 5: he will agree because he is supposed to take care of her sickness.

Enumerator: Does Amina have the power to sell the livestock and venture into other business?

Scores

Amina-0

Boru-1

Both-6

Reasons for Amina

Respondent 5: she is the household wife, she will discuss with the husband she can do business, that’s why she has the power.

Reasons for both

Respondent 3: when she wants to do something, she comes up with an idea so that he agrees.

Respondent 6: money is not hers alone, he also has the shares, she cannot make decision alone, she must discuss with him.

Respondent 7: they discuss because it belongs to both of them.

Respondent 1: business can be used to educate her children, take them to hospital, she will discuss with the husband and do what he agrees.

Enumerator: does he disagrees?

Respondent 1: he doesn’t disagree; she can talk to him until he agrees.

Enumerator: I will narrate another short story lets listen. There are two people, the husband is called Adan and the wife is called Sharifah, Adan is 45 years old, Sharifah is 40 years old. They have been married for three years, how many years?

Respondent: Three years’ *chorus responses.*

Enumerator: they have been married for three years. They are pastoralists. They have sheep, goats, cow and camels. For the past four years, there is a disease which affects human beings and livestock, in that four year the disease keeps occurring. Sharifah was invited for a seminar so that she can tell them the disease in their area and also to gain knowledge. Does Sharifah have the power to attend the seminar? Or it’s the husband who has the power? Or they will discuss together? Show me the cards if the husband agrees or disagrees.

Respondent: this one?

Enumerator: you show mw with this card. This is Sharifah, this is Adan, and this is Adan and Sharifah. We are together? Does Sharifah have the power to attend the seminar? When will she discuss with him will he deny her? Who has the power?

Respondent: Sharifah will ask for a permission.

Enumerator: we use the card to speak.

Scores

Both-6

Sharifah-1

Adan-0

Reasons for both

Respondent 4: Sharifah doesn’t have the power to attend the seminar if she won’t ask for permission to attend the seminar.

Respondent:3 she will have to sit with the husband and agree

Respondent 7: all the responsibilities lie with the husband and for her to go she has to go with his authority and it’s not culture for her to go without his permission. She will explain that there is somewhere providing education about the disease and if the husband agree, she will go

Respondent 5: the reason she asks for permission is that he is the household head, if she goes without his permission it might bring problems.

Enumerator: if there is training do men deny women not to attend?

Respondent: 2 They are not denied they also attend but we don’t often have seminars here

Enumerator: when the seminar is in your area, are women allowed?

Respondent: 1 they are allowed but men must be in attendance.

Enumerator: Why are they denied if the seminar is far away?

Respondent 6: if the seminar is far away, a lot of things will have lost, since she is taking care of children. The husband cannot manage to do other things in the household. She might be pregnant and not able to go to far places, in such scenarios she is not allowed.

Enumerator: so, does this mean that she will not be allowed to attend seminars in far places?

Respondent: All, yes.

Enumerator: do you have knowledge about Rift Valley Fever?

*Coughing.*

Respondent 5: we know nothing about it we just heard about it.
