# Supplementary material for: “Without a man’s decision, nothing works”: Building resilience to Rift Valley fever in pastoralist communities in Isiolo Kenya
Source: PLoS One. 2025 Jan 28;20(1):e0316015. doi: 10.1371/journal.pone.0316015 (PMC11774392; doi:10.1371/journal.pone.0316015)
Supplement: S1 Dataset — (ZIP) [file pone.0316015.s001.zip › Supporting Information Files/File 2.docx]

Enumerator: my first question is, as community which type of livestock do you own or keep?

R6: donkey, cows, goats, camels, hens, and sheep

What else do you have?

R5: we have both sheep and goats.

All respondents: That’s all we own in the community

Enumerator: **who owns this livestock? Is it men, women, or children?**

Respondent 2: women own this livestock, and men also own livestock. But men own more than women. They all own livestock.

Respondent 4: if I might tell you, it’s God who brought both wife and husband together, such that the livestock which belongs to the husband belongs to the wife, and which belongs to the wife belongs to the husband. In cases where the wife becomes widowed, the livestock belongs to woman and children.

Respondent 3: according to the Borana culture cattle, sheep, goats, and camels belong to the man while hens and donkeys belong to the women.

Enumerator: **my question is, now you have said you have livestock, *which disease commonly affects the livestock*? (She reminds them to say their number when they talk).**

Respondent 6: the common disease that affects livestock, there is Gandhi, *hawars.*

Respondent 7: Silisa is there, such as a fever

Respondent 1: gasdori commonly in goats.

Respondent 2: the issue is the disease mostly affects the livestock during the drought period. There is hawars that make cows cough. The animals’ lack of strength has a problem which is caused by drought.

R 8: Silisa is caused by a hyena when it attacks livestock and its spreads when other livestock sniffs the infected livestock.

R5: There is an unknown insect that buzzes and kills livestock young livestock which is called kalazar. *(Murmuring)*

Respondent 8: we said that our livestock is cows, goats, sheep, camels, and hens. The diseases mentioned before are for goats and cows. The so-called hawars and silisa affects cows, while gasdor affects sheep and fever affects sheep and cows. Also, there is a disease that affects the sheep but not the goats are known as gasdor. There is a disease that does not affect the goats and is called hawars and silisa. The common cause of diseases in this area is ticks and kalazar which is even worse than the mosquito. The most disturbing thing that affects our livestock that is causing diseases are ticks and kalazar.

Enumerator: **is there any other disease that you know?**

Respondents 6: mostly in sheep their *sirgo*.

Respondent 7: marchakas in sheep.

Enumerator: **is there any other that you know?**

Respondent 7: diarrhea, where livestock shivers and dies.

Enumerator: **You have mentioned different diseases, have you ever heard about rift valley fever disease?**

**All respondents: yes**

Respondent 5: rift valley fever is caused by diarrhea, fever, and kalazar but remember this is for the goats. While the main disease in cows is sombesel is also known as hawars. Silisa is caused by hyena saliva after it attacks livestock and spread by smelling of attacked livestock.

Enumerator: **what are some of the signs and symptoms of RVF in livestock?**

Respondent 7: signs are lack of appetite, shivering, and lack of strength.

Respondent 8: at times the goats have blood in urine, running nose, and bleeding through the nose.

Respondent 2: whatever has been said, it’s the truth. What kills Borana livestock is kalazar. Kalazar is just a mosquito.

Respondent 2: I know. Livestock got fever and diarrhea. During drought goats dies at once.

Respondent 6: shivering. rift valley fever causes the goats to raise their ears and tails just like human beings.

Enumerator: is there anything you haven’t told me about Rift Valley Fever?

Respondent 3: stillbirth

Respondent 8: first, the most important the animal dies

Enumerator: **what is the most important thing you saw in a stillbirth?**

Respondent 4: during stillbirth, the placenta doesn’t fall.

Respondent 4: placenta doesn’t fall that fast.

Enumerator: *raise your voice.*

Respondent: placenta doesn’t fall.

Enumerator: ooh placenta doesn’t fall. Is there anybody who wants to say something?

Respondent 1: no, we are done.

Respondent 3: there is something else. We are pastoralists, and you are a veterinary, your research entails on the root cause and treatment of Rift Valley Fever.

Enumerator: Yes.

Respondent 5: we inherited and acquired this livestock. Since the colonial period, we had our ways of treating livestock such as injection and spraying pesticides. During the spraying of livestock, there are some who run away, and they don’t get enough treatment.

Enumerator: **my second question is, how do livestock acquire Rift Valley Fever? How is it transmitted?**

Enumerator: let’s answer one by one. You are number five.

Respondent 5: Livestock gets the disease from the environment through mosquitoes and kalazar bites which are both poisonous to livestock and human beings, but livestock don’t infect each other

Respondent 2: mzee has said everything. All insects that bite livestock leave something poisonous, so livestock gets a fever, diarrhea and dies. It doesn’t even graze.

Respondent 4: it spreads and is transmitted during the watering of livestock such that if livestock drinks from the same water point as infected livestock, they get the disease.

Respondent 6: when healthy goats drink water from the same water points as infected goats with gasdor it can easily spread to each other

Enumerator: **we are talking about Rift Valley Fever.**

Respondent 7: one day specialists visited us and told us not to eat meat from infected livestock. Humans can also get RVF through livestock. It can also be transmitted from livestock to human beings and from human beings to livestock through eating meat from infected animals.

Respondent 8: so, this disease can spread easily through livestock, especially at watering points. Rift Valley Fever can be spread when mosquito bites infected livestock and then bites healthy livestock, that’s how we see it.

**Enumerator: the ways how to identify RVF in animals are *“first stillbirth, followed by stillbirth, the third is diarrhea, and lastly death***

**Enumerator: number 7you said it also affects human beings, how does it affect human beings?**

Respondent 7: it affects human beings by bloating.

Respondent 3: human beings have a fever and shivers like livestock.

Respondent 4: someone died in Iresaboru where he bled through his mouth and nose.

R1: bleeding from body openings

R2: death

R8: fever

Respondent 5: One of my children died of RVF. At that time, we didn’t know about RVF. We went to seek for medication in Isiolo and Meru. He died and had buried him in Isiolo. Also, another son of mine contracted RVF, and we were able to contact doctors in Habaswein and they gave him medicine. So RVF is a very bad disease. In the same way, they built centers in Wajir, we are appealing to have the same center in Merti or Iresaboru.

Enumerator: **the first sign is bleeding *(nose/mouth*)** **the second is fever, this is bloating, fourth is death.**

Enumerator: **How did you learn about the rift valley fever?**

Respondent 3: learned it when our community was affected by RVF. At that time our animals were dying in large numbers and fever, and when you would slaughter the animal, we saw that the meat had turned yellow meat

Respondent 1: we saw the signs when our animals were affected by RVF in 2020 with massive abortion

Respondent 2: When it rains there is stagnant water where mosquitos breed, and it transmits it.

Enumerator: **you didn’t know about the rift valley fever, who told you?**

Respondent 4: from hospital reports when someone is infected with rift valley fever and taken to hospital. That’s how we knew.

Respondent 5: according to our culture it’s during the rainy season when there is stagnant water but during the drought period the disease is minimal.

Respondent 7: just as the old man said, when there is that disease, the disease surveillance team comes and takes samples.

Enumerator: Do they take samples?

Respondent 8: from the veterinary people

Respondent 6: when things get tuff they come and take samples and later bring results

Enumerator: question number 5, ***how do you treat the infected person?***

Respondent 7: if he/she is infected we take them to the hospital

Respondent 3: seeking medication

Five respondents said that when a family member gets infected with RVF are taken to the hospital

Respondent 1: another medication is using herbal medicine (*walthena*) when there were no hospitals/nowadays we take people to the hospital

Enumerator: so, you take people to the hospital, are there people using herbal medicine (*walthena*) up to date?

Respondent: (*chorus*) No

Respondent 5: there was no hospital, so our community use herbal trees which are many in our area

Enumerator: so, people have stopped using herbal trees?

Respondent 6: we go to hospitals

Enumerator: when you go to the hospital, is the private hospital or the government hospital?

Respondent 7: we go to the hospital

Enumerator: government hospital?

Respondent: (arguing and discussing) We go to a government hospital which tells you to buy medicine from the chemist

Enumerator: so according to your which hospital do you go to?

Respondent 1: I go to referral hospital (chorus and inaudible answers)

Enumerator: so, you go to government hospitals and not the private hospitals?

All Respondent: yes

Enumerator: Is anyone who does self-treatment and doesn’t go to hospitals and traditional healers? There are traditional healers?

Respondent 2: she is asking, anyone who does not go to either the hospital or traditional healers and self-treatment

Respondent 8: when someone is away from the area, and he has headaches and fever he just treats himself using the herbal trees

Enumerator: Are there any prevention measures you practice, apart from treatment?

Respondent 2: God protects us

Respondent 3: we use the mosquito net

Respondent 5: spraying of pesticides/insecticides like mosquito

Respondent 4: there is some medicine sold in shops you apply

Respondent 7: we send them to our pastoralists so that they can protect themselves too

Enumerator: Baba what are you saying?

Respondent 8: we just use that medicine to protect ourselves, especially during the rainy season

Respondent 6: another one we just wear long clothes

Enumerator: why do you wear long clothes?

Respondent 1: To protect ourselves from mosquito bites, we also cut long grass from our compound

Enumerator: anything else that you do? You said you can be infected through milk, what are doing with milk to prevent infection

Respondent 2: boiling

Enumerator: what else?

Respondent 3: avoiding eating infected meats

Respondent 5: other people say when you cook the meat eat the meat and don’t drink the soup (*laughing*)

Enumerator: how do you prevent it from spreading within livestock?

Respondent 6: injection, spraying pesticides

Enumerator: does any veterinary come to look at this livestock? When are you reporting that there are mosquitos, or you don’t even report there are mosquitos?

All Respondent: (*Murmuring*) By the time they are given a report and they act on the report the livestock are already dead (*all respondent agrees*) as we are doing the follow up the livestock are already died

Enumerator: Number 7 previously you said that when the animal gives stillbirth you can get the disease while touching the still one by bear hands, what can you do to prevent yourself from getting the disease?

Respondent 7: in such situations, people wear gloves and dispose it away or even use sticks to dispose of it away but not touching with their bare hands

Enumerator: how many are for the use of sticks to dispose of it without touching it with their bare hands?

Respondent: this is the norm for many people to dispose of the aborted fetus or other products since it’s the only possible means available.

Enumerator: raise your hands clearly (*counting*)

Respondent 3: Leave alone what they are saying it is normally disposed of by sticks

Enumerator: now, 6 people are that they don’t touch it with their hands, okay. Those of boiling milk how many people are saying that they usually boil milk?

Respondent 6: you have asked us to raise our hands if the milk should be boiled before consumption. It is not a common thing between us. This milk when we go herding livestock, we usually sat down milk the cattle, and drink it immediately

Respondent 8: yes, we don’t boil it. The milk which is being boiled is that milk when we come back from herding the women to use tomorrow for tea preparation, so they boil it to prevent it from being spoiled even this people don’t understand the difference

Enumerator: (murmuring) Now, among you, except number 7 all others don’t boil the milk, so, it is only one person who says he does boil the milk before consuming? okay about the veterinary you have said the veterinary usually delays.

All Respondents: the veterinary officers take a long to respond if there is an outbreak and their help is minimal

Respondent 1: sometimes they come during a drought during bedtime, when the livestock lack pasture, but they don’t come when there is pasture for livestock or an RVF outbreak

Respondent 4: they don’t help, because mostly the livestock have got malaria and there is no food it ends up dying

Respondent 8: If they come in time they would have help, but they don’t come in time

Respondent 5: and they are nothing to back up you to prevent this disease at time

Respondent 2: Mostly they come in during drought when you don’t have anything to feed on livestock

Enumerator: but if come in come they would have help?

Respondent: (all agrees) Yes, they will help especially that time when there is grass but they come when there is a drought when you have nothing for livestock, so the disease kills them.

Respondent 3: veterinary help but the reason why it doesn’t help it’s because the owner has seen his livestock have the disease, he reports since there is no vet here, the veterinary doctor is in Garbatulla, he/she takes time because he reports to Isiolo station so that’s why the vet has become helpless due to lack of time, otherwise they would have helped.

Enumerator: Now, we are at section B. I would like to tell you a story. In this story, I will ask a few questions and also you will tell me something from it, okay? Now, there is a person by the name Boru(*murmuring*), now the story I will tell you with these cards, you will answer it individually as you think, you will just show me the cards as am asking you, is it clear? Now let’s hold these cards in this way, in this manner. Let the side that is drawn face you, okay? Now we are starting our story, first, there is a person by the name of Boru, he has a wife known as Amina. They are husband and wife. These two owns livestock which are cows, camels, goats, and sheep. Is it okay up to there? They are married and they also own livestock. Now in 2023, there was an outbreak of disease in their area, there is what? The outbreak of the disease in their area affects both livestock and human beings, are we together up to there? the two own livestock, in 2023 there is an outbreak of disease that affects humans and livestock. Boru and Amina have done what to prevent themselves from this disease? So, we would want to know how Amina, boru and both use their ownership to respond to the disease.

**Can Amina make the decision to sell the livestock to avoid more lost**

**Scores**

Amina-0

Boru-6

Both-2

Reasons for boru

R3: He manages Amina and the children, and the household at large, so he makes final decisions on all that happens in the household.

R6: Everything in the household belong to the man. She was also just brought into the house through marriage, so he makes all the decisions about everything in the home

R1: the man is the household head and makes all the decisions. However, if he is not home, the lady can make the decisions.

R2: Amina can only have the power to make decisions about household resources when the man is dead but as long as the man is alive, she cannot make any decisions.

R5: Boru is in charge because for example, a head and the legs which one enters the house first. It’s the head and boru is the head and makes decisions for the legs which in this case is the woman

R 8: then, as the old man says, if she says something the husband buys it, are you listening? The reason he bought all this firstly, she just came and saw the livestock were already there, she did come with any of it (murmuring). Even if the livestock is equally owned by them, she cannot buy by herself

Reasons for both

R4: it is important to discuss and make a joint decision because Amina also contributes to caring for livestock production within household

R7: they decide both avoid conflicts within the household but also to appreciate the time women also put into livestock rearing.

Enumerator**: the second story is, there is one person known as Adan**. Adan’s wife is known as Sharifah. Adan is 45yrs old (background Sharifah) Sharifah is 40yrs old. They have been married for 3yrs. They are pastoralists they have cows, camels, goats, and sheep. Foe 4yrs their community has experienced both livestock and human disease. There is an organization that visited them and wanted to talk about this disease. Sharifah was invited for a seminar, to talk about the disease and also learn, are we together? Without informing Adan can she attend the seminar? Will Adan decide for her to attend the seminar? Will they decide together? How will their conversation be? Can she go with put informing her husband? Can her husband decide? Or decide together? You can rise what you think is right. If Shariffah can go without informing her husband you raise this, if Adan will decide to raise this, or if both will decide to raise this, are we together?

Enumerator: show me by your cards; don’t show each other Baba don’t look at his card

Scores

Shariffah-0

Both-8

Adan-0

Reasons for both

Respondent 1: there is no way she can go alone

Respondent 2: let them sit together but she can’t/ won’t go if her husband will not be alone with her. They are husband and wife, and the wife was invited to a seminar to talk about disease in her community and learn. She won’t go without her husband knowing. She must inform him. At times he can deny her and let them choose another person

Respondent 6: according to Islam Sharifah cannot move alone to far places without the company of her husband. So, they have to discuss so that the man can find time to escort her or find someone he trusts to accompany her.

R3: it’s important to discuss because the knowledge will be useful for the family at large in disease prevention

R4: the woman cannot go alone because she doesn’t have the power to make such decisions because the household role belongs to women. If she leaves for the seminar who will take care of the children? But the man can go anywhere.
