# Supplementary material for: “Without a man’s decision, nothing works”: Building resilience to Rift Valley fever in pastoralist communities in Isiolo Kenya
Source: PLoS One. 2025 Jan 28;20(1):e0316015. doi: 10.1371/journal.pone.0316015 (PMC11774392; doi:10.1371/journal.pone.0316015)
Supplement: S1 Dataset — (ZIP) [file pone.0316015.s001.zip › Supporting Information Files/File 3.docx]

**E: As community, which types of animals do you keep?**

R1: Cows, goats, donkeys.

R1: Cows, goats, and donkeys. The goats are both goats and sheep.

**E: Are there any other animals?**

R5: We mainly keep cows, goats, donkeys, and chickens.

**E: Is there any other animals other than the ones mentioned by R1 and R5?**

R3: Sorry I had stepped out. Have they mentioned chicken?

**E: Yes.**

**E: Among these animals, which ones belong to women and which ones belong to men?**

R3: As you know, do you have a house where you live in?

**E: yes.**

R3: Are you married?

**E: No.**

R3: From your observation on married people, do they have separate houses?

**E: No they have one.**

R3: So, for a husband and wife who live together, the animals belong to both of them.

**E: There are animals that are mainly known to belong to men and others mainly known to belong to women.**

R3: The one is clearly known to be hers is only one cow which she was given as her bride price and if there is any that she was given by her father.

**E: So that means that women too can own cows.**

R3: Yes they can.

**E: Is there any other animal that is known to be owned by women?**

R4: Donkeys are known to be owned by women.

**E: R2, is there any other animal that is known to belong to women?**

R2: Chicken

**E: What about men?**

R3: There are donkeys that belong to the man and the one that belong to women. Generally, it is the women that use the donkeys the most. She fetches water with it, she goes to fetch firewood with it.

R4: She uses the donkeys to help her with chores but it belongs to her husband.

**E: What about chicken?**

R3: She takes care of the chicken.

**E: Why can she own it?**

R3: because chickens are kept at home and she is the one who is always there and she feeds them.

**E: Why doesn’t a man own chicken?**

R5: What is known to be owned by men is cows and goats. And Chicken and donkeys are known to belong to women because they take care of them. Cows and goats are known to belong to men because they take them for grazing and water.

R6: In terms of taking care of the animals, the goats and cows belong to the men and chicken and donkeys belong to women.

**E: What are some of the diseases that affect both humans and livestock in this area?**

R3: Yellow fever

**E: The one people were vaccinated against?**

R3: Yes. Also there is another disease called *Salmonellosis* which we don’t know how to cure. Some of its symptoms are diarrhea, makes the cows lose their sights and dryness of the nose.

*(Birds chirping).*

R3: Also Foot and Mouth disease. We haven’t got the right medicine for it. We have lost many livestock to it. We are told that vaccination will be sent and we have seen none.

R5: Foot and Mouth disease and *Haemorrhagic Septicaemia.*

R7: Foot and Mouth disease. If the animals don’t get the right medication, they die from it. The cow’s mouth is filled with sores and they cannot eat. It breaks apart the hooves and they cannot walk.

R8: They have mentioned all the diseases that affect our animals.

R1: Sheep and goat pox is also another disease that affects our animals. It doesn’t have cure too. There is also CBPP.

R2: There is a disease which makes animals to urinate blood and die. We don’t know its name.

R3: Anthrax.

R4: CCPP

*(Men discussing)*

R3: There is another disease called *buth* it affects the bladder of an animal and the animal dies.

**E: What about the diseases that affect humans.**

R3: Yellow fever, Diarrhea, and vomiting.

*(Cock crowing)*

**E: R7, is there a disease that affects humans?**

R7: Yes. Fever.

R8: Cancer

R 2: rift valley fever

R3: Corona, rift valley fever

**E: Are there infections still?**

R3: Yes, the infection is there, it keeps on changing its symptoms.

*(Men talking and laughing)*

R: Kala azar and fever.

**E: Is there a difference between Yellow fever and the RVF disease?**

R3: There is no difference.

R5: They are the same only that they have different names.

**E: Have you heard of RVF disease?**

Chorus: It is different.

R5: Yellow fever is called *birrelle* and RVF is called *Qando Birte*.

**E: Are we in agreement?**

Chorus: Yes.

**E: How do you know if a person has been infected by the RVF disease? What are some of the symptoms?**

R5: Fever.

All, chorus: Yellowing of urine

R3: Headache.

R7: Chicken pox is also another disease that affects humans.

R3: We are discussing the symptoms of the RVF disease.

**E: R3, you were telling us about another symptom.**

R3: Yes, I am not a medical practitioner but I heard that a person has fever, diarrheas and nosebleeds.

R4: They diarrhea blood.

*(Men discussing)*

**E: What are some of the symptoms in humans?**

R2: When an animal is slaughtered, they find the meat has changed color and the liver is swollen. The meat is thrown away.

**E: Are there any other symptoms?**

*(Phone ringing)*

**E: Put your phones on silent mood.**

**E: R4, is there any other symptom that can be seen in animals?**

R4: No

R6: During the rainy season, there are mosquitoes which bit the animals and leave them infected by diseases.

R8: Shivering and it doesn’t eat grass.

R5: There is another disease that affects animals which is called *trypanosomiasis*.

R6: Diarrhea

R1: Teary eyes.

R5: The animals feels cold, it shivers and die.

R4: Loss of appetite.

R1: An animal dies.

R5: The animal aborts if it is infected.

R6: The placenta is retained.

*(Men discussing in low tones)*

*(Phone ringing)*

*(Men talking)*

*(Cock crowing)*

*(Someone coughing)*

**E: What I have written here are the symptoms in animals that you mentioned in our discussion. I would like to know between Abortion and retained placenta which symptom shows that an animal has been infected by the RVF disease?**

R5: Abortion.

**E: By show of hands how many are saying abortion?**

**E: Between abortion and fever, which symptom shows that an animal has been infected by the RVF disease?**

Chorus: Fever

**E: Between abortion and yellowing of the meat, which symptom shows that an animal has been infected by the RVF disease?**

Chorus: Yellowing of meat.

**E: Between abortion and swelling of the liver, which symptom shows that an animal has been infected by the RVF disease?**

Chorus: Swelling of the liver.

**E: Between Abortion and when an animal shivers from feeling cold, which symptom shows that an animal has been infected by the RVF disease?**

Chorus: Feeling cold and shivering.

**E: Between Abortion and diarrhea, which symptom shows that an animal has been infected by the RVF disease?**

Chorus: Diarrhea.

**E: By show of hands how many are saying diarrhea? Raise your hands properly, I want to count.**

**E: Between abortion and teary eyes, which symptom shows that an animal has been infected by the RVF disease?**

R5: Abortion.

**E: How many are saying abortion?**

**E: Between abortion and an animal dying, which symptom shows that an animal has been infected by the RVF disease?**

R4: Abortion.

**E: Between placenta retention and fever which one shows that an animal has been infected by the RVF disease?**

Chorus: The retained placenta.

**E: Between placenta retention and yellowing of meat, which symptom shows that an animal has been infected by the RVF disease?**

Chorus: Yellowing of the meat.

**E: Between placenta retention and swelling of the liver, which symptom shows that an animal has been infected by the RVF disease?**

Chorus: Placenta retention.

**E: Between placenta retention and shivering, which symptom shows that an animal has been infected by the RVF disease?**

Chorus: Shivering.

**E: How many are saying shivering?**

**E: Between placenta retention and diarrhea, which symptom shows that an animal has been infected by the RVF disease?**

R6: Retention of placenta.

**E: Between placenta retention and when an animal dies, which symptom shows that an animal has been infected by the RVF disease?**

Chorus: An animal that has died.

**E: Between fever and yellowing of meat, which symptom shows that an animal has been infected by the RVF disease?**

Chorus: Yellowing of meat

**E: Between fever and swelling of the liver, which symptom shows that an animal has been infected by the RVF disease?**

*(Someone coughing)*

Chorus: Swelling of the liver.

*(Donkey braying)*

**E: How many are saying Swelling of the liver?**

**E: Between fever and shivering, which symptom shows that an animal has been infected by the RVF disease?**

Chorus: Fever.

**E: Between fever and diarrhea, which symptom shows that an animal has been infected by the RVF disease?**

Chorus: Fever.

**E: Between fever and death of an animal, which symptom shows that it has been infected by the RVF disease?**

Chorus: Death.

**E: How many are saying death?**

**E: Between yellowing of meat and swollen liver, which symptom shows that an animal has been infected by the RVF disease?**

Chorus: The yellowing of the meat.

**E: Between yellowing of the meat and cold and shivering, which symptom shows that an animal has been infected by the RVF disease?**

Chorus: Yellowing of the meat.

**E: Between yellowing of the meat and diarrhea, which symptom shows that an animal has been infected?**

Chorus: Yellowing of the meat.

**E: Between yellowing of meat and an animal that has died, which symptom shows that an animal has been infected?**

Chorus: Yellowing of meat, the one that did.

**E: How many are saying the yellowing of the meat?**

R5: You will find the yellow meat on an animal that has died.

**E: Between a swollen liver and shivering, which symptom shows that an animal has been infected by the RVF disease?**

Chorus: The swollen liver.

**E: How many people are saying the swollen liver?**

**E: Between a swollen liver and diarrhea, which symptom shows that an animal has been infected by the RVF disease?**

Chorus: The swollen liver.

**E: Between a swollen liver and an animal that has died which shows that an animal has been infected by the RVF disease?**

Chorus: The animal that has died.

**E: Between an animal that shivers and an animal that diarrhea, which symptom shows that an animal has been infected by the RVF disease?**

Chorus: The one that shivers.

**E: How many are saying shivering?**

**E: Between an animal that shivers and the one that has died, which symptom shows that an animal has been infected by the RVF disease?**

Chorus: The one that has died.

**E: Between an animal that diarrheas and the one that has died, which symptom shows that an animal has been infected by the RVF disease?**

Chorus: The one that has died.

**E: According to our discussion we can say that the main symptom which shows that an animal has been infected by the RVF disease is when an animal has died followed by the yellow meat. Other symptoms are if the liver is swollen, fever, abortion and diarrhea.**

*(Phone ringing)*

**E: I would like us to discuss the human symptoms. Between fever and headache, which symptom shows that a person has been infected by the RVF disease?**

Chorus: Fever

**E: How many are saying fever, raise your hands.**

**E: Between fever and yellow urine, which symptom shows that a person has been infected by the RVF disease?**

Chorus: Yellow urine.

**E: Between fever and bloody diarrhea, which symptom shows that a person has been infected by the disease?**

Chorus: The bloody diarrhea.

**E: Between fever and nose bleeding, which symptom shows that a person has been infected by the disease?**

Chorus: Nose bleeding.

**E: Between fever and a person who has died, which symptom shows that a person has been infected by the RVF disease?**

Chorus: The death of a person.

**E: Between headache and yellow urine, which symptom shows that a person has been infected by the RVF disease?**

Chorus: Yellow urine.

**E: Between headache and bloody diarrhea, which symptom shows that a person has been infected by the RVF disease?**

Chorus: Diarrhea.

**E: Between headache and nose bleeding which symptom shows that a person has been infected by the RVF disease?**

Chorus: Nose bleeding.

**E: Between headache and a person who has died, which symptom shows that a person has been infected by the RVF disease?**

Chorus: A person who has died.

**E: Between yellow urine and bloody diarrhea, which symptom shows that a person has been infected by the RVF disease?**

Chorus: Yellow urine.

**E: Between yellow urine and nose bleeding, which symptom shows that a person has been infected by the RVF disease?**

Chorus: Nose bleeding.

**E: Between yellow urine and a person who has died, which symptom shows that a person has been infected by the RVF disease?**

Chorus: The person who has died.

**E: Between bloody diarrhea and nose bleeding, which symptom shows that a person has been infected by the RVF disease?**

R6; Nose bleeding

**E: How many are saying nose bleeding?**

**E: Between bloody diarrhea and death, which symptom shows that a person has been infected by the RVF disease?**

Chorus: Death.

**E: Between nose bleeding and death, which symptom shows that a person has been infected by the RVF disease?**

Chorus: Death.

**E: According to our discussion the main symptom of the RVF disease in humans is when a person dies followed by nose bleeding, yellow urine, bloody diarrhea, then fever and lastly headache.**

*(Men discussing)*

**E: Our next question is about how RVF disease is transmitted to both humans and livestock.**

R3: From being bitten by mosquitoes.

**E: Is there any other way?**

R5: Both humans and livestock get the infection from mosquitoes.

R3: Humans get infected from eating infected meat.

R8: You can get infected by drinking raw milk.

**E: How did you know about RVF disease? How did you learn to differentiate between its symptoms from the symptoms of other diseases?**

*(A child crying in the background)*

R5: When we slaughter the animals and find the meat yellow, it is said that it has been infected and we are not allowed to eat the meat. When we find the swollen liver, it is said that it has been infected by the disease. We also don’t eat meat.

R8: from fellow pastoralists

**E: What activities do you do in taking care of the livestock that can make you susceptible to getting infected by the RVF disease?**

(This question was not well understood by the respondents)

R6: When you drink milk from an animal that has aborted.

**E: What activities do you do in taking care of the animals?**

R4: You take the animals for grazing.

R8: Milking

**E: When you’ve taken the animals for grazing, can the herder get infected?**

R4: Yes he can.

R3: You cannot get infected from herding but from eating an infected animal’s meat and drinking its milk.

**E: Who slaughters the animals?**

Chorus: The herder.

**E: Is the herder male or female?**

Chorus: Male.

**E: Why men?**

R3: The animals are taken into the wilderness to graze and there, there are wild animals and men are brave and strong.

**E: What other activities?**

R3: Construction of cow sheds.

**E: When constructing the shed, can one get infected by the disease?**

Chorus: No he cannot.

**E: Are there people who use herbal medicine?**

R3: In the past there used to be herbalists who treated animals using fire. They burn rods and place the hot rods on the animal’s body.

R5: Nobody visits the herbalists these days.

**E: Are there people who self-prescribe themselves with medicines?**

R7: There is nothing like self-medication.

**E: How do you prevent the spread of the RVF in your community?**

R5: Cleaning the compound, burning bushes, and removing the dirt.

R3: Vaccination.

R8: By wearing gloves when helping an animal who is giving birth.

**E: Is there any other way?**

R1: After doing any kind of work, wash your hands. Keep short nails too.

R5: We avoid meat from infected animals and do not drink milk from an animal that has been aborted.

**E: Is there any other way?**

R7: You don’t drink milk from an animal that has just been vaccinated. You have to wait for a week.

R3: If you go to the hospital, you take the medicine as it has been prescribed for you.

**E: With all the measures you have mentioned, which one works best?**

R3: Vaccination

R6: Not eating meat and drinking milk from infected animals.

**E: Raise your hands if you think it is Vaccination.**

**E: Vaccination. Why vaccination?**

R3: It is said that prevention is better than cure, it is better to prevent yourself from any harm. Vaccination boosts your immunity.

**E: If there is a vaccination drive, who takes the animals to the vaccination center? Who gives the authority for the animals to be vaccinated?**

R3: The government brings the vaccine and the father gives the authority.

R1: It is the father who decides whether his animals are to be vaccinated or not.

**E: Why the father?**

R3: He is the owner of the livestock.

**E: Who takes the animals to the center?**

R1: The owners.

**E: Who are the owners?**

R5: The father, his children and also the herder.

**E: Why don’t women take the animals?**

R6: Women have a lot of work at home, they take care of the children and do the other house chores.

**E: What follows vaccination?**

R3: Instructions from the doctor. He tells us not to drink milk from a sick animal, not to eat meat from a sick animal.

*(Donkey braying)*

R5: Avoiding milk and meat from an infected animal.

**E: Between the doctor’s instruction and avoiding milk and meat from an infected animal, which is more effective? By show of hands, how many are saying avoiding milk and meat?**

**E: R5, why is it important to avoid milk and meat from an infected person?**

R5: You will get infected from eating the meat and drinking the milk.

**E: Who give orders that the milk and the meat from the infected animal should not be drunk and eaten?**

R3: It is the doctor’s instruction.

**E: I would like to know in the household who gives the instruction.**

R5: It is the father of the house.

R3: The father conveys the message from the doctor.

**E: Why does the father make the orders?**

R8: He is the head of the household.

**E: What other measure?**

R4: Cleaning of the environment.

**E: Why cleaning the environment?**

R4: For protection of germs and reduction of breeding places for the mosquitoes.

**E: Who gives the orders for the compound to be cleaned?**

R6: The father.

**E: Why him?**

R6: He is the head of the household.

**E: When the father gives orders, who does the cleaning?**

R1: The children.

**E: Why them?**

R1: They hurry in doing things.

**E: What other measure?**

R5: Washing of hands and use of gloves when helping an animal give birth.

**E: Why should a person do all that?**

R5: Washing hands prevent the spread of the disease. Wearing of gloves reduces the risk of transmission of the disease.

**E: Who gives the orders of wearing gloves and washing hands in the household?**

R5: The father of the house.

**E: Why?**

R5: He is the head of the household.

R3: All the instructions come from the doctor and it is the father that implements it in the household.

**E: Who uses the gloves and washes hands?**

R5: Everyone is supposed to wash their hands. Everyone who lives in that household. The gloves can be used by anyone who assists the animal to give birth.

**E: What happens when an animal has difficulty in giving birth?**

R5: You call the traditional healer if you don’t know what to do.

**E: He is the one who wears the gloves.**

R5: Yes and assists the animal in giving birth.

**E: Is the traditional healer male or female?**

R5: Male

**E: Why not women?**

R5: Women are fearful.

**E: Which measure follows that?**

R5: Doctors Instruction.

**E: Why the doctors instruction?**

R3: The doctor is knowledgeable and guides people in the right way. It is not a trial and error.

**E: Who gives order that the doctor’s instructions are to be followed?**

R3: The father

**E: Why him?**

R3: He is the president of that household.

*(A woman talking)*

**E: Who follows up on the doctor’s instruction?**

R4: The mother.

**E: Why the mother?**

R4: The mother is the one who spends more time at home.

**E: What follows that measure?**

R6: Cutting short of nails.

**E: Why do you cut your nails short?**

R6: They keep dirt under them.

**E: Where does the dirt comes from?**

R6: They keep dirt under them. When working in the field and taking care of the animals.

**E: Who gives instructions on cutting nails short?**

R8: It is taught by our religion.

**E: Who is supposed to cut their nails short?**

R8: Everybody in the household.

*(Men discussing)*

**Section B**

**E: I have a short story. Please listen and we will have a discussion after. There are two people. They are husband and wife. The husband’s name is Boru and his wife’s name is Amina. They own cows, goats, sheep and camel. In the year 2023, there was an outbreak of a disease which infects both animals and humans. We will use the cards that I have given you to answer the questions. How many cards do each one of you have?**

Chorus: Three

**E: This card is Amina, this other one is Boru and the last one is both of them. Are we clear?**

Chorus: Yes.
**E: How will they deal with spread of the disease? Is Amina able to take part of the animals and sell them?**

*(Birds chirping)*

**E: R5, why did you choose Amina?**

R5: Amina is the mother of his children and she has the right to sell the animals.

**E: Is there any other reason? R7 why doesn’t Amina not have the right?**

R7: He is the man of the house and she belongs to him.

**E: R6, why doesn’t Amina have the right?**

R6: He is the man of the house. The head of the house.

**E: What do you think R4?**

R4: He is head of the household and she is his responsibility.

R1: She cannot lead while he is present.

**E: What do you think R2?**

R2: She cannot make any decision by herself. He is the head of the house and he makes the final decision.

**E: R1, why can’t she make any decision?**

R1: He is the head of the household and what he says is what will happen.

R7: Women are considered children.

R6: Everything was planned on her wedding day. The husband took all her responsibilities and she is under him.

**E: R8, what do you say?**

R8: He is the head of the household.

**E: Does Amina have the power to change livestock? R7 why doesn’t she have the power?**

R7: Boru is the owner of the livestock.

**E: Why do they have to make the decision together?**

*(Someone greeting people)*

R6: He is the husband and she is the wife. For them not to have conflict in their households, they have to sit together and make decision together. That way they will make the best decision. If one of them makes the decision, there will be conflict in that household.

**E: R3, how do they resolve conflict in case it happens?**

R3: When conflict occurs, there is lack of peace in that house. You cannot eat or drink in peace. So the relatives from both sides come together, listen to both sides of the story and resolve the issue.

**E: If they are not able to resolve issues by themselves what happens?**

R3: Their parents are involved or even an elder in the village can resolve their issues.

**E: What if the parents are not able to resolve the issues?**

R3: They will be able to resolve it.

R5: They might fail to resolve the issues.

**E: R5, What happens if the parents fail to resolve the issues?**

Chorus: They go to the Kadhi’s court.

**E: Does Amina have the right to sell an animal and use the money to go to the hospital or take children to the hospital?**

*(Voices of women talking at the background)*

**E: R5, why does Amina have the right?**

R5: The livestock belongs to her as much as it belongs to him.

**E: R6,**

R6: The livestock belongs to both of them but she needs to consult him first.

**E: So if she makes the decision and inform him, there is no problem?**

R6: No problem.

**E: R8, why would they consult each other?**

*(Sound of children playing at the background)*

R8: Even in our Islamic teachings we are taught to always consult each other before making any decision.

**E: R2, why would they consult each other?**

R2: There is no way she can take the animals without consulting her husband and they should decide together what to do because the livestock belongs to both of them.

R3: She is not feeling well. She is not supposed to make any decision. It is the husband who is taking care of her. He will make the decision on which animal is to be sold and take her to the hospital.

**E: R4, why does Boru make the decision?**

R5: It is his responsibility to take her to the hospital so he should not consult her. He should do the due diligence.

**E: The couple might have some money kept in the house. Does Amina have the right to take the money and start her own business?**

**E: R6, why does Amina have the right?**

R6: She cannot the money and start the business without consulting her husband.

R4: If they have consulted each other, it is fine to start the business.

**E: R8**

R8: Just like I had mentioned earlier, every decision made in a household should have been consulted on.

*(Donkey braying)*

R2: She has to consult her husband. If he does not agree, she cannot continue with her plans.

R1: Every decision ends with her husband’s approval. If he is not around, she will have to call him and tell him about her idea.

**E: Will he accept?**

R1: Why not? These days women are perceived to be equal to men so he will let her do her thing.

R3: Your question was very clear. You asked as if Amina can take the money in the house and start her business, right?

Chorus: Yes.

R3: She cannot take the money, unless the husband gives her the money to start the business. Starting a business is risky. You can either make more money or make a loss. So, unless the husband approves, she cannot make any move.

**E: If she tells the husband, will he accept?**

R3: He should accept. Like for example you are educated, you will be given a chance to start your business.

R7: Without consulting him, you will do nothing.

**E: He makes the last decision.**

R7: Yes, he makes the last decision.

**E: I have another short story, there are two people who are husband and wife. The husband’s name is Adan, and he is 45 years old. The wife’s name is Shariffa and she is 40 years old. They have been married for three years. They own livestock which are cows, sheep, goats, and camels. There has been a dangerous disease which occurs every year and it infects both livestock and humans and it has been four years now. Shariffa has been invited to attend a seminar about the disease. Does Shariffa have the right to attend the training? Please use the cards.**

*(Sound of children playing.)*

**E: R8, why will they have to consult each other?**

R8: Shariffa cannot go out without her husband’s permission, she must ask permission from her husband as per the teachings of Islam. If the training is in the area she can go alone and if it is in a far place, he will have to accompany her.

**E: R5, why would they consult each other?**

R5: She will have to consult him because when she is at home, she has some responsibilities, so they will have to plan on who will take up her responsibilities while she will be away.

**E: R2, why would they have to consult each other?**

R2: She will have to put everything in order before leaving and she must consult her husband because he is in charge of the house.

**E: R7, why does the husband make the decision?**

R7: Our religion Islam teaches us that she has to seek her husband’s permission before going out.

**E: When she tells him, will he grant her permission?**

R7: Yes he will.

**E: What if the seminar is held at a different town?**

R7: They will go together.

R6: He is the head of the family; his wife is his responsibility and also that is how Islamic Sharia teaches us that.

R4: If he hasn’t given her permission there is nowhere, she can go.

R3: In Islam, a woman cannot go anywhere without asking permission. When she goes, her husband might start to get jealous and bring mistrust between them. If she goes far, she must be accompanied. She is his responsibility and she is under his care.

**E: Can he not allow her to go?**

R3: Yes, he can if the place is unknown or the intentions are not very clear.

*(Men discussing and laughing)*

**E: If she has to go to a far place, will he leave all his responsibilities and take her there?**

R4: Yes, he will leave everything and go.

All respondents: She will have to stay home.

*(Men discussing)*

R5: Women are like ready food and they are wanted by everyone. You fear to lose yours so you have to take her where she wants to go.
