# Supplementary material for: “Without a man’s decision, nothing works”: Building resilience to Rift Valley fever in pastoralist communities in Isiolo Kenya
Source: PLoS One. 2025 Jan 28;20(1):e0316015. doi: 10.1371/journal.pone.0316015 (PMC11774392; doi:10.1371/journal.pone.0316015)
Supplement: S1 Dataset — (ZIP) [file pone.0316015.s001.zip › Supporting Information Files/File 5.docx]

(Sound of motorcycle passing)

**E: As community, which types of animals do you keep?**

R5: Goat, Cows

R4: Cows, goats and donkeys.

R6: Chicken

*(Someone greeting people)*

**E: R1, do women own livestock?**

R1: They are the ones who own livestock these days.

*(Men talking)*

**E: R7, to put you up to speed, we asked the types of animals that are found in this community and we have been told that there are cows, goats, donkey and chicken. Is there any other animal that has not been mentioned?**

R7: No

**E: Coming back to you R1, do women own livestock?**

R1: There are some that have.

**E: Which animal do they own majorly?**

R1: Goats. They have a few which are kept at home.

**E: Where do they get the goats from?**

R1: They are given by relatives and sometimes buy.

**E: R2, Are there animals that are known to belong to women?**

R2: They can get the animals from their parents.

**E: What kind of animal is she given by her parents?**

R2: Cows and goats. Also when they get married, they can be given by their husband as bride price.

**E: R4, are there other animals other than cows and goats that are known to be owned by women?**

R4: Everything that is owned by men is in their hands. The men give everything to the women to manage.

R5: Chicken belong to the women. Men don’t interfere with the chicken.

*(Birds chirping)*

R6: Women keep dairy goats so that they can milk for home use.

E: Why do chicken belong to women?

R3: It is because chicken can feed around the compound.

**E: To whom do donkeys belong?**

R1: It belongs to both men and women.

Chorus: Donkeys belong to women.

R3: She uses the donkey to fetch water and firewood.

R5: When her daughter gets married she asks the groom’s family to give her a donkey. She says that the work her daughter used to do in the household will be done by the donkey.

**E: I would like you to tell me some of the diseases which are common in your community that affect both humans and animals.**

*(Birds chirping)*

R7: Kala azar comes from insects that mainly breed in the cow dung. There is also the RVF disease which is transmitted by mosquitoes mainly during the rainy season.

**E: Is there any other disease, R5?**

R5: CCPP

R4: Sickness in animals limits its growth. The main problem we have is fever. When an animal is slaughtered, you find that the meat has turned yellow.

**E: The fever that you are talking about, is it RVF?**

R4: yes it is that one.

**E: R3, is there any other disease?**

R3: I herd cows and we have a huge problem. Without enough knowledge, we just assume that an animal is infected by the RVF disease without being sure about it. There are people like veterinarians who are supposed to come by, check the animals and tell us the real problem and the real disease. We just buy vaccines and inject the animals.

**E: Is there a disease that you know?**

R3: Cows are sick even now and I cannot really tell what they are suffering from.

**E: R7**

R7: We don’t get services from the veterinary. When an animal falls sick we use the trial and error method to cure the animal.

**E: R4,**

R4: There is a part in the stomach of an animal which is supposed to clean the stomach but it doesn’t. When an animal is slaughtered, that thing looks like stone.

**E: Is it in the intestines?**

E4: No, it is in the stomach. Also animals don’t want to drink water and the water around them. We assume the disease comes from eating the fruits of the mathenge tree. These days the donkeys are also affected. No one among us has any knowledge on the diseases that affect the animals.

**E: What are some of the diseases that affect humans?**

R5: Fever.

R4: Cancer, these days people get it like it is flu.

*(Phone ringing)*

R3: We are living in fear these days. We don’t know if there a hidden agenda by the white people to reduce our population because our children are being vaccinated too many time and it is worrying. We have so many questions and we don’t know whom to ask. I watched a certain video and shows that all the children who have been vaccinated will not be able to bear children.

R3: Children are sent to their parents to seek consent for them to be vaccinated. If the parents refuse to give consent the children are not vaccinated. That is just a by the way. The main disease which is very common these days is cancer.

R4: When a woman gives birth to the first child, she might stay for a very long time before getting another.

R3: That one too is a disease

R4: Women have issues with the whole of their reproductive system. They always complain of infection and back pain. There is no infection it is the family planning injection that causes the pain.

*(Motorbike passing)*

R4: Instead of their monthly menses coming out, it stays in her uterus. Then they start complaining about headaches.

R5: Like what R4 said, the infection affects even older women.

*(Men laughing)*

**E: What are the signs and symptoms of RVF in humans?**

R6: Before we go on, there was a disease that we forgot. It is diarrhea and vomiting. It is a combination of typhoid and dysentery. I became ill and when I went to the hospital, I was diagnosed with typhoid.

**E: What are some of the symptoms of the RVF disease in humans?**

*(Children playing at the background)*

R4: The RVF was first detected in 2007. that time both human and livestock lost their lives. One of the symptoms is vomiting blood. A person bleeds from mouth and nose.

R8: Diarrhoea.

Chorus: Yellowish urine.

**E: Please lets speak one at a time. Is there any other symptom.**

R4: Swelling of the body.

**E: What are some of the signs and symptoms of RVF in animals?**

R4: Bloating then it starts to diarrhea.

*(Children playing in the background)*

R7: Urinates blood. When slaughtered, the meat is yellow.

R1: Yellow urine and fever.

R3: It shivers because of a fever.

R4: Yellowing of the eyes.

R7: Animals die in large numbers.

*(Phones ringing).*

R4: The animals transmit the disease to the other animals.

R3: Massive abortion

**E: How do humans and animals get infected by the RVF disease?**

R4: They are bitten by mosquitoes.

R2: Sandfly

**E: How do humans get infected?**

R6: From drinking milk and eating meat from an infected animal.

R3: The disease is transmitted from the animals to humans when they ear meat or drink milk from an infected animal.

**E: How did you learn about RVF disease?**

*(Birds chirping)*

R3: The eyes of a person turn yellow and their urine too turns yellow.

**E: How did you know that the disease was the RVF?**

*(Children playing at the background)*

R4: Before going to the hospital, we try to figure out the symptoms, bloating of the stomach, loss of appetite and yellowing of the eyes and urine and that is how we know that it is the RVF disease.

*(Motorcycle passing)*

**E: You learn from the symptom. Do the doctor tell you that you have been infected by the RVF disease?**

R2: The doctor doesn’t tell us anything.

R5: If you go to the herbalist, he will give you medicine even though he is not very sure of what one is suffering from.

**E: What are some of the activities you do when taking care of the livestock that can make you at risk of getting infected by the RVF disease?**

R7: When milking the animal and drinking the milk or eating the meat from an animal that has been infected.

**E: Who milks the animal?**

R7: It is us or our wives.

*(Birds chirping)*

**E: Why your wives?**

R7: It has always been like that in our culture. Yes, she takes care of everything in the household. The milk is used in the household, so she is in charge.

**E: You also mentioned that you eat meat. Who slaughters the animal?**

R5: The men in the household.

**E: Why them?**

R5: It is our culture, and our religion Islam also teaches us that.

*(Children playing in the background)*

R4: Women are fearful, even you for example will not accept to slaughter an animal.

**E: Women fear.**

R4: Yes, they even run away.

R1: They wait for the meat to be brought and they cook the meat.

*(Children playing at the background)*

R2: After slaughtering and skinning the animal and cutting the meat, a person might get infected by the disease.

**E: Who does the cutting and the skinning?**

R2: Majorly men.

**E: Is there any other way a person might get infected?**

R3: You only get infected when you eat meat or drink milk from an infected animal.

R2: When someone urinates in the cowshed where the animals sleep, that person might get infected.

**E: Who urinates in the cowshed?**

R2: The men.

*(Men discussing and laughing)*

**E: Can you get infected while herding the animals?**

Chorus: No.

**E: Who takes care of an animal or a person who has been infected by the RVF disease?**

R4: The father.

**E: Why him?**

R4: Because he is strong, and he can vaccinate the animals and give medicine to the animal.

**E: What about a person who has been infected? Who takes care of him or her?**

R8: It is the mother who takes off the person, but the father is there for support.

**E: When does the RVF disease occur the most?**

R7: During the rainy season.

**E: Why the rainy season?**

R2: There are a lot of mosquitoes that transmit the disease during this period.

*(Phone ringing)*

R1: There are a lot of insects that bite animals during the rainy season

**E: Other than going to the hospital, what else can you reduce the spread of the RVF disease?**

R4: Use of mosquito nets.

R5: Clearing the bushes and cleaning the compound.

R1: Smoking the house to prevent the mosquitoes from entering.

R6: Use of mosquito coil.

*(Children playing at the background)*

R7: The mother shuts the doors and windows early in the evening.

*(Birds chirping)*

R3: Covering all the water storage containers.

R1: Draining of stagnant waters that reduces the breeding of the mosquitoes in that water.

**E: How do you protect the animals?**

R3: By moving the animals away from the water bodies like now the river is flowing.

R5: Burn wood and use the smoke to scare away the mosquitoes.

R7: Spraying the animals with pesticide.

**E: Are vaccination perceived as an option?**

Chorus: There is none that we know off.

**E: There is no RVF disease vaccine?**

Respondent: All, we don’t know of any RVF vaccine.

*(Men talking and discussing)*

**E: can helping animals with bare hands exposure them to risk of contracting the RVF disease?**

R5: You cannot.

*(Children playing at the background).*

**E: You earlier said that when an animal is infected, it is the father who takes care of it, right?**

Chorus; Yes.

**E: As the father takes care of the sick animal, isn’t he at risk of contracting the RVF disease?**

R4: He can get infected if he drinks the milk or eat the meat from the animal.

**E: How can he prevent himself from not getting infected?**

R4: By boiling the milk.

*(Sound of a child talking)*

R6: We don’t know if it is true because we are not educated but we have heard that if the meat is well cooked and the milk is boiled well, the heat kills all the germs and it is safe for consumption.

**E: Is there any other way?**

Chorus: There is none.

**E: From all the measures taken to prevent yourselves from getting infected, which is the most effective?**

R7: Use of mosquito nets.

R4: Draining of the stagnant water and clearing the bushes.

**E: Why do you think net is the most effective?**

R7: The mosquitoes are in plenty at night. We have women and children of the household and they need to be protected. In the evening the nets are draped down and children and women sleep in the nets.

*(Men talking )*

**E: Why net R4?**

R4: Net is very important in protecting people against being bitten by the mosquitoes. If it is draped early in the evening and doors and windows are shut early, net stands out as the best measure in prevention of the RVF disease.

**E: Who authorizes the nets to be draped?**

Chorus: The mother.

**E: Why her?**

Chorus: She manages all the chores in the house.

**E: What is the other most effective measure?**

R7: Draining of stagnant water and clearing of the bushes.

**E: Why?**

R7: In the past, we never used to have nets. Nets were introduced to us in the recent past. In the past, we used to hide ourselves from being bitten by the mosquitoes. We would make long (in height) beds. The mosquitoes couldn’t come up. They would bite you in the evening. Clearing the bushes and draining the stagnant water reduces the breeding of the mosquitoes in those places.

**E: Who gives the authority of the clearance of the bushes and draining of the stagnant water?**

Chorus: It is the mother.

R5: The draining of water, it is the mother that gives the authority and clearing of the bushes it is the father.

**E: Why the difference?**

R7: The father clears the bushes because the mother already has a lot of house chores to look after.

**E: Which measure follows the clearing of the bushes/**

R4: Cooking meat and boiling of milk.

**E: Why?**

R4: When you cook meat and boil milk, the heat destroys the germs and bacteria.

**E: Who gives the authority for the meat to be cooked and the milk to be boiled?**

R3: It is the mother who gives the authority and her who cooks the meat and boils the milk.

**E: Why her?**

R4: Cooking is part of her chores.

**E: Which measure follows?**

R6: Closing of windows and doors in the evening.

**E: Why is it effective?**

R6: It reduces the rate at which the mosquitoes enter into the house.

(Birds chirping)

**E: Who authorizes that door and the windows to be closed?**

Chorus: The mother.

**E: why her.**

R6: She runs all the household affairs.

R7: The house and the compound are two separate places. The whole compound and whatever is inside, belongs to the husband and the house belongs to the woman.

**E: She gives the authority, who does that work?**

Chorus: Her children or she does it by herself.

**E: Which measure follows that?**

Chorus: Mosquito coil.

**E: Why mosquito coil?**

R4: It is place in the house. The mosquitoes don’t enter the house if they smell the smoke.

**E: Who gives the authority to burn the mosquito coil in the house and who burns it?**

R4: It is the mother.

**E: She does both?**

R4: Yes.

**E: The measure is spraying the animals. Why are animals sprayed?**

Respondent:2 To prevent the mosquitoes and other pests from biting the livestock.

**E: Who gives the authority for the animals to be sprayed?**

Respondent: All The father.

**E: Who sprays the animals?**

Respondents: All The father.

R1: They older children help in spraying the animals.

**E: Are the children male or female?**

Chorus: Men.

**E: Who takes care of the welfare of the livestock. Who is the most responsible person?**

Chorus: The father.

**E: Why him?**

R2: He is the owner of the livestock and the head of the family.

R3: He is the manager.

**E: Who makes the decision of going to the hospital or taking children to the hospital to seek medical services?**

R5: The woman cannot go on her own. She has to be given money by her husband.

**E: Why the father?**

R5: They are his responsibility.

**E: Who has the authority over how the income is used in the household?**

R7: Whatever money we have is given to the mother. She is the treasurer.

**E: Does she have the right to use the money that she has been given?**

Chorus: Yes, she has all the right.

R5: She knows how to spend it. If she asks for more money is when we question if we feel like it has not been well utilized.

*(Men laughing)*

**E: I have a short story for you and we will answer a few questions later.**

R1: I hope they are not many, we don’t have a lot of time.

**E: We will summarize.**

R2: Prayer time has already passed. May the Almighty forgive us. You are our guest and guests are supposed to accommodate.

**E: I will give you three cards and you will use the cards to answer the questions after the short story.**

**E: There are two people. A man and his wife. The man’s name is Boru, and the woman is Amina. They own cows, goats, sheep, and camel. There was a disease outbreak in their area. The disease affects both humans and livestock. My first question is, how will they use the resources they have to protect themselves from getting infected by the disease? Does Amina have the power to sell the animals?**

R3: So we will use the cards to answer?

**E: Yes. This card is Boru, this other one is Amina and this one is both of them.**

*(Birds chirping)*

**E: The first question is, does Amina have the right to sell the animals? Who as the right or do they have to consult each other? Use the cards.**

**Scores**

Amina-0

Boru-4

Both-3

**Reasons for both**

R2: They have to consult each other because they live together and they want the best for the family. When they decide on the animal that is to be sold, the husband takes the animal to the market and bring her the money.

R8: They have to consult because they are the ones responsible for their families. They will have to agree on the decisions they make.

**E: What if one of them makes the decision?**

R8: There will be a disagreement between them.

**E: How can the disagreement be resolved?**

R8: By both of them sitting together and talking it out.

**E: What if they are not able to come to an agreement.**

R8: They call an elder.

**E: Is the elder a relative, or village elder?**

R8: The tribe elder.

**E: Can the issue get past the elder?**

Chorus: No, it cannot.

**E: Why will they consult each other?**

R4: Without consulting each other, no one has the right to sell the animals.

**Reasons for Boru**

R1: Because the livestock belongs to him, and he is the most responsible person for the family.

R5: He is the head of the house. He is responsible for all their needs. He might consider consultation, but he makes the last and the best decision for the family because they are his responsibility.

R6: Because he is the head of the household. In our culture, women are children and we can consult them sometimes, but the decision is made solely by the husband.

R7: When she was married, the husband took all her responsibilities. It was never said to share the responsibilities. We don’t really consult the women; we pass the information about the decision that we have already made.

**E: Does Amina have the right to change the animals?**

**Scores**

Amina-0

Boru-7

Both-0

Reasons for Boru

R1: Maybe he finds a better animal

**E: Why can’t she do it?**

R1: She can change too. If he is not there, she can change but if he is around, she will have to tell him about it. The decision is still his.

**E: R2, tell us why he is the decision maker.**

R2: He goes out to look for income or if it is livestock, he is the one to look for them. Whatever he brings from outside it is given to the mother to be managed by her.

**E: R3, why him?**

R3: There is a Borana saying which says that “A woman knows what’s inside the house while a man knows what’s inside the compound” that means that if there is an animal that needs to be changed, it is the father that knows, and she doesn’t know the difference.

**E: R8**,

R8: The father is the one who makes the decision. The woman makes all the decisions inside her house.

R4: I have the same opinion.

R5: They said it all.

*(Birds chirping)*

R7: Exchanging the animals might bring problems because the one she is exchanging with might not be the rightful owner. If a problem erupts, she is not going to be called by the elders, it will be my problem. The cow that she might exchange with might be a stolen one and I will be the one who will be summoned. To avoid all that, before doing anything, she must inform me.

**E: Does Amina have the power to sell an animal and go to the hospital or take a child to the hospital?**

**Scores**

Amina-3

Both-3

Boru-1

Reasons for both

R1: They have to consult each other before making any decision.

R5: They have to consult each other but the father’s decision is final. Even if he not around, she must find a way to inform him before making the decision to sell the animal.

R7: They play different parts; the father gives the authority and the mother takes the child to the hospital. They need to consult each other.

R8: The father is responsible for his family wellbeing so he is the one to make the decision.

R3: If she is alone at home, she can do anything to save the life of that child. Other than that, if the father is around, she must consult him.

*(Birds chirping)*

R4: If the father is not around, she is the one responsible for taking care of the household and the livestock. She can sell the livestock and take the child or herself to the hospital, if the animals need medicine, she can buy medicine and all the other house hold needs. When the father is around, he takes back his responsibilities.

Reasons for Amina

R3: so that Amina can be in position to respond to an emergency which is sickness

R4: this is because amina equally has the capacity to make decisions that impact the family positively. She cannot just look on and die or when the children need medical attention

Reasons for Boru

R1: this is because he is the man and the overall decision maker within the household.

**E: I have another short story. There are two people. They are husband and wife. The husband’s name is Adan and he is 45 years old and his wife’s name is Shariffa and she is 40 years old. They own livestock like cows, goats, sheep and camel. They have been married for 3 years. There has been a disease outbreak which affects both humans and livestock, the disease has been recurring for the past four years. Shariffa has been invited to attend a seminar about the disease. I have a few questions and I would like you to answer using the cards. My first question is, Does Shariffa have the power to attend the training?**

**Scores**

Sharifah-0

Both-4

Adan-3

*(Children playing at the background)*

Reasons for both

R1: They have to consult each other before she attends the seminar. She cannot go without her husband’s permission.

R3: consultation is done to avoid conflicts in the home

R4: That is how our culture and religion want. We have to consult, and I let her go.

**E: So, she a man can let his wife attend the seminar even if it is outside your village or within your village?**

Chorus: Yes, they can.

R5: If we consult and agree she can leave otherwise she stays.

**E: What if she goes without telling the husband?**

Chorus: She will get married to that seminar.

R5: Her Talaq will be sent to her. She will not return home.

Reasons for boru

R2: A woman cannot go out without informing the husband because he is the household head.

R4: The reason why she needs to consult the husband is because she is his responsibility traditionally and religiously. She is under his care. If anything happens to her, he is the one who will be asked. She has to consult her husband.

R1: the Islamic custom states that a woman is under a man and the man id entitled to escort/accompany the woman wherever she is going as a husband and sign of protection
