# Supplementary material for: “Without a man’s decision, nothing works”: Building resilience to Rift Valley fever in pastoralist communities in Isiolo Kenya
Source: PLoS One. 2025 Jan 28;20(1):e0316015. doi: 10.1371/journal.pone.0316015 (PMC11774392; doi:10.1371/journal.pone.0316015)
Supplement: S1 Dataset — (ZIP) [file pone.0316015.s001.zip › Supporting Information Files/File 6.docx]

**E: The first thing I would like to ask you is (cock crowing) what kind of livestock you keep?**

R4: Cows and goats

R3: Goats, camels, chicken, and donkey

**E: I would like you to tell me what is owned by men and what is owned by women.**

R5: Women have cows and goats, and they take care of them.

R7: Women are the sole caregivers regardless of whether they own the animals or not.

**E: In Ownership, which types of animals are known to belong to men, and which are known to belong to women?**

R7: Women are known to own donkeys only.

**E: Donkeys only?**

R7: Yes, all the other animals are known to belong to men.

*(Cock crowing)*

R7: What is clearly known to belong to women is donkey and chicken.

**E: Our first question is, what type of diseases infect livestock and humans in this area?**

R5: The main problem is fever. Diarrhea and vomiting and also chickenpox.

R8: In animals, they have diarrhea and urinating blood.

*(Sound of a child playing)*

R7: Cattles die from a very high fever.

R1: Vomiting and diarrhea

*(Birds chirping)*

R3: In animals, they have a fever, diarrhea, bleeding from the mouth, and urinating blood.

R2: Many people get a fever from eating meat from cattle that had a fever but got vaccinated before slaughtering.

R1: Sometimes you get sick from drinking milk from an infected animal that has been vaccinated.

R3: Are you asking about the diseases that affect humans or animals?

**E: Both humans and animals.**

R3: The diseases that affect animals will also infect humans because humans feed on animals and animal products.

**E: This fever you have mentioned, is it from malaria and other diseases, or is it from RVF?**

R1: The fever is from other diseases. There is also a fever from RFV disease, but we have not experienced it in the recent past.

**E: How does RVF disease affect humans and animals?**

R1: Animals bleed from the mouth, humans too bleed, diarrhea for both.

**E: So, you clearly understand the RVF disease, right?**

All respondents: Yes.

**E: Please tell me the symptoms of RVF disease in animals.**

R1: The animals shiver it’s like it feels cold.

R4: Animals cough

R3: They start dying. Their young ones especially cows’ goats and sheep.

R5: Bleeding from the nose. When the bleeding starts, there is no going back it will die.

R1: Their legs become numb, and they are unable to walk.

**E: What else?**

R3: Goosebumps

R7: Diarrhea

R2: Blood diarrhea.

R1: Dulling of the eyes

R8: animals miscarry a lot

R4: Also, the fever is too high.

R2: Their nails start to break in vertically.

*(Women discussing in low tones)*

**E: Which is the main symptom that clearly shows that an animal has been infected by RVF? Please raise up your hands if you say numbness.**

R1: An animal can cough and still walk but the other one cannot move an inch it might even die of hunger.

**E: From our discussion**, it shows that the first main symptom that tells you if an animal has been infected by the RVF disease is Miscarriage followed by bleeding from the mouth and nose. It is followed by fever. Fever is followed by the dullness of the eyes, followed by goosebumps. Numbness is the last on our list.

**E: How does the RVF disease affect humans?**

*(Sound of a crying child)*

R2: Fever

R4: Stomachache and diarrhea.

R5: Headache

R1: Joint pain.

R3: Tiredness

R4: Loss of appetite.

R1: Vomiting

R8: Dizziness

R6: Weight loss

**E: From our discussion,** the main symptom of RVF disease in humans is fever followed by loss of appetite and it is followed by headache, (voice of a man greeting people) followed by vomiting, followed by joint pain, followed by diarrhea.

**E: Our second question is how humans and animals get infected by RVF disease.**

R4: Humans get the infection from eating the meat of an infected animal.

R3: Humans get infected by drinking milk from an infected animal. The milk is not tested and usually drink raw milk.

R1: There is no way to detect an early infection in the animal we realize it when the symptoms have advanced so we get infected without notice.

R7: Just like they’ve said we get from meat and milk.

**E: How do animals get infected?**

R7: When they are hit by hunger, they become sick.

**E: We are talking about RVF disease.**

R1: No, it does get infected when hungry, that is something else. The animals get infected when they have been bitten by the mosquitoes.

R6: mosquitoes.

**E: Is there something else you would want to add?**

*Silence.*

**E: How do you treat RVF disease?**

*(Sound of a child)*

R5: They are taken to the hospital

R3: We also use herbal medicine.

**E: Which are these medicines?**

R3: Walthenna, Mwarobaini (local herbs). We boil mwarobaini leaves and drink half a glass of it when it is cool. It cures the fever.

**E: What other herbal medicine do you use?**

R3: Walthenna. We soak it in water and drink half a glass at night. It cures fever.

*(Sound of a child shouting)*

**E: Is there any other type of herbal medicine that you use?**

R5: Haldheeth. It is soaked and drank in the morning

R6: Marasisa; these herbs are smoked like a cigarette.

**E: You mentioned that you go to the hospital, Is it a private or a public hospital?**

Chorus: Public

R6: They don’t have a laboratory to test people. You are given painkillers like Panadol.

**E: is there any other kind of treatment?**

R7: A herbal medicine is known as biress.

**E: Please repeat**

Chorus: Biress.

R6: herbal medicine doesn’t have a prescription sometimes people overdose and die.

R5: Quran is recited for the sick.

**E: Other than going to the hospital and use of herbal medicine, is there any other way you use to prevent yourself from getting infected by the RVF disease?**

R1: When it rains, grass grows very fast. I cut the grass and sleep in mosquito net and also drain away stagnant water to prevent mosquitoes from breeding.

*(Someone coughing)*

R8: I don’t stay out at night, and I sleep under a mosquito net to prevent myself from being bitten by mosquitoes.

**E: How do you prevent yourself from getting the infection from the animal?**

R5: We burn firewood or anything that smokes to prevent the mosquitoes from biting the animals.

R8: Sometimes we put up nets for the young animals.

R4: we don’t eat meat or drink milk from an infected animal until the animal is fully recovered. And when it dies, we dispose of it in far places.

R1: I boil the milk and drink. I also boil the meat, pour out the soup fry the meat and eat.

**E: Why do you pour the soup?**

R1: Because all the bacteria will be removed from the meat, and it will be in the soup. I will be safe.

R5: The way she just said, when you boil meat, everything that was in the meat will be transferred to the soup, so it is safe to eat the meat than to drink the soup.

*(Sound of a motorcycle passing)*

**E: Is there any other way you prevent yourself?**

Silence

**E: When an animal miscarries, you said you dispose of the remains. How do you prevent yourselves from getting infected?**

R2: I don’t have gloves; I wrap some clothes on my hands and carry the remains and throw them away together with the cloth.

R5: We cannot get masks or other protective gear in our area. Our area is remote. We believe that God will protect us and continue with life.

**E: Do veterinary doctors come around your area and vaccinate the animals?**

R4: Yes, they do but it’s been a while since they came around.

*(Women discussing in loud voices)*

**E: If you are told about a certain disease which is in the region, and you are told to vaccinate your animals would you do it?**

R5: There are people who would and people who wouldn't.

**E: Does the vaccine help?**

R3: Some animals die after being vaccinated.

*(Sound of a child playing)*

**E: Of all the measures we discussed earlier on how to prevent ourselves from being infected by the RVF disease, which one do you think is the most effective form of prevention.**

Chorus: The use of Mosquito net.

*(Women discussing)*

**E: I am talking about the preventive solutions you had earlier mentioned.**

*(Women continue to discuss)*

*(Sound of a child crying)*

R5: Cutting of the grass in the compound.

**E: What follows the cutting of grass**

*(Sound of a child crying)*

R6: Draining of the stagnant water.

*(Someone coughing)*

*(Sound of a child crying)*

**E: Now I would like to tell you a story and I request you to listen carefully. We will have a discussion later. There are two people. One is Amina and the other one is Boru. They are a married couple. They own livestock. These livestock are cows, camels, goats, and sheep. Are we together? In 2023, there was a disease that was a disease outbreak in their area which infected both livestock and humans. Our first question of discussion is how Amina can and Boru prevent themselves from being infected by the disease. Please hold these cards. This is Amina, this is Boru, and this is Boru and Amina. So, we will answer the questions using the cards. If you think it is Amina, you hold Amina’s card. If it is Boru, you hold his card and if you think it is both, you hold the other card. Are we together?**

*(Women discussing) (Someone coughing)*

**E: Does Amina have the power to take the livestock to the market and sell it?**

Scores

Amina-

Boru-

Both-

Reasons for boru

R1: I chose that because when a woman is married and the husband is present, she cannot make any decision on her own.

Reasons for both

**E: Please tell us why you said that they will make the decision together.**

R2: They are married, and they have to make the decision together. She cannot do it alone.

*(Women discussing in low tones)*

R3: I chose the husband. The husband as the head of the house has the right to all the livestock. Women are not involved in anything. They are just informed when everything is done.

*(Motorbike passing)*

R4: I chose the two of them because they lie together, and they handle everything together. If they decide to sell part of the livestock, they must discuss it because it is a family affair. The livestock belongs to his children and us too.

R5: I chose Amina because the livestock belongs to both, she can sell it when he is around and when he isn’t.

R8: The livestock belongs to the husband. The woman cannot sell the livestock because it doesn’t belong to her

*(Sound of children playing)*

R7: We will sit and consult each other and what we will decide on is what will happen otherwise there will be fights between us.

**E: So, when he sells without your consent, what will happen?**

R7: Yes, and I sell without his consent he will also be mad at that is why we consult each other. We sit and talk it out or if the fight is huge, we call an elder who will sit with us and offer guidance and help us make peace.

**E: The elders that you invite, are they relatives or someone from outside?**

R7: we start with our family members and if the fight is not resolved, we involve the community elders.

**E: When Amina falls sick, does she have the authority to go to the hospital or she must ask permission from her husband?**

*(Women discussing in low tones)*

**E: Six of you have said that Amina needs to seek permission while two of you say that she can decide it on her own. I would like you to tell us why you made those choices.**

R1: Amina is married, they have livestock together, and they live together so she has to ask for permission from her husband before doing anything or going anywhere.

*(Birds chirping)*

R2: She cannot go to the hospital without informing him.

R3: She cannot go without her husband’s permission.

R5: She needs to consult her husband because she might need a lot of money for her treatment, and she doesn’t have money or also she might be referred to another hospital. She cannot do all that without her husband’s consent.

R4: She can do it by herself. For example, if they had a fight, and a child is sick, she can take the child to the hospital without telling him anything.

R8: I can call my husband to ask for money that I might need to use at the hospital that is how he will be informed.

**E: Lack of money is what makes you give him a call?**

R8: Yes.

R7: I cannot go anywhere without informing him.

**E: Why**

R7: Our religion Islam teaches us to obey our husbands.

**E: What about you R6?**

R6: My husband is the head of my household, he is my leader, if I need to go to the hospital, he is the one who will take me.

**E: If they sell the livestock, can Amina use part of the money to start a different kind of business or she has to have her husband’s consent to do so?**

R1: She needs to consult her husband because the money belongs to both.

R2: It cannot happen without the husband.

**E: Why?**

R2: He must give the money because he is the head of the family.

R3: It is the husband who is the decision maker. It ends with him. He owns the money and if he wants to give it to his wife, he can take the money from the account and give it to her. E is the main decision maker.

R4: We will consult each other because the money belongs to both of us and come to an agreement on how to use the money or on which type of business, we shall invest in.

R5: Business is something that has a lifeline, and it is risky. (Sound of a child playing) and you need to consult each other so well.

R7: We need to sit as a family and decide on how the money should be spent.

R6: We need to consult before doing any business and since we have children, they must be part of the decision-making process because at the end of the any decision made will affect them.

**E: I have another short story and I would like you to listen to it carefully and later we discuss it. There is a couple. The husband’s name is Adan, he is 45. His wife’s name is Shariffa, she is 40 years old. They have been married for 3 years. They live in an area called Mutta. In their area, there has been a disease that recurs after every 4 years, and it affects both livestock and humans. Adan and Shariffa are livestock keepers. They have camels, cows, goats and sheep. Shariffa was invited to attend a seminar about the disease. Using the cards, I would like you to tell me if Shariffa has the power to make her own decision and attend the training without informing her husband or she will have to consult him first. Or she will consult people that they live with.**

*(Women discussing in low tones)*

*(Sound of a child)*

**E: Why did you say that she has to consult her husband?**

R1: I need to consult my husband because he is the head of the household.

R2: I will consult my husband and if he permits me, I will go.

*(Sound of a child)*

**E: Why would you consult him?**

R2: He is the head of our household. He is the main decision-maker in our house. If he says that I should go, I will and if he doesn’t, I won’t go.

R4: I consult my husband and he decide whether I should attend or not.

R8: I will need to consult my husband.

R7: I cannot decide on my own. He knows where I am set right now, and it is because we consulted

*(Women discussing, laughing, and someone coughs)*

**E: Can both men and women attend the seminars?**

Chorus: Yes.
