# Supplementary material for: “Without a man’s decision, nothing works”: Building resilience to Rift Valley fever in pastoralist communities in Isiolo Kenya
Source: PLoS One. 2025 Jan 28;20(1):e0316015. doi: 10.1371/journal.pone.0316015 (PMC11774392; doi:10.1371/journal.pone.0316015)
Supplement: S1 Dataset — (ZIP) [file pone.0316015.s001.zip › Supporting Information Files/File 7.docx]

Enumerator: **R8 Which livestock do you own?**

Respondent 8: we have goats, cows, and camels (*children playing in the background)*

Enumerator: cows, camels, goats, and what else?

Respondent 8: Donkeys.

***(****Murmuring****)***

Enumerator**:** say your name and say it. You are R7.

(Women discussing)

R7 what do you own?

Respondent 7: we have goats, cows, donkeys, and sheep.

Enumerator: R6

Respondent 6: we have donkeys, cows, goats, and hens.

Enumerator: cows, sheep, goats, hens, and camels

Respondent 6: we have never owned camels.

Enumerator: there are no camels. **which one is owned by men and owned by women?**

Respondent: livestock?

Enumerator: **which is owned by men and owned by women?**

Respondent 3: cows are owned by men because they are the ones who herd. (*Children shouting)*, women own the ones which remain behind at home like hens. Women look after the one which remains behind at home during drought. (Women discussing and children shouting in the background)

Enumerator: R6

Respondent 6: They both own together.

Enumerator: who owns hens?

Respondent 6: women.

Enumerator: Donkey?

Respondent 6: women.

Enumerator: **My first question is, which disease affects both human beings and livestock in community?**

Enumerator: yes respondent 8, come closer (clearing throat)

Respondent 8: the diseases that affects cows is Trypanosomiasis, Haemorrhagic and Septicaemia

Enumerator: don’t be afraid you’re right

Respondent 7: hoyale

Enumerator: which other diseases?

Respondent 1: the diseases are Haemorrhagic Septicaemia, Trypanosomiasis

Enumerator: **do not repeat the ones that have been said, add another one, so that we can save time since its Friday**

Respondent: these are the ones

Respondent 6: Bottle jaw.

Enumerator: *budhaa*

Respondent 6: Bottle jaw in livestock causes difficulty when urinating

Enumerator: any other (child murmuring)

Respondent 6: the hoyale affects between the legs and tongue for livestock or human beings?

Enumerator: what diseases affects both livestock and human beings, if possible, mention which affect both livestock and human beings.

Respondent 4: for human beings, it is sirgo, vomiting and diarrhea

Respondent 3: vomiting and diarrhea

Respondent 1: where there is stomachache and then diarrhea follows up, there is malaria, through mosquito

Respondent5: diseases like silisa it is caused by hyenas after it bites livestock

Enumerator: **Have you heard of rift valley fever?**

All respondent: Yes, RVF in humans and livestock

Respondent 2: during the rainy season there is Gandhi, kala-azar. Kala azar it swells liver, stomach when someone consume infected livestock, they also contract disease. During drought season there is no pasture hence it becomes a problem to the livestock

Enumerator: What are the signs and symptoms of RVF

Respondent 1: you will know rift valley fever in livestock (cows) when the cows shiver, changes in skin, lack milk production, lacks appetite, so we say it is rift valley fever and inject it antibiotics it can’t say am sick

Enumerator: it can’t say am sick. Any other behavior? How would you know it has rift valley fever?

Respondent 8: it has fever and shiver

Enumerator: anything you can add, respondent 8

Respondent 2: if it has rift valley fever and you will you isolate it from other livestock

Enumerator: apart from fever and shivering, what else can have?

Respondent 7: lack of appetite and unhealthy

Respondent 3: running nose

Respondent 4: blood in urine, that they say it is rift valley fever

Respondent 6: stillbirth

Respondent 5: Rift Valley fever is during rainy seasons and not drought season. During drought season except for some diseases like fever livestock are good

Enumerator: the sign you have mentioned is shivering, fever, stillbirth, bloody urine, runny nose, and lack of appetite

Enumerator: **Signs and symptoms of RVF in human beings?**

Respondent 1: fever, joint pains

Respondent 2: severe headache

Respondent 3: stomachache/diarrhea

Respondent 4: it causes death, other have survived after seeking medication. When it affects who take care of livestock it can kill faster

Respondent 5: Death

Enumerator: according to your responses you will know rift valley fever by death first, fever and severe headache, diarrhea, and joint pains

Enumerator: **Our third question wants to expose how RVF is transmitted.**

Respondent 1: it spreads when the area is infested with mosquitoes, it spreads by biting it brings fever, it also spreads by biting livestock

Respondent 2: mosquito is found during the rainy season but during the drought period

Respondent 3: milk from livestock, you know we don’t boil it when we consume it, and that’s how it spread

Respondent 6: we just consume milk without boiling

Respondent 4: through meat, we cook the meat and drink the soup. You can get it through that way

Respondent 8: by consuming the meat of infected livestock (murmuring)

Enumerato**r: how does it spread from livestock to human beings?**

Respondent 3: during the rainy season, contagious diseases such as Caprine pleuropneumonia it occurs all season

Enumerator: **what about rift valley fever?**

Respondent 3: rift valley fever occurs during the rainy season

Enumerator: **rainy season**

Respondent 3: the rainy season where there is long grass

Enumerator: **and you respondent 8?**

Respondent 8: (clearing throat) It’s during rainy seasons when there are mosquitos

Enumerator: **and you respondent 2?**

Respondent 2: rainy season

Enumerator: **my fifth question, is how do you treat people affected by rift valley fever?**

Respondent 5: you take them to the hospital and the sample taken

Respondent 6: no, we are not medical officers. We just know it just has a fever

Respondent 7: religion leaders pray for the sick by reciting the Quran

Respondent 8: To be prayed for since he is sick that’s why

Enumerator: **Which hospital do you go to? Is it private or government?**

All respondents: government

Enumerator: **government, which other treatment method do you use?**

Respondent 1: use of herbal trees. when someone has a fever, we grid the herbal trees and make him/her drink and make the sick sweat to clear the fever

Enumerator: **any other herbal medicine you use?**

Respondent 2: we only use *waldhen* and *aldhid* when fever persists for long, we slaughter and use the hides as a treatment

Enumerator: **why use the hides and skin?**

Respondent 2: They help the patient to get well

Enumerator: **is it dried, or do you use it when it is fresh?**

Respondent 2: when it is fresh

Enumerator: **is there another any way of treatment? You have mentioned traditional medicines, religious leaders, and hospitals**

Respondent 4: the treatment is in two ways; one is the hospital, and another is the use of hides. We only know two ways of treatment (child crying and woman murmuring)

Enumerator: **as a community what are the measures you use to avoid these diseases?**

Respondent 5: only God can keep it away from us

Enumerator: **yes, God can keep it away from you but there is something you can do to avoid the disease**

All respondents: the thing that we use is herbal medicine and we are taking measures like keeping the infected animals away since it can be transmitted through them (chorus) it can be transmitted through water

Enumerator: **now you are not affected, but how can you prevent the spread of this disease? what are measures you use to prevent this disease?**

Respondent 4: we go to the hospital (inaudible murmuring), we boil and treat water since we use water from boreholes and water pans which we boil it and also treat it

Respondent 2: without treatment it can cause stomachache and diarrhea, so by treating it prevent rift valley fever

Enumerator: **what are the other ways you use to prevent this disease?**

Respondent 5: use of mosquito nets

Enumerator: **use of mosquito nets, is there another way? How do you prevent these mosquitos?**

Respondent 7: we use mosquito nets, back in the day we use to migrate to another place

Enumerator: **without migrating and use of nets which are the other ways?**

Respondent 8: we use smoking methods whereby we lit the fire by adding dry leaves to produce smoke thus reducing mosquito (inaudible response)

Enumerator:

Respondent 3: boiling milk and also cooking meat but you don’t drink the soup

All respondents: You cook the meat and because all nutrients are in the soup you don’t drink the soup

Respondent 7: you wear the protective gear

Respondent 5: livestock vaccination to reduce the spread of this disease

Enumerator: **of all these ways you have mentioned, which one is helpful? Boiling of milk, use of nets, wearing gear, and vaccination which is helpful?**

Respondent 3: vaccination when you vaccinate it reduces the spread of this disease

Respondent 6: nets

Enumerator: **why nets?**

Respondent 6: to prevent mosquitos

Enumerator: **what next after nets?**

Respondent 7: gloves

Enumerator: **why this?**

Respondent 7: to reduce dirt and getting in contact with body fluid

Enumerator: **what next?**

Respondent: boiling milk

Enumerator: **why boil milk?**

Respondent 2: to prevent the disease

Enumerator: **what next after boiling milk?**

Respondent 1: we consume meat after pouring soup thus will prevent the disease

Enumerator: **why pour the soup?**

Respondent 3: because the soup has nutrients, because the livestock is infected you cook the meat and pour the soup

Enumerator: **I will give you these cards and tell you what to do with them (clearing throat), now I will narrate a short story listen carefully. There are two people, how many people? husband and wife. The husband is called Boru, and the wife is called Amina, they have livestock, they are pastoralists, and they own cows, camels, goats, and sheep. In 2023 they were affected by the disease in their community. For instance, the disease affects both humans and livestock, are we together? Respondent 2 have you understood? this disease affects livestock, the husband and wife live together, and they own livestock, how will this ownership of livestock help them to prevent this disease? That one. We have said Amina is Boru’s wife will she be able to sell this livestock understood?**

All respondents: yes, we have understood

We want to know whether Amina is able to make a decision, whether Boru makes the decision or whether they decide both. I hope you have understood. The cards have given you one is Amina the other is Boru and this is both together, now let the cards face this way, hold the three cards this way, have you understood, one is the husband other one is a wife and the other is both of them. Don’t discuss and look at each other’s cards show me cards the white side and what you think

Enumerator: when I say show me the cards, you show me the white side, (Boru) You rise the cards you have selected, my first question the disease affects the community and Amina wants to sell the livestock will she be able to sell by herself or its Boru who decides? Or both will decide? I have raised mine (Boru) just raise until she says show me. If it’s Amina you raise Amina’s card, if it’s Boru raises Boru’s card or if it’s both of them then you raise it (counting). Now you tell why it is Boru who can give permission which Amina is not able to.

Does Amina have the power to make the decision to sell livestock due to the RVF in the community to avoid losses?

Scores

Amina-0

Boru-5

Both-2

Reasons for Boru

Respondent 5: the reason Boru decides he is the household head. Amina she is a wife does have any power to do that.

Respondent 6: he is the head of household

Respondent 7: yes, because he is the head of the household

Respondent 2: because he is head and responsible for everything like if someone is sick, to look for food at times he shares the decision with her when he is around

Respondent 3: he is the owner of the livestock

Respondent 2: the owner does everything about the livestock

Reasons for both

Respondent 8: the reason is they are husband and wife, and there is no way he can decide for himself, when they have debt, paying school or even food they will decide together

Respondent 4: if he decides and she doesn’t agree, misunderstanding will a rise

Reasons why Amina cannot decide

All respondent 4: Amina will not be able to sell by herself because she is under the authority of the man

Access to Health

Scores

Amina-0

Boru-4

Both-3

Reasons for Boru

Respondent 8: Her husband has authority over her, so when she wants to go somewhere, they will sit together and decide

Respondent 5: he is the household head.

Respondent 2: he is the household head, so the wife cannot decide.

Respondent 1: without him nothing works, whatever he says is right such as taking to children to school or the hospital.

Reasons for both

Respondent 6: when children are sick you take them to the hospital without their father’s consultation. children belong to both of them so they will decide together

Respondent 7: the reason is, the children belong to them, and they are sick, she will discuss with the husband and decide if to sell the livestock and take the children to the hospital.

Respondent 3: they gave birth together, so they will decide together.

Return your cards.

Venture in other business

Scores

Amina-2

Boru-0

Both-5

Reasons for both

R8: they can decide together.

Respondent 3: they both own the livestock

Respondent 8: the reason is they both have children together so it’s also hers.

Respondent 2: one is the husband and the other is the wife, they can decide together. If can decide alone, there will be a misunderstanding.

Respondent 5: they are husband and wife, for instance, If I say this is mine and do whatever I want misunderstanding will arise, so to avoid the misunderstanding I will discuss it with my husband. We decide and do business together.

Respondent 6: they both own the livestock, so they will decide together. (Children speaking)

Respondent 7: they decide together because, without the household decision, you cannot sell the livestock.

Reasons for Amina

Respondent 4: the reason she can decide alone is that she can own parts of the livestock.

Respondent 1: she is the household woman/wife, and she is mandated to have a share in the household/marriage

Enumerator: okay, I will tell you another story, listen carefully.

There are two people, husband and wife, the husband is Adan. The husband is called. Adan, Adan is 45 years, and his wife is called Sharifah, Sharifah is 40 years old. They have been married for three years. They lived in Mutaa, are we together? I have said that there is a husband and wife, the husband is Adan. Adan is 45 years old; his wife is called Sharifah, Sharifah is 40 years they have been married for three years, and they live in Mutaa. They are pastoralists, they own cows, camels, sheep, and goats. In the last four years, their community is affected by the disease; the disease affects livestock and human beings. Sharifah is invited to a seminar. Do you go to seminars? Sharifah was invited to a seminar (child speaking) will Sharifah be able to go seminar or Adan will decide? Or both will decide? I will add other cards. (Child speaking)

Does Sharifah have the power? If she has, you say yes, if she doesn’t and Boru, Adan’s has, you show me Adan’s cards or they discuss together, you show me that one. Sharifah will attend the seminar to get knowledge about the disease and also to create awareness. Are we together?

Enumerator: have your cards with you, show me the cards if Sharifah will be able to go to the seminar. Or whether Adan will decide, or they will decide both

Scores

Adan-3

Sharrifah-0

Both-4

Reasons for Adan

R5: Adan will decide for Sharifah to go to the seminar because he is the husband and head of the household

R3: Adan is the household head. In our culture wife cannot go without her husband’s consent.

Respondent 2: her decision alone cannot be. She cannot go anywhere without her decision.

Reasons for both

Respondent 1: she cannot go anywhere without him knowing and same to him, he cannot go without informing the wife that he has been invited for a seminar and he would like to attend. They will discuss and when they reach an agreement, he tells her to go, if they don’t agree she won’t go.

Respondent 7: they both have the same responsibilities.

Respondent 4: the reason is that they are both the same people. We discuss together, you won’t go anywhere without him knowing. (*Murmuring and children playing)*

Enumerator: do men and women in the community attend the seminar?

Respondent 2: we are not invited but when we are invited elderly people like me do attend it.

Respondent 8: When there is a seminar, we attend

Respondent 7: we go we go

Enumerator (Boru): do both men and women attend seminars equally?

Respondent 1: we attend equally.

Enumerator (Boru): We have eight people here, so four must be women.

Respondent 6: no, both men and women attend.

Enumerator: when eight people are needed, how many women will get the chance?

Respondent 5: three.

Enumerator: most are men.

Do women have knowledge about RVF?

All respondents: those who attend the seminar have the knowledge.

Enumerator: as women and men, there are some measures to prevent this disease. What hinders such prevention in the community? (Factors that affect successful prevention of RVF)

Respondent 3: lack of time.

Enumerator: what are those that hinder such prevention?

Respondent 4: lack of net.

Respondent 5: lack of medicine.

Enumerator: you said lack of medicine, what else?

Respondent 6: as you see we live in a rural area and we only have one doctor, you can be sick for a long without getting medicine.

Enumerator: what else? R6.

Respondent 2: lack of medicine, our dispensary is not well stocked. There are no medicines

Respondent 1: there is nothing else. You light a fire to prevent mosquitos.

Respondent 7: We don’t have vet officers. RVF occurs only once. This is the period that it occurs.
