# Supplementary material for: “Without a man’s decision, nothing works”: Building resilience to Rift Valley fever in pastoralist communities in Isiolo Kenya
Source: PLoS One. 2025 Jan 28;20(1):e0316015. doi: 10.1371/journal.pone.0316015 (PMC11774392; doi:10.1371/journal.pone.0316015)
Supplement: S1 Dataset — (ZIP) [file pone.0316015.s001.zip › Supporting Information Files/File 8.docx]

**Enumerator**: as a resident of this area, what are the main livestock kept by the area resident? Please say

your number and response

R8: cows and sheep

**Enumerator**: what is your response, respondent 1?

R1: sheep and goats

Enumerator: what is your response, respondent 2?

Respondent 2: goats and sheep

Enumerator: what about you respondent 3?

R3: cows and goats

Enumerator: Does anyone with additional points or opinions?

R1, R2, R3, R7: (chorus) We mostly keep cows, sheep, and goats. Let’s not use or repeat the answer if you

have the same response as your partner

Enumerator: any other animals apart from the ones you have mentioned, what about poultry?

Respondent: All (chorus) We don’t rear chicken commonly

Enumerator: are there camels?

Respondent: All (chorus) We don’t rear camel as well

Enumerator: what are the most reared livestock by men in their community?

R2: cows and sheep

R3: (low tone) Cows and sheep

Enumerator: do not repeat the same response as your colleague. Do women own livestock?

R2: yes, they do

Enumerator: what are these livestock they keep?

R1: chicken

R6: donkey

R7: same as R1 and R6

Enumerator: why do men rear cows and sheep while women rear chickens and donkeys?

R6: they keep donkeys because they help in the transportation of firewood water etc,

Enumerator: why do women don’t own animals like goats and cows?

R3: they own it but not as many as men’s counterpart

Enumerator: where do they get it? Do they buy them, or they are given them?

R8: they are given by their fathers probably before they get married and as such, they own it entirely

R5: maybe they buy from their savings

Enumerator: to start with, are there diseases that affect both humans and livestock?

R1: (malaria) fever, which affects both human and livestock

Enumerator: what about you R8?

R8: rift valley fever, this affects both humans and livestock

Enumerator: what about you R3?

R3: body fever

Enumerator: apart from fever, what about other diseases do you know?

R7: diarrhea

Enumerator: what is your opinion R6?

R6: do you want me to start with human disease or animal disease?

Enumerator: you can start with any of them, you are at liberty to start with the one you want

R6: let me start with human disease

Enumerator: go ahead R6

R6: typhoid

Enumerator: what is your response R5?

R5: (laughter) Mummering…

Enumerator: R5 could you kindly repeat your answer, we didn’t hear your response kindly

R5: malaria spread by mosquitos

Enumerator: R6, are there any human diseases that you can tell us?

R6: Kala azar (leishmaniasis)

Enumerator: what about animal disease?

Respondents: (laughter and sound effects in the background that made their response not to be heard)

R7: respiratory diseases like Asthma (pneumonia)

Enumerator: are we done with animal diseases? Is there any that we did not mention R1?

R1: (repeatedly) malaria

R8: TB

Enumerator: R6, please tell us about animal diseases

R6: I don’t know much about any disease honestly

R1: silisa (fever)

Enumerator: any other?

R8: dysentery (blood in the stool)

Enumerator: what other signs do they have besides bloody diarrhea?

R6: bloody diarrhea is always accompanied by uncontrolled saliva from the mouth

R5: other leaps

Enumerator: any other disease that we have not mentioned?

R6: kurtubale

R3: Gandhi caused by the tsetse fly this is spread by insect

Enumerator: let us focus on rift valley fever, do you know about it?

Respondents: (chorus) Yes, we know

Enumerator: **what is the sign of the symptoms of this rift valley fever**? Tell me the signs and symptoms of rift valley for both humans and livestock, starting with

**Signs in humans**

R4: fever is the common sign of rift valley fever in humans, with severe headache and general body aches

Enumerator: R4 has said that there are general body aches, who can add more?

R8: joint pain

R1: fever

R3: joint pains

R3: blood coming from mouth and nose

Enumerator: let us go to the symptoms of rift valley fever for livestock.

**Signs and symptoms of rift valley fever for livestock**

R1: retained placenta

R2: bloody urine

R6: lack of appetite

R4: mass abortion by animals

Enumerator: any other? Those of you who are sitting on this side are silent

R7: the affected animals cannot move and hence remain in the Boma the whole day

Enumerator: In the next question, we are going to discuss the transmission cycle of RVF. Can you tell me the mode of rift valley fever transmission? Let us respond kindly.

R2: if one takes either milk or meat of infected animals

R1: if you touch an infected animal in the mouth

R8: if you touch the carcass of the animal that died of rift valley fever

R4: if you are beaten by a mosquito that carries the virus from the infected animals

Enumerator: what other modes of transmission do you remember?

R1: we have mentioned the one we remember

Enumerator: how did you hear about the rift valley fever and how did you know?

R2: someone from our location has died of this disease we also learned about this disease through CDR and sensitization sessions during public barazas by healthcare workers and local administrators

Enumerator: what are some of the ways that you can identify rift valley fever in animals and humans?

R8: through blood sample testing

Enumerator: who does this blood testing?

R1: veterinary officers

Enumerator: how do you know it is indeed rift valley fever?

R2: abortion

Enumerator: what are some of the activities that can help us contract rift valley fever from animals?

R3: women

Enumerator: why is milking the role of women? Why not men? Why not children? Why only women?

R3: because they are caregivers and such, they use that milk to feed children and cook tea for the

family members. Males look after the animals, but the responsibility of milking does not belong to them

Enumerator: any other activities that spread rift valley fever from animals to humans?

R1: feeding of animals

Enumerator: who does the feeding?

R1: women

Enumerator: why women?

R1: they are generally the caretakers of sick and weak animals at home

Enumerator: why is feeding not done by men or children?

R6: children go to school, and they also cannot be able to do this hard task. Men on the other hand have other responsibilities like herding and watering animals and therefore cannot multitask

Enumerator: during feeding can the animals spread the rift valley fever to the feeder?

R3: yes! The mosquito that bites them also bites the one feeding the animals and thus passes the disease to the one feeding. The mosquito that bites them at night bites the human beings during the time of feeding and therefore spreads the disease to the human

Enumerator: the other activities that spread rift valley fever from animals to human beings?

R5: during spraying of animals with insecticides

Enumerator: how is rift valley fever spread during spraying?

R6: any activity that brings you closer to the animals that has rift valley fever can easily expose you to the disease and you can become affected

Enumerator: who normally does spray this animal?

Respondents: any member of the household can do the spraying. It is not a specific person’s responsibility to spray the animals against pests and diseases. A man can do what a woman can do, any adult in the Boma can do expect children

Enumerator: when do women normally do spraying?

R3: when there are no men around

Enumerator: why is spraying mostly done by men?

R6: it is a hard task that needs to be done by men unless they are not available

Enumerator: any other activity that can transmit rift valley fever from animals to human beings?

R1: when handling animals specifically during vaccination and deworming

Enumerator: whose responsibility are those?

R2: it is the collective responsibility of a man and a woman to do together. It is not a man’s responsibility or a woman’s responsibility is any person’s responsibility and not for a specific gender

Enumerator: who does it mostly?

R8: vaccination and deworming are mostly done by men

Enumerator: what other activities can spread rift valley fever from animals to human beings?

R3: during cleaning of animal wastes

Enumerator: whose responsibility is that?

R4: girls and women

Enumerator: why is the cleaning of animal wastes the responsibility of girls and women?

All Respondents: cleaning is culturally the responsibility of women and thus a man cannot clean in the presence of female gender around them

Enumerator: (laughter) That is exactly what am trying to find out. During the rift valley fever outbreak, who treats those who get rift valley fever? How are they treated?

R2: they are treated at the health facilities by nurses. They also vaccinate animals.

R3: (laughter) I really don’t remember who treats it and how is it treated

Enumerator: after being discharged from the facilities, who takes care of the patient at home, and how is home-based care done?

R2: the person who normally prepares food for the family is the one who stays with the patient most of the time. In this case, mothers are responsible for them as they give recuperative feeding to the patient to recover quickly, mothers are also available at home most of the times and therefore are readily available to care for the sick person at the home

Enumerator: what other care can be given to the rift valley fever patients at home? R8 please respond…

R8: they need to be given a lot of fruits, and vegetables and need to be given the prescribed drug on times

Enumerator: who treats animals with rift valley fever?

R4: infected animals are treated by the animal owner because the animal belongs to them

Enumerator: you said earlier that sick animals stay indoors during the period of sickness, what care is these animals given at home?

R7: they are isolated and fed separately. Water is also given in the isolated room

Enumerator: who feeds them?

R1: care is generally given by the mother at home

Enumerator: which other care is given to animals with rift valley at home?

R7: they can also be injected according to the medicine as instructed by the experts

Enumerator: at what time is this outbreak of rift valley fever common in our area?

R2: during the rainy season

Enumerator: R6, what is your opinion on this?

R6: during the wet season

Enumerator: R4, any opinion on this?

R4: during the flood, especially in swampy areas

Enumerator: R6, I want to hear your response

R6: where there is stagnant water, there are insects that bite which causes the rift valley fever outbreak

Enumerator: R7, what do you have to say on this?

R7: during the rainy season when mosquitos are breeding. It bites which increases the rift valley fever

outbreak

Enumerator: what is your take on this R3?

R3: rift valley fever becomes common during the rainy season when tall grass grows giving the breeding grounds for the mosquitos

Enumerator: let R1 give her views too

R1: during the rainy season when mosquitos are very many

Enumerator: which facilities do you normally seek medication in? Privates or public hospitals

R6: there are no privates facilities around, we seek treatment from public hospitals

R7: we also seek herbal treatment from traditional healers

Enumerator: how is these herbal treatments from the traditional healers?

R6: they are mixed with tea

R8: we take soup

R1: herbal medicine can be taken with food

R7: others are taken directly with water

Enumerator: are there who self-medicate themselves? I mean those who treat themselves without

consulting medical expertise?

R3: yes! There are those who seek spiritual healing by asking religious leaders to treat them. The divine. They normally go to sheik or imam for treatment

Enumerator: are there people who seek traditional healers’ medication?

R3: yes! They do seek medication from them

Enumerator: what could be the reason for seeking their treatment?

R5: they believe that traditional healers equally treat what is treated at the hospital but with less

medication expense

Enumerator: what are some of these preventive measures to protect yourself from getting rift valley

fever?

R2: clearing away tall grasses which are the main mosquito breeding grounds, throwing away empty tins and containers that are acting like mosquitos’ hideouts

R3: proper cooking of meat before eating

R1: burning of animal wastes

Enumerator: do you boil milk as a prevention measure?

R3: yes! We do. The animal products like milk and meat from infected animals are a way through which Rift Valley fever can be transmitted to human

R4: we also vaccinate our animals during mass vaccination to prevent our animals from getting Rift Valley fever

Enumerator: (phone ringing) Please put your phones on silent mode now I want to know which of these measures work best in preventing the rift valley fever outbreak and why?

R1: mass vaccination is the best because prevention is better than cure and also it is cost-effective compared to the other measures

Enumerator: what are the second priority measures in preventing rift valley fever after vaccination?

R7: sleeping under a treated mosquito net prevents transmission of disease from animals to human beings as also from human beings to animals. Clearing of bushes also contributes greatly to the prevention rift valley fever

R1: boiling milk before consumption is also a very important preventive measure.

R3: boiling of water

**Enumerator: now we go to section B**.

Respondent: murmuring

Enumerator: you are tired? We told you we will take two hours. We have one hour we will be through.

Murmuring and chorus conversation, respondents laughing.

**Ownership and control**

I will narrate a short story. There is a person called Boru and he has a wife. They are pastoralists, they have sheep, goats, cows, and camels. There was an outbreak of disease in their community, that affects both human beings and livestock. This card is Boru, this is Amina, his wife’s name is Amina. These two Boru and Amina, are we together?

Respondent: mmh

Enumerator: everyone will tell me, there is no telling you will just show me with cards until I ask you why you are saying this or that. If I ask you a question and your answer is Amina, you hold your card till I ask you. Have you understood R8?

Respondent: Mmmm.

Enumerator: anyone who hasn’t understood?

Respondent 8: I haven’t understood.

Enumerator: I have said, if I ask you a question, R7 am repeating this because of you. There is a person known as Boru.

Respondent 7: Boru?

Enumerator7: yes, this is Boru. His wife is known as Amina. They are pastoralists and have livestock. They have goats, sheep, cow and camels. There was outbreak of disease in their area. They thought about selling their livestock. Now I will be asking a question, if you think it’s Amina you raise Amina, if I say show me you show me, if it’s both you hold this way. If I say show me you show me, are we together? If they want to sell their livestock, does Amina have the power to sell the livestock? Show me the cards. If you say Amina, has you raised Amina’s card. Not that way, raise your cards. If I say show me, you raise this way. Show me where there is picture, or you show me the white side. We must raise it the same time. Does Amina have the power to sell the livestock? If she has, raise the card. I want to see your cards. R6, you have raised which card. Now show me the card. Put the rest down.

Sale of livestock

Will she decide on her own? Will the husband decide? Or will they decide together?

***Boru-3,***

***Both-5***

***Amina-0***

Reasons for Boru

Respondent: 5 Because he is the owner of the livestock so he alone can make the decision

Respondent:4 He is the head of the household

Respondent 8: He is the owner of the animals and has the final say

Respondent: 6 he is the head.

Reasons for both

Respondent 2: the reason why I have chosen both is that the livestock belongs of both of us and

they share responsibilities together.

Respondent 1: livestock belongs to both.

Respondent 3: they got the animals together hence they have to make a joint decision

Enumerator: so next time you are supposed to pick this.

If there is a disease in their area and Amina wants to sell the livestock to go the hospital, is she able to make decision on her own?

Does Amina have the power to sell the livestock and go to the hospital (access to health carefor humans and livestock)? Will she decide on her own? Will the husband decide? Or will they decide together?

***Both-7***

***Boru-1***

Reasons for boru

Respondent:1 He is the husband hence he takes charge of all the decisions including access to health care for both livestock and humans within the household

Reasons for both

Respondent:7 Amina is a woman and has no say about the livestock which is why they must sit together and discuss to have a final decision on either to sell or not

Respondent: 2 They got the livestock together, so they must discuss as husband and wife and decide. So, both cannot make an individual decision

Respondent: 6 women can sell the livestock if the husband is not around.

Respondent: 7 They will discuss and decide because they are husband and wife.

Respondent: 8 am not able but he has the final decision.

Respondent: 5 has said does Amina have the power to sell the livestock and go to hospital.

Respondent 4: we understand each other that’s why we discuss together and by doing so she can go to hospital.

Enumerator: so next time you are supposed to pick this.

Amina wants to start a business because of the disease outbreak, can she sale the livestock and venture and others business. Will she decide on her own? Will the husband decide? Or will they decide together?

Scores

Boru-2

Both- 6

Reasons for Boru

Respondent: 2 Boru is the man and head of the household and all the decisions come from home

Respondent 3: He is responsible for all the needs in the home, if he doesn’t allow a woman to work, she must sit home

Reasons for Both

Respondent 8: they are husband and wife, and profit from the business belongs to them.

Respondent All, they decide together so there can be peace in the house. Joint decisions help to avoid conflicts in the home and individual decisions bring about conflicts which can cause separation.

Respondent 1: for mutual understanding.

Respondent 3: they are husband and wife.

Respondent 7: they have the same responsibilities.

Participation

There is a person called Adan and he has a wife called Sharifah. Adan is 45 years old and his wife is 40 years old. They have been married for three years. They are pastoralists. Their area has a disease which mostly affects human beings and livestock. Sharifah was called for a seminar. I want you to use the cards like before. Have you understood R4 and 5. Sharifah was invited for a seminar. Can she go to the seminar alone without telling anybody? If she can go, you show Sharifah. If she can’t go and Adan will decide for her, you will show Adan and if you say the two will discuss together you will raise both. Have we understood each other?

Respondent: mmmh.

Please repeat.

Enumerator: I have said, there is Adan and there is Sharifah. They are both pastoralists. There area is mostly affected with the disease, which repeatedly occurs maybe after three years. Sharifah was invited for a seminar. Have you understood?

Respondent: mmmh.

Enumerator: now I ask you, can Sharifah go to the seminar alone? If she can, you raise this one of a woman. If you say it’s the husband who makes the decision, you raise Adan and if they decide together you raise for both. Have you understood?

Respondents: mmmh

Enumerator: Will she decide on her own? Will the husband decide? Or will they decide together?

Scores

***Both 7***

***Adan 1***

***Sharifah 0***

Reasons for Adan

Respondent 8: he is the husband and he make decision. she is a woman, and you cannot let woman to go alone.

Reasons for both

Enumerator: you cannot let woman go alone. R5 why have you said they have to decide together?

Respondent 5: in Islamic religion, a woman cannot go alone. So, in case of such programs, both husband and wife have to discuss and agree.

Respondent 4: there is nothing else, if they agree she will go and if they won’t agree she won’t go.

Respondent 7: she won’t go without her husband’s permission. This is because she is just a wife who cannot make decisions on her own

Respondent 3: they are husband and wife, so they must agree on thigs that happen in the hh together.
