# Supplementary material for: “Without a man’s decision, nothing works”: Building resilience to Rift Valley fever in pastoralist communities in Isiolo Kenya
Source: PLoS One. 2025 Jan 28;20(1):e0316015. doi: 10.1371/journal.pone.0316015 (PMC11774392; doi:10.1371/journal.pone.0316015)
Supplement: S1 Dataset — (ZIP) [file pone.0316015.s001.zip › Supporting Information Files/File 9.docx]

Enumerator: we will start. My first question is, as a community what type of livestock do you own?

Respondent: cows, goats…

Enumerator: you haven’t mentioned your name.

Respondent: R5, cow…

Enumerator: 8 cows but raise your voice so that you can be heard from here.

Respondent: 2 cows, goats, sheep, camels, donkeys (*birds chirping)*

Enumerator: is there any other that he hasn’t mentioned?

Respondent:1 hen.

Enumerator: is there other that he hasn’t mentioned?

Respondent:3 there are many types of livestock, we keep pets such as cats.

Enumerator: Of the livestock that you keep, which belongs to women and which belongs to men?

(Birds chirping)

Respondents :1 The world is changing, as R1 has said, for instance, this household has a husband and wife, beside the husband owning it, it belongs to both of us. When we want to take children to school, we discuss how to pay the school fees.

Enumerator: R2 what opinion do you have?

Respondent 2: as R2

Enumerator: which livestock is owned by women and which is owned by men?

Respondent: 2 There is no separate ownership; both men and women do livestock husbandry. Men do herd and watering of livestock; they discuss if they are to sell the livestock.

Enumerator: R4 in your opinion do women own livestock?

Respondent: *(inaudible response)*

Enumerator: what?

Respondent: 4 In the household, women own something like chicken

Enumerator: hen is for women.

Respondent: yes, in a household but livestock is owned by men. Cows and goats, it’s for men

Respondent: All yes, it’s for men.

(*Inaudible conversation)*

Enumerator: my first question is which disease affects both livestock and human beings?

Respondent 1: As R1 the disease that can be spread from livestock to human beings is brucellosis

Respondent 5: There is Rift Valley that can spread from livestock to human beings and from human beings to livestock.

Enumerator: R3, tell us another disease.

Respondent 3: Rift Valley Fever and milk disease.

Enumerator: is there any other disease that affects livestock in your area except RVF and Milk disease?

Respondent 1: there are many diseases, it occurs in a season. Some disease affects only goats, and disease like Contagious Caprine Pleuropneumonia only affects goats and it occurs during the dry and rainy season. Disease like *Hoyale* affects cattle. During this drought period the disease that mostly affects human beings, like children is vomiting and diarrhea. (*Inaudible conversation).*

Enumerator: R2 do we have any other disease?

Respondent 2: Diseases such as *Hoyale,* Haemorrhagic Septicaemia.

Respondent 1: a disease called fuzni, which we didn’t have in our area in previous years kills cows and donkeys

Respondent: 5 There is a disease caused by the Mathenge tree. we don’t know its name we just called *forest disease*.

Enumerator: is there any other?

Respondent: 6 There is Gandhi, gasdor for goats, which causes fever in livestock. Hoyale disease which affects goats and cows.

Enumerator: R4 is their other diseases?

Respondent 4: these are the ones.

Enumerator: is there another they haven’t mentioned?

Respondent 4: marchakas affects goats and sheep whereby sheep become so thin,

Respondent 6: These are the disease that has been mentioned by my colleagues, there is fever brucellosis,

Rift Valley Fever (*clearing throat)*

Enumerator: How do you call Rift Valley Fever?

Respondent: *qando bini*

Enumerator: Now, how would you know the signs of Rift Valley Fever in livestock and human beings?

Signs and symptoms of RVF in livestock

Respondent: 3 livestock urinating blood.

Enumerator: livestock urinates blood. What else?

Respondent 1: (*birds chirping)* Livestock gives stillbirth, running nose with blood.

Enumerator: it has a running nose with blood?

Respondent: 4 yes, livestock also give stillbirth

Respondent: 6, Livestock has fever, running nose, shivers, during the evening it has a high fever, it produces warm milk

Enumerator: Warm milk.

Respondent 6: These are the ones.

(*Birds chirping)*

Respondent: 8 the meat is yellow.

Enumerator: Yes R2.

Respondent 2: it has watery eyes.

Enumerator: watery eyes.

Respondent 7: meat taste bitter/bad.

Respondent 7: it has goosebumps/shivering.

Enumerator: what else?

Respondent 1: you will know the difference between healthy and affected livestock, affected livestock produces different smell.

Respondent: 1 it has an unpleasant smell. Even the meat doesn’t seem to be fresh, inside the livestock’s stomach it has water, and the lungs have different colors.

Enumerator: Is there anything else? How does it affect human beings? We will start with R6.

Respondent 6: last year, I had a high fever, headache, loss of appetite, and pains in the joints. You won’t be able to sleep the whole-body aches. It causes dizziness and you won’t be able to stand up.

Enumerator: R5, is there anything that you can add?

Respondent 5: Rift Valley Fever?

Enumerator: Yes.

Respondent 5: Eyes turns to be yellow.

Respondent 3: retained placenta

Respondent: 2 if the disease persists, one bites the lips so hard. Everything that he takes is tasteless.

(*Murmuring and silence for long) (Coughing and clearing throat).*

*(Phone vibrating)*

Enumerator: We have been talking about the signs and symptoms of RVF in Livestock. So, what are the signs and symptoms of RVF in Humans?

Enumerator: Baba we are talking about Rift Valley Fever, we have said how we would know that livestock is with Rift Valley Fever. You have said it gives stillbirth, fever, running nose which has blood stains, watery eyes, blood in urine, meats are yellow, it has unpleasant smell, and you have said that, isn’t it?

Signs and symptoms of RVF in humans

Respondent:6 fever

Respondent:7 headache

Respondent: 8 nausea and vomiting

Respondent: 4 Body weakness and dizziness

Respondent 3: body aches

Respondent:5 yellowish eyes

Respondents: 2 lip smirking

Respondent:1 tastelessness of food

Enumerator: How does Rift Valley fever affect human beings and livestock? How does it spread?

Respondent:3 Drinking milk and eating meat from infected animals.

Respondent: R2, drinking the milk, eating the affected livestock meat

Respondent 1: Human beings get affected when helping livestock in giving birth bare hands without protection. Carrying stillbirth. Others drink milk without boiling it. Eating meat.

Enumerator: R7 tell me.

Respondent 7: mine is the same. Helping animals when giving birth

Enumerator: R4

Respondent 4: stillbirth, drinking milk from infected animals.

Enumerator: *(Inaudible conversation).*

Respondent: drinking milk from livestock that is infected with the disease and supporting the animals when having a stillbirth.

Enumerator: R5.

Enumerator: raise your voice.

Respondent 5: During watering livestock, healthy livestock can contract diseases from the affected livestock.

Respondent: 4 when healthy mixes with the affected livestock, the water point.

Respondent 8: When you eat meat from affected livestock. Drinking milk without boiling.

Enumerator: Anything else?

Respondent: 5 other thing is as he has said at water points, when healthy and affected livestock meet.

Enumerator: R1, do you want to add anything?

Respondent 1: Livestock can affect each other when you take one to the market.

Respondent: 2 when you come and settle in a cowshed where there was affected livestock, they can be affected through the livestock waste products.

Enumerator: next question, how did you get information about Rift Valley Fever? Or what is the source of information?

Respondent: when one is affected

Enumerator: when one is affected, he has a headache, fever, vomits, body aches, how did you know that he is affected with Rift Valley Fever and not any other disease?

Respondent: someone dies in our community, drinking milk and he becomes affected.

Enumerator: Now this disease how did you now.

Respondent: *(Chorus response)* R6, how we know.

Enumerator: how did you know Rift Valley Fever? Where did you hear?

Respondent: Livestock was affected, and it spread to human being, one was affected, he was taken to Merti hospital that was later referred to Isiolo. He was examined *(Inaudible chorus)* and he died. Then they came and examined his livestock. That’s the day R6 was examined and found to be affected.

Enumerator: so, it was the doctor who told you after they have examined the disease?

Respondent: All *(Chorus)* Yes, after it’s the doctors.

Enumerator: That’s what I want to hear

Respondent: 4 through baraza often called by the area chief

Respondent 1: Medical officer tell the chief, chief calls for baraza, then CDI and the community were informed.

Respondent:8 In mosque imam announces that there is this disease.

Respondent 5: You examine family of those who were affected, household members and those who have had close contact with him.

Respondent: 7 You know that’s after someone is affected but she wants to know how you heard about the disease. You knew after one is affected.

Enumerator: what about livestock? Where did you hear from?

Respondent 6: That person was affected then they start tracing it. *(Chorus and murmuring)* what causes it? They traced it and examined the livestock.

Respondent:4 vet officers

Respondent: 3 They examined the livestock which they were milking and later treated the affected people.

Enumerator: our fifth question, how do you treat those who are affected by Rift Valley Fever? R6

Respondent 6: those who are affected we treat them by…you asked about treatment method?

Enumerator: Yes.

Respondent: 4 we take them to hospital.

Enumerator: hospital.

Respondent: all yes.

Enumerator: is there other way?

Respondent: 1no.

*(Chorus)* you buy medicine.

Enumerator: you go to government hospital or private hospital?

Respondent: 2 (*Chorus response)* government hospital.

Enumerator: is there any other way you treat the affected person?

*(Chorus responses)*

R5.

Respondent 5: As pastoralists we don’t usually go to hospital but we use traditional medicine.

Enumerator: tell me about traditional medicine. Which are these medicines?

Respondent: 5 for treatment?

Enumerator: yes.

Respondent: 7 there is *Bires,* for treating fever.

Enumerator: *Bires.* How do you use it?

Respondent: 8 we distill and drink it.

Enumerator: who distill it? Are you distilling it or?

Respondent: 5 there is a traditional healer.

Enumerator: your traditional healer does it for you. Is there other traditional medicine?

Respondent: 3 there are so many trees for such treatment.

Enumerator: tell me if you know the names.

Respondent: *Burquqe*

Enumerator: *Burquqe* how do you use it?

Respondent: 2 it’s just a bark of a tree. (*Chorus responses)*

You distill it.

Enumerator: is there other tree?

(*Inaudible responses)*

Respondent: 4 those two are for treating fever.

(*Chorus responses) Walthena,*

Enumerator: how do you use it?

Respondent: We distill it and also use in tea.

Enumerator: Apart from treatment what other measures do you use as community to prevent the spread of Rift Valley Fever?

Respondent: 5 Apart from hospital?

Enumerator: yes, what are other ways to prevent the spread of this disease?

Respondent: 6 when livestock is affected it’s not slaughtered at the butcheries.

Enumerator: yes.

Respondent: 7 when they die we burn the carcass.

Enumerator: burning the carcass. Say your names.

Respondent: 1 we boil the milk

Enumerator: as R5 what do you have to say?

Respondent 5: we burn the carcass, bones and everything.

Respondent 6: we bury the carcass. also separating the affected livestock from healthy ones.

Enumerator: Any other?

Respondent 2: you inform each other that there is affected livestock.

Enumerator: informing each other. You have told me that you help livestock in giving births. How can you prevent from spreading?

Respondents: 8 *(Chorus responses)* You wear gumboots to prevent blood from spilling to your body, wearing overall,

Enumerator: what about hands?

Respondents: 2 wear gloves to prevent contact.

Respondent: 3We also vaccinate livestock.

Enumerator: I want you to tell me the effective method that helps you, you have said that you don’t slaughter livestock when there is disease, boiling milk, burning the carcass, burying the carcass, isolating the affected livestock and other is vaccination. Which is the effective method that helps you?

Respondent:5 vaccination

Enumerator: vaccination, what next?

*(Inaudible response)* R5.

Respondent 5: when livestock is affected, we don’t consume meat and milk.

Enumerator: boiling milk and meat. What next?

Respondent: 4 (*Chorus and Murmuring).* Isolating the affected livestock.

Enumerator: why isolation?

Respondent: 6 *(Chorus responses)* to prevent the spread of the disease.

Enumerator: what next? Beside vaccination and isolation of livestock.

Respondent: 5 burying the carcass.

Enumerator: why burying the carcass?

Respondent: 3 to prevent the spread because livestock wants to smell and lick the carcasses.

Enumerator: what next?

Respondent: 2 creating awareness.

Enumerator: awareness. Why is awareness important?

Respondent: 8 so as to inform them on how to prevent spread of disease.

Enumerator: I will that question later but as community which methods do you use to prevent the spread of this disease.

*(Chorus responses)*

Respondent: 4 Boiling milk.

Enumerator: why do you boil milk?

Respondent: 6 to prevent the spread to human beings.

Enumerator: we are now in section B, and we will continue to our next question. I will narrate a short story about livestock and ask one or two questions. (*Coughing and clearing throats)* I will give you the cards. We will narrate our story with these cards. Does everyone have three cards?

Respondent: All mmmmh (*All agrees).*

Enumerator: there are two people. How many people?

Respondent: two.

Enumerator: husband and wife. The husband is called Boru, and the wife is Amina. These two are married, right? They own livestock, they are pastoralists. They own cows, camels, sheep, and goats. In 2023, there was a disease in their community which affects both livestock and human beings. I want you to tell me how the ownership of Amina and Boru will influence if their livestock is affected. We don’t talk with mouths but with cards. That’s one. They own the livestock because they are husband and wife. Does Amina have control or power to sell the livestock? Are we together? They both own the livestock and there is a disease in their community that affects human beings and livestock. I will repeat for R1.

Respondent: repeat.

Enumerator: R1 we have said, there are two people, and they are called Boru and Amina who are husband and wife. They are pastoralists. Leave the cards alone (*Phone ringing)* they own cow, camels, goats, and sheep. There is an outbreak of disease, which affects both livestock and human beings. Now am asking Amina and Boru ownership, how will it influence to prevent the spread of diseases? Does Amina have control to sell the livestock (*Phone continues ringing).*

Respondent: (*Inaudible conversation)*

Enumerator: we talk with cards. This is the husband, Boru. Wife is the Amina, and the other card has both Boru and Amina. Have you understood baba?

Respondent: no. The red one is Amina?

Enumerator: yes, Yellow is Boru, green both and pink Amina

Enumerator: hold your cards the three ones. Show me this one. If I ask you the questions it’s the one who makes the decision, you just show me that one or this white one. Have you understood?

Respondent: mmmm

Enumerator: does Amina have the control to take the livestock to market and sell? As a woman does she have the control to sell? If she has the control, you raise hers if it’s Boru, you show me Boru, if both you show both. Does she have control? I have raised mine. Everyone raises one. Don’t look at each other. Put these two down.

Scores

Boru 2

Both 6

Amina 0

Reasons for Boru

Respondent:1 he is the husband he has control.

Respondent:6 The reason why he has control is the owner of the livestock. Women are like a neck and not the head. So, because are just the necks, they cannot make any decision. with his permission, she cannot sell the livestock.

Reasons for both

Respondent 4: when they want to sell the livestock, they discuss and then sell.

Enumerator: any other reason?

Respondent: 3 They discuss together and then make the decision to sale the livestock.

Respondent: 5 because they are husband and wife. So to keep the peace, they have to discuss and agree

Respondent 2: they are husband and wife, so they make decision together. But also, both of them are involved in livestock management. Joint decisions are made to avoid misunderstandings in the family.

Respondent: 1 I can solve in a way that I will tell her that I had an emergency and I can’t wait for your decision.

Respondent 5: *(Clearing throat*) When ere is disagreement, before going to government side there are elders in place that we talk to like chiefs and family of both parties.

Enumerator: Does Amina have the control to go to hospital? Does she have the control of selling the livestock and go to hospital?

Respondent:1 repeat what you have said.

Enumerator: Amina; it’s just the same story Amina wants to go to hospital, maybe she wants to take the child to the hospital

Scores

Boru 2

Both 6

Amina 0

Reasons for boru

R8: the man is the head and he makes decisions on whatever happens in the home. The woman should inform the husband about her sick or that of a child. Here the man will allow her to go to the hospital or not, because we also have options of involving elders in case of disagreements.

Respondent:1 Until Boru decides to sell of the livestock. If Boru is not around she cannot sell the livestock and go to hospital.

Reasons for Both

R2: Consultation is done to identify which animals should be sold to solve a problem.

Respondent: 3 if both of them are around they can discuss that she wants to go to the hospital and if he agrees she can ask him to take her to hospital.

R4: in case a man is not home, the discussion helps the woman to make a decision even if the husband is not home.

Respondent:6 all households’ responsibilities are under the control of the man. Even if she owns livestock, she must inform him. If he became unjust to her, she can take ‘*MEHER’* and sell. The reason God didn’t make men and women is that he is the household head and a, leader.

Respondent 1: the reason is if the wife becomes sick, the husband is aware of the sickness. If there is the understanding between them, he must be aware of wife’s sickness. So, they agree and sell one livestock and go to hospital. One remains behind with the children and other go to hospital. If you don’t do this, someone might even die.

We have seen many widows who know nothing about livestock since it was the husband who used to do the husbandry roles. Someone else takes care of her livestock and he becomes unjust and sells of the livestock but if she is with her husband, she gets to know everything in case the husband dies.

Respondent 2: they are husband and wife. If they discuss and decide to sell the livestock maybe their children want to go to the hospital.

Respondent 5: they discuss and agree to go to hospital.

Respondent 4: they discuss and since he knows his wife’s problem they decide to sell one livestock and go to hospital.

Enumerator: return your cards. Does Amina have the control when she wants to exchange the livestock? Does Amina have the power to exchange the livestock?

Enumerator: we use the cards.

Scores

Amina 1

Boru 2

Both 5

Reasons for Amina

R5: If the woman has livestock and she come to her husband house owning animals she can sell them to venture into business

Reasons for Boru

R6: Boru is the head of the house and he makes decisions because animals and his wife belongs to his irrespective of the ownership. All the resources in the home belongs to the man

Reasons for both

Respondent 7: they both the livestock, there is livestock for her hand in marriage. They can discuss but she won’t agree she can exchange the livestock.

Respondent: 3 Boru is the household head but if she sees that the livestock is good she can be able to exchange.

Respondent 6: Boru is in control because even if she owns livestock that livestock belongs to him also. God created man to be the head, so he is the shoulder to her. She has to inform Boru before any decisions are made

Respondent 5: even if Boru is the owner, it is important to have joint decisions because Amina also contributes to the rearing of the livestock and the welfare of the family.

Enumerator: R4.

Respondent 4: they discuss together which to exchange.

Enumerator: Does Amina have the control, to sell all the livestock and open up a new business, to avoid loss from the disease outbreak?

scores

Boru 3

Both 5

Amina 0

Reasons for boru

Respondent 7: the reason she doesn’t have control is that Boru is the household head. According to culture, your wife cannot go outside without your permission.

Respondent:2 Islamic culture do not allow the woman to do anything on her own or if the husband has not given her permission.

Respondent 6: the reason she isn’t in control is just the same as the previous one. According to Islamic Sharia going outside is not permissible, she must inform him of everything.

Enumerator: R8, why is he in control?

Respondent 8: if he agrees she can go. he must agree for her to go.

Reasons for both

Respondent: 1 she has her own money, and he owns his, so they discuss what to do with the money.

Respondent 2: they sell the livestock and kept the money together. When doing business, they will together and discuss what to do.

Respondent: 3 she has the right to do business if she will not use it in a bad way.

Enumerator: I will tell you another story let’s listen carefully. (*Clearing throat)* there are two people. we are almost done.

Participation

Enumerator: there are two people. They are husband and wife, the husband is called Adan, and Adan is 45 years old. They have been married for three years. For how long? Mutaa village, just like any other village. They are pastoralists; they own cows, camels, goats, and sheep. For the last few years, there is a disease which keeps occurring, it affects both livestock and human beings and in the last four years, it keeps occurring. Sharifah was invited to a seminar so that she keeps knowledge about the disease and also to create awareness. Does Sharifah have the power to attend the seminar? We use the cards to talk. Are we together? Sharifah and Adan there is a disease in their community, that disease its keeps occurring after every four years, they own cows, camels, and sheep. She was invited for a seminar. Does Sharifah have the power to attend the seminar? Show me your cards. Are we together baba?

Respondent: I have understood.

Scores

Both 5

Adan 3

Sharifah 0

Reasons for Adan

Respondent 8: Adan has the power to decide for Sharifah to attend the seminar because he is the house head

Respondent 5: the seminar is going out. Women those who have husbands, their husbands should accompany them. She cannot go out alone. So if the seminar is in Isiolo, she spends the night in the lodging alone. *(Laughing).* This is not acceptable

Respondent 6: According to Islamic culture he needs to give her permission. Where is she going? All this needs to be known so he is the head

Reasons for Both

Respondent 4: They discuss and agree. they discuss and agree if she can go alone.

R3 they are aware of each other; they inform him of the seminar and he let her go after the agreement.

Enumerator: R2.

Respondent 2: She cannot go with having discussion.

Enumerator: why can’t she make decision alone?

Respondent 2: he is the household head so they will have a discussion.

Respondent:1 there is this disease which keeps occurring; he knows why she is going. They are always together and they discuss together, there is trust between them so he let her go. She will get the knowledge abo the disease and tells him.

Respondent: my sister can I go and pick something from that probox?

Enumerator: okay. Do women attend the seminar or its men?

Respondent: *(Chorus response)* they go.

Enumerator: R1.

Respondent: they go.

Enumerator: they are no denied?

Respondent: yes.

Enumerator: what about men?

Respondent: *(Chorus)* they go.

Enumerator: men also go.

Respondent: nowadays there is gender balance, they have to go. Without women that meeting won’t happen.

(*Chorus conversation)*

Cash transfer it’s sent to their phone *(laughing)*

They go everywhere.

Enumerator: but religion doesn’t allow?

Respondent: *(chorus response)* it allows if they can go together.
