# Supplementary material for: “Without a man’s decision, nothing works”: Building resilience to Rift Valley fever in pastoralist communities in Isiolo Kenya
Source: PLoS One. 2025 Jan 28;20(1):e0316015. doi: 10.1371/journal.pone.0316015 (PMC11774392; doi:10.1371/journal.pone.0316015)
Supplement: Attachment — Submitted filename: Manuscript-Irene.docx [file pone.0316015.s002.docx]

**Title**: Without a man’s Decision, Nothing Works: Building Resilience to Rift Valley fever in Pastoralist Communities in Isiolo Kenya

**Irene N Mutambo^a,b^, Bernard Bett^b^ and Salome A. Bukachi^a^**

^a^Institute of Anthropology, Gender and African Studies, University of Nairobi, P.O. Box 30197-00100, Nairobi, Kenya

^b^One Health Research, Education and Outreach Centre in Africa, International Livestock Research Institute, P.O. Box 30709, 00100, Nairobi, Kenya

*Corresponding author e-mail: [mutamboirene@yahoo.com](mailto:mutamboirene@yahoo.com)

**Abstract**

This article presents the results of a study that sought to understand how ownership and decision-making in pastoralist male and female-headed households influence coping mechanisms and resilience to Rift Valley Fever (RVF), using the three resilience capacities absorptive, adaptive, and transformative. The study was conducted in two sub-counties (Sericho and Merti) of Isiolo County, Kenya. Data were collected through 16 focus group discussions and 13 key informant interviews with pastoralists and animal and human health stakeholders respectively. The findings indicate that traditionally, men are considered to have the final say on decisions related to livestock ownership and household overall decisions. We established that pastoralists use different approaches to reduce the effects of RVF on their livelihoods and ensure continuity. Men and women adopt coping mechanisms such as hygiene practices and mosquito nets but also adapt to proactive measures such as community knowledge dissemination, new businesses, healthcare, and indigenous medicines to have some control over their lives and that of their livestock. They also link up with community disease surveillance initiatives to strengthen disease surveillance networks and get access to county government support. This process fosters resilience, community empowerment, and transformative and sustainable adaptation responses to RVF.

**Keywords:** Decision making; gender equality; resilience; Rift Valley fever; pastoralist communities

## **Introduction**

**Rift Valley Fever**

Rift Valley fever (RVF) is a viral zoonotic disease that can be transmitted from animals to humans (1,2)by infected *Aedes* and *Culex* mosquitoes. Outbreaks of the disease usually occur after heavy rainfall and it usually affects sheep, goats, cattle, buffaloes, and camels which are primary reservoirs for human infection (3–5). The disease mostly affects the pastoralist population that depends on livestock as the main source of food and livelihood (5–7). According to (8), humans get exposed to RVF either through direct or indirect exposure pathways. RVF can be contracted through direct exposure to infected animals or their products. This includes consuming raw or undercooked animal products (e.g., milk, blood, and meat), handling infected animal bodily fluids, and inhaling aerosols from infected environments (9,10). However, indirect exposure occurs when people come into contact with infected vectors (11,12). Rift Valley fever is among the top zoonoses that are life-threatening to pastoralists and their livestock thus affecting their ability to meet basic needs and capacities to cope and respond that come with the outbreaks (5,13). In recent decades, Kenya has recorded numerous RVF outbreaks, the most recently in 1999 -1998 and 2006/7 (14,15). The outbreaks mainly affected the north and northeastern parts of the country including Garissa, Baringo, and Ijara (16).

**Household gender relations and resilience building among pastoralists.**

A focus on decision-making power dynamics in male and female headed households from a gender perspective present an important opportunity to understand the different gender roles and relations regarding adaptation strategies and innovations (19,20). For instance, men are mainly the primary final decision-makers on aspects such as the sale of livestock, access to health, household expenditures, and access to information/participation, while women are involved in making decisions around household chores (18). These variations in decision making can lead to differences in appropriate coping mechanisms and response to the effects of RVF in pastoralist communities. Some studies on adaptation indicate that joint decisions in households increase individual/household capacities to absorb and adapt to the challenges caused by natural calamities (19) such as RVF hence may have positive impacts if men and women pastoralist stop working in silos.

Resilience refers to the capacities men and women rely on to respond to and cope with challenges and shocks and prepare for future shocks (20). Resilience has been studied using different frameworks but for purposes of this study, it was assessed from the socio-ecological perspective (21). The social-ecological resilience evolves on three capacities—absorptive, adaptive, and transformative—that people employ to recover from shocks and stresses within their social-ecological systems (21). Absorptive capacity are the immediate measures and strategies pastoralist men and women use to withstand shocks for examples RVF effects without losing essential functions but rather minimize exposure to hazards and build buffers to absorb the effects brought by RVF. Examples absorptive capacity for a flooding community might have flood-resistant housing, early warning systems for natural disasters to reduce effects caused by the shocks (23). The adaptive capacity refers to the ability of pastoralists to learn from experiences, adjust their behaviours and adapt new strategies and innovations to manage future shocks when they occur (20). Example of adaptive capacities adapting to new technologies that enhance livelihood and alternative responses to changing conditions. Lastly, transformative capacity refers to the ability to create a structural atmosphere to change the barriers that hinder people to move beyond vulnerability thresholds (22) Examples included capacity to advocate for policy changes for equitable access to resources. .

In this study, we use socio-ecological resilience to analyse the different capacities that male and female pastoralists employ during RVF outbreak and how decision making within the household influence individual/household resilience to RVF. Pastoralist communities are based on patriarchal systems that often give men decision-making power over women (24). (24)posit that in such communities, authority is more likely to be in the hands of men than women based on the dos and don’ts that exclude women from making important decisions. As a result, women tend to have limited power to own resources including livestock as an adaptive capacity that can help them to negotiate for fall-back as compared to men who have ownership rights (25). In instances where women own livestock, studies have indicated that women own a small number of livestock and those that have less value as compared to large and high-value livestock (18,26).

Similarly, although men and women are familiar with and knowledgeable about the diseases that affect livestock in their communities, interventions that target prevention of such diseases primarily target men, thus creating differences in knowledge on disease prevention measures and access to animal health care among women (25,27). In addition, (28) argue that pastoralist men and women have poor knowledge about zoonotic diseases, the transmission pathways, and limited knowledge of prevention measures yet human behaviours play a crucial role in the transmission partners. As a result, pastoralist men and women face difficulties in accessing effective strategies to control zoonotic diseases such as RVF—and this in turn challenge pastoralist’s adaptive capacities to move beyond the vulnerability thresholds (26). Importantly, because pastoralists are in rural areas, they also face a challenge of poor infrastructure such as roads, access to veterinary services, well-stocked hospitals, and timely surveillance, which further exacerbate pastoralists capacities for resilience (26).

There are several studies on RVF, mostly focusing on knowledge, attitudes and practices, and perceptions and some are quantitative in nature (6). In addition, studies on resilience in pastoral areas have focused on the impact of climate on food security (29). There are also a few studies on adaptation to RVF but not focusing on household decision-making power. A qualitative study on how household decision-making and ownership influences resilience and gendered strategies adapted during RVF outbreak is able to provide information on how the gender dynamics shape and influence men’s and women’s capacities that bond negotiation for resilience during RVF outbreaks.

The objective of this study was to understand how gender relations influence resilience of pastoralist men and women to RVF in Isiolo County, Kenya. The study was guided by two research questions: i) How do men's and women's livestock ownership and decision-making power within the household influence resilience to RVF? and ii) What are the adaptation strategies used by men and women to respond to RVF and who makes decisions regarding the strategies? Our qualitative analysis focuses on how household ownership, decision making influence men’s and women’s resilience to RVF, and the gendered adaptation strategies used to respond to adverse impact of RVF outbreak.

## **Materials and methods**

**Ethical statement**

**Ethical approvals and research permits** were obtained from the relevant authorities in Kenya: the International Livestock Research Institute ethics committee (ILRI-IREC2022-56); the National Council of Science, Technology, and Innovations (NACOSTI), Kenya (NACOSTI/P/23/2313). Others include approval from the Isiolo County Department of Veterinary and local area chiefs. **Informed consent** was obtained from all participants, either through a signature or a thumb print. The participants were informed of the **study's objective**, their **free will to participate**, **confidentiality**, **anonymity**, **benefits**, and **risks.**

**Study area**

The study took place in Sericho and Merti sub-counties, Isiolo County, Kenya. Rift Valley fever has majorly affected the north and northeastern parts of the Kenya (30). The fact that RVF outbreaks has been reported in the region since 1998/9 enables this study to collect data on how household gender relations influence resilience to RVF in pastoralist communities. This study employed a cross-section research design using Focus group discussions and key informant interviews. The study used simple random sampling method for equal representation of study population. The sample size was guided by the theory of saturation (31).

**Study design and data collection**

Data collection was conducted between 26^th^ March 2023 to 16^th^ April 2023. We used vignette to understand the decision-making process within households. Participants were given three coloured cards to indicate who made each decision: orange for decisions made by men, red for decisions made by women, and green for joint decisions. Household decisions on the livestock market, access to health, household expenditures, and participation or access to information were discussed.

Participants used the cards to show how decisions are made in their households during the discussion. The researchers asked participants to raise their cards at the same time to minimize bias. After each decision was made, the number of cards for each colour was recorded, and participants were asked to elaborate on their choices. The same activity was repeated after participants discussed the management strategies; they use to respond to RVF outbreaks. Notes were taken, and audio recordings were made to capture information during the discussions. In total 116 people were interviewed from the sixteen FGDs which comprised 48 men and 68 women. While the key informants were 13 in total and were all men. **Table I** indicates the composition of participants by gender. Most participants did not have formal education and all participants were Muslims. The key informants were all men and were classified at the local or county level. They included Community Disease Reporters (CDRs), Community Chiefs, Health in charge, Disease surveillance officers, environmental officers, RVF survivors, veterinary officers, and gender officers.

**Data management and analysis**

After data collection**,** recorded data was transcribed verbatim from Borana to English by native Borana speakers. The research team reviewed all transcripts to clean them up and identify codes. Data was then coded into NVivo 14 to identify themes and content analysis was conducted to relate the findings to the study objective.

**Table 1: Number of participants in the study per category**

| Collection methods | Category | Number  of groups | Number  of participants |
| --- | --- | --- | --- |
| Focus Group Discussions | Women from male-headed households | 3 | 23 |
|  | Women from female-headed households | 3 | 23 |
|  | Female herders | 3 | 22 |
|  | Men from male-headed households | 3 | 24 |
|  | Male herders | 3 | 24 |
|  | Total | - | 116 |
| Key informants | County officials | - | 07 |
|  | Local officials | - | 06 |
| Total number of study participants |  |  | 129 |

# **Results**

**Livestock Ownership and Control**

The study participants reported they own various livestock including cows, sheep, goats, chickens, camels, and donkeys. However, certain livestock were traditionally associated with different genders (males and females). Men are the primary owners of livestock such as cows, goats, sheep, and camels; and the ownership is associated with cultural norms and traditions (e.g., inheritance rights and household heads). They inherit the livestock from their parents, grandparents, and uncles but a few bought some livestock themselves. Importantly, there were no differences in the findings between the two study locations (Sericho and Merti).

Women's ownership of livestock is primarily limited to donkeys, chickens, and to a lesser extent, goats and cows. This is attributed to a lack of inheritance rights and the practice of giving livestock to women as a token of appreciation for their labour. Women typically acquire livestock through marriage, either as a dowry from their parents or from their husbands (e.g., donkeys) to assist with household tasks (including to fetch water and firewood). However, there is a challenge in maintaining ownership of livestock, particularly those given as dowry. Men often claim ownership of these animals after marriage, leaving women with little decision-making power and requiring them to seek approval from their husbands. In cases of livestock loss due to predation, illness, or death, men may take livestock belonging to their wives to compensate for their own losses. This is illustrated in this narration:

*“Women own livestock through dowry but men sell women’s livestock without their knowledge. In addition, if men’s livestock are taken away by bandits, or men come back home and take women’s livestock. Men also take women’s livestock if their animals fall sick and die or when the animals get lost during grazing for replacement” (Female herders FGD 02).*

## **Absorptive resilience strategies and decision making**

Absorptive capacity is the ability to withstand shocks and stresses without losing essential functions (32) . It involves measures that minimize exposure to hazards and build buffers to absorb impacts of shock (21). It was established that during RVF outbreak, pastoralists use mosquito nets and repellents and hygiene practices as absorptive strategies to reduce and prevent extreme effects of RVF as detailed below.

The study participants reported that at the initial stages of RVF outbreak, pastoralists use mosquito nets and repellents as on one the immediate adaptation strategies to reduce the effects of RVF. This is important because mosquitoes are the main carriers of RVF in both animals and humans especially after the heavy rains. Accordingly, using mosquito nets and repellents helps to protect themselves from mosquito bites and reduce the risk of RVF infection. However, the decision to use mosquito nets and repellents within the household was decided by the woman (wife), while men (husbands) decided on the purchase of mosquito nets and repellents, as revealed by men and women in all the focus group discussions. In addition, men make decisions to spray the Boma^[1]^ to kill mosquitoes. The reasons why the men make the decision to purchase mosquito nets, repellent, and pesticides are; men own resources, are heads of households, and are viewed as presidents within the household. This is consistent with the male’s traditional responsibility as a breadwinner and provider.

*“We sleep under mosquito nets and repellents to avoid mosquito bites. Men are response for the purchase of mosquitos and repellents while women ensure that every member sleeps in a net” (Men from male-headed household FGD 01)*

Additionally, implementing specific hygiene practices is crucial for men and women to effectively respond and protect themselves during RVF outbreak. These practices include milking, boiling milk, and meat, clearing bushes, and setting fire/smoking and use gloves to support during birth. The study participants reported that during an RVF outbreak, members of each household must collaborate to implement hygiene practices to minimize the risk of exposure to the virus. These practices are widely recognized as effective preventive measures due to the community's awareness of their importance for maintaining health and well-being. In both men and women groups, it was noted that among married couples, decisions regarding tasks like milking, tending to fires, and clearing bushes are made jointly, with the tasks being carried based on whose what in a household. Meanwhile, women take the lead in deciding and carrying out tasks like boiling milk and meat, as well as providing home-based care. The women reported that the men also provide essential support by collecting firewood for boiling milk, and meat and lighting the fire around the compound to chase away mosquitoes. Also, when slaughtering an animal suspected of having RVF, both men and women collectively decide to discard the soup, believing it to harbour the virus and pose a risk of RVF exposure.

## **Adaptive Coping Strategies to RVF and decision making.**

Pastoralist men and women in the region have developed various strategies to cope with RVF outbreaks and protect their primary livelihood, livestock. These strategies include selling livestock, starting new businesses, and accessing healthcare services, or use of traditional herbal medicines. Selling livestock is a crucial adaptation strategy during RVF outbreaks. It provides a means of generating income and ensuring survival amidst the crisis. However, the market for livestock is limited during outbreaks due to quarantine measures implemented to control the spread of the disease. Despite these limitations, pastoralist men find ways to sell their livestock for survival. In male-headed households, men traditionally make the final decision regarding livestock sales. This decision-making authority is rooted in both customary gender roles and the desire to maintain household harmony. Men typically discuss their intentions to sell livestock with their spouses, but the final decision rests with them*: "For us not to have conflicts in our family, we sit, and I inform the women which livestock is due for selling and how many would want to sell, but I (man) make the final decision" (Men-MHH FDG02).*

On the contrary, women in male-headed households, women have more influence over poultry and household-related decisions, while men primarily control the sale of valuable livestock. In female-headed households, women assume the primary ownership and decision-making power for livestock. Their roles as caregivers and their knowledge of livestock grant them significant authority in managing and responding to RVF-related challenges. However, decision-making power in FHH is not entirely static. Factors like the presence of older sons or proximity to other family members may influence decision-making dynamics, leading to joint decisions. Despite this, women retain the final say in how funds from livestock sales are utilized, emphasizing their financial decision-making authority.

During and after Rift Valley Fever (RVF) outbreaks, venturing into new businesses is a critical coping mechanism for pastoralist communities. Women are increasingly establishing small businesses, such as vegetable stalls and general merchandise shops, within their households to secure their families' survival and meet essential needs. This shift reflects their adaptability and willingness to diversify income sources in the face of livestock losses. Decisions to start new businesses are made jointly by couples, with men providing the financial resources and women taking on the operational roles. This highlights the ongoing gender dynamics within households, where men maintain control over financial resources while women's contributions are valued for their resourcefulness and dedication to household needs.

Access to health facilities emerged as a critical adaptation strategy for coping with Rift Valley Fever (RVF) outbreaks. Participants reported seeking medical attention from government hospitals, private hospitals, or chemists when household members fell ill. Government hospitals, equipped with laboratories for blood screening and appropriate healthcare support, were often the preferred choice for patients exhibiting RVF symptoms. However, women and children within married households often faced the requirement of seeking permission from male household heads, typically husbands or fathers, before accessing medical care. This restriction stemmed from the male household head’s position as the primary resource holder and decision-maker within the household. As one female participant noted, "*Without a man's decision, nothing works, whatever the man says is right. So, he decides when to take the sick person to the hospital*" (Female herders FGD01). This highlights the deeply entrenched gender disparities influenced by traditional cultural and religious norms, which can hinder women's resilience to RVF due to limited resources and decision-making autonomy. Exceptions to this pattern do exist in emergency situations or when men are unavailable, allowing women to make independent healthcare decisions, although they are expected to inform their husbands afterward. In instances were pastoralist men and women fail to access health care, they rely on herbal medicines which are perceived effective and readily available. However, while community members express confidence in herbal medicines, health officials remain cautious, advising against their use due to concerns regarding their efficacy and safety. *“We as medical officers, we discourage the use herbals or traditional medicines to treat RVF, because we don’t know the how it works” (KII human health officials).*

## **Transformative resilience to RVF and gendered decision making.**

Transformative capacity is the ability to fundamentally alter the underlying causes of vulnerability and create new pathways to sustainability (33). It involves systemic change, challenging root causes, and re-imagining social-ecological systems (34). In this study, pastoralist men and women reported three transformative resilience strategies: access to information, RVF surveillance and vaccination that they rely on to build resilience to RVF as elaborated.

The study revel that access to knowledge before, during, and after a Rift Valley Fever (RVF) outbreak is crucial for effective response and prevention. Both men and women in pastoralist communities actively share information about RVF signs and symptoms and prevention strategies among themselves. Upon observing RVF signs in their herds or household members, pastoralists promptly disseminate information within their community and seek guidance from healthcare professionals and veterinarians. Livestock exhibiting RVF symptoms are isolated from healthy animals, and in some cases, healthy livestock are relocated to safer areas. Men generally assume the primary role in making these decisions.

Disease surveillance emerged as another crucial strategy for building resilience against Rift Valley Fever (RVF) outbreaks. Pastoralist men and women acknowledged the vital role of Community Health Volunteers (CHVs) and Community Disease Reporters (CDRs) in effectively educating the community about RVF transmission risks, exposure symptoms, and preventive measures. These trained community volunteers actively engage with the community to identify potential cases and promptly report them to the county government before the disease causes widespread harm. Their efforts ensure timely sample collection for laboratory analysis and swift response to RVF outbreaks.

Vaccination is considered an effective strategy for protecting livestock from Rift Valley Fever (RVF) and preventing its transmission to humans. Pastoralists recognize its ability to boost livestock immunity and provide long-lasting protection against RVF. They express a preference for vaccinating their animals during the rainy season, as mosquito breeding increases following heavy rainfall, raising the risk of RVF transmission. Men hold the final decision-making authority regarding livestock vaccinations. This stems from their ownership of the animals, their perceived greater knowledge about livestock care, and the practical challenges faced by women in traveling to distant vaccination centers and managing household responsibilities.

# **Discussion**

In this study, we used a qualitative approach to understand how gender relations influence resilience to RVF in pastoralist communities in Isiolo County, Kenya. The study found gender-based differences relating to livestock ownership, and how ownership and decision-making power influence individual resilience to RVF in pastoralist communities. This is in line with, (29) who note that men in pastoralist communities are household heads and owners of livestock. In addition unmarried women and men own and make decisions on almost everything, as a result, their level of adaptation capacity is relatively high as compared to married women who lack ownership rights and decision-making power. Similarly, men and single women have more freedom to make decisions on adaptation innovations because they are the ultimate decision makers within the household (1). Additionally, women’s ownership of livestock is limited because in instances where women own livestock, marketing decisions and use of resources are determined by the men (35).

Livestock ownership is crucial for pastoralist communities, serving as a means of generating income, accessing healthcare, covering household expenses, and participating in community activities. However, this reliance on livestock reinforces gender disparities in decision-making, as social and cultural norms grant men greater authority over livestock ownership and control. These power dynamics perpetuate gender inequalities within households, influencing decision-making regarding adaptation strategies in response to crisis such as Rift Valley Fever outbreaks. According to (36), a person’s fall-back position is determined by the resources they own. Accordingly, we argue that men have strong bargaining power and their ability to bounce back after adverse effects of RVF compared to women whose social structural hierarchy to ownership is limited. Women’s limited ownership of livestock not only affects their bargaining power of access to health and access information but also limits their ability to negotiate the appropriate adaptation strategies to RVF.

The absorptive capacities reveal the vital role of gender dynamics in the adoption of preventive measures such as the use of mosquito nets and repellents. While women are primarily responsible for the decision to use these protective measures within the household, men play a significant role in the procurement of mosquito nets and repellents, reflecting their traditional position as the providers and heads of households. This observation resonates with the findings of previous studies such as (37), emphasizing the gendered division of responsibilities within household decision-making processes related to health and hygiene practices. Moreover, the study illuminates the joint decision-making process between men and women concerning various hygiene practices, including milking, boiling milk, and meat, as well as the clearance of bushes to minimize the risk of RVF exposure. While decisions related to milking, fire management, and bush clearance are jointly determined, women take the lead in decisions involving the boiling of milk and meat and home-based care, reflecting their central role in ensuring household health and well-being. These findings align with the conclusions drawn by (28), emphasizing the collaborative nature of gendered roles in promoting health and safety within pastoralist communities. Additionally, the joint decision to discard the soup from potentially infected animals sheds light on the shared responsibility of men and women in safeguarding the health of their families. This finding corroborates the research by (38), emphasizing the joint decision-making process within households as a crucial component of risk mitigation strategies during zoonotic disease outbreaks.

In relation to adaptive capacities, the findings of our study reveal pronounced gender disparities in decision-making processes concerning the sale of livestock, reflecting the dominance of male decision-makers, which aligns with the conclusions drawn by previous studies such as (17,18) These studies emphasize the pivotal role of men in agricultural and livestock-related decision-making within similar contexts. Moreover, our research sheds light on the unique decision-making dynamics within female-headed households, highlighting the significant decision-making authority of women, which resonates with the observations made other studies (39,40). In the context of healthcare access, our study highlights the barriers faced by women due to traditional gender norms, corroborating the observations made by (41). This study underline how cultural norms restrict women's autonomy, hindering their ability to access healthcare services independently during health crises such as RVF. Additionally, the use of herbal medicine as a coping mechanism finds support in the research of (13,42,43), which highlight the significance of traditional healing practices within communities dealing with health emergencies. Furthermore, the study's exploration of business diversification reveals a joint decision-making process between men and women, aligning with the findings of (44), emphasizing the importance of joint decision-making in livelihood diversification strategies during crises.

The transformative capacities tell the critical role of knowledge sharing among community members, highlighting how both men and women actively disseminate information about RVF signs, symptoms, and prevention strategies within their social networks. This collective knowledge dissemination approach aligns with the findings of previous studies, such as (45) emphasizing the pivotal role of community-based knowledge exchange in enhancing disease awareness and response within pastoralist communities. Furthermore, the study emphasizes the transformative role of disease surveillance facilitated by Community Health Volunteers (CHVs) and Community Disease Reporters (CDRs). These community volunteers play a crucial role in sensitizing the community about RVF transmission risks, symptoms, and prevention strategies, thereby fostering proactive measures to contain the spread of the disease. The collaboration between the CHVs, CDRs, and government disease surveillance coordinators reflects a transformative approach to community engagement and empowerment, aligning with the conclusions drawn by (45) regarding the significance of community-based surveillance in disease management and response. Moreover, the study highlights the transformative potential of vaccination strategies in protecting livestock and preventing the transmission of RVF to humans. However, it identifies the unequal decision-making dynamics within households, with men primarily responsible for making vaccination decisions without actively involving their partners. This observation resonates with the findings of (46),emphasizing the need for gender-equitable decision-making processes in livestock management and disease prevention strategies within pastoralist communities.

**Conclusion**

In conclusion, to build sustainable and effective resilience against zoonotic diseases like RVF, it is imperative to integrate gender-responsive approaches into all stages of planning and implementation. Empowering women, fostering collective decision-making, and promoting inclusive participation in knowledge sharing, disease surveillance, and adaptive measures are essential for enhancing the overall resilience and well-being of pastoralist communities facing complex health challenges. By recognizing the collaborative potential of absorptive, transformative, and adaptive capacities, policymakers and stakeholders, this in turn can foster a more holistic and gender-inclusive approach to resilience-building, ensuring the long-term health and sustainability of these communities.

**Limitations**

A cross-sectional study design was used in this study to learn how ownership and household decisions influence men's and women’s capacities for resilience. The researcher did not collect data during or immediately after the RVF outbreak to interview pastoralist men and women on what adaptation strategies they use to respond to RVF and who makes the final say on the adapted strategies. All the data collected draws from previously remembered experiences. Arguably, the information gathered is helpful because it enables us to comprehend how men and women respond and reduce the adverse effects of RVF and who in the household has the final. In addition, the information is useful in understanding how livestock ownership influences pastoral men and women’s resilience capacities.

**Acknowledgements**

The authors extend their gratitude to the project team, community leaders, and members in Merti and Sericho sub-county, the Isiolo County Veterinary Department, the Public Health Department, the County Hospital, and the Gender Department. Special appreciation goes to the enumerators who worked tirelessly to ensure the smooth running of data collection.

# **References**

1.	SHIBATA R, CARDEY S, DORWARD P. GENDERED INTRA-HOUSEHOLD DECISION-MAKING DYNAMICS IN AGRICULTURAL INNOVATION PROCESSES : ASSETS , NORMS AND BARGAINING. J Int Dev [Internet]. 2020; Available from: doi: 10.1002/jid.3497%0AGENDERED

2.	Yahia S Ben, Khochtali S, Abroug N, Khairallah M. Rift valley fever. Intraocular Inflamm. 2016;1209–13.

3.	Kariuki Njenga M, Bett B. Rift Valley Fever Virus—How and Where Virus Is Maintained During Inter-epidemic Periods. Curr Clin Microbiol Reports. 2019;6(1):18–24.

4.	Hassan A, Muturi M, Mwatondo A, Omolo J, Bett B, Gikundi S, et al. Epidemiological investigation of a rift valley fever outbreak in humans and livestock in Kenya, 2018. Am J Trop Med Hyg. 2020;103(4):1649–55.

5.	Mburu CM, Bukachi SA, Bett B. Pastoralists ’ perceptions on the impact of Rift valley fever disease following an outbreak in North Eastern Kenya. Pastor Res Policy Pract. 2022;12(24).

6.	Ng’ang’a CM, Bukachi SA, Bett BK. Lay perceptions of risk factors for Rift Valley fever in a pastoral community in northeastern Kenya. BMC Public Health [Internet]. 2016;16(1):1–10. Available from: http://dx.doi.org/10.1186/s12889-016-2707-8

7.	Ibrahim M, Schelling E, Zinsstag J, Hattendorf J, Andargie E, Tschopp R. Sero-prevalence of brucellosis, q-fever and rift valley fever in humans and livestock in Somali region, Ethiopia. PLoS Negl Trop Dis [Internet]. 2021;15(1):1–18. Available from: http://dx.doi.org/10.1371/journal.pntd.0008100

8.	Bett B, Lindahl J, Sang R, Wainaina M, Kairu-Wanyoike S, Bukachi S, et al. Association between Rift Valley fever virus seroprevalences in livestock and humans and their respective intra-cluster correlation coefficients, Tana River County, Kenya. Epidemiol Infect. 2019;147.

9.	Nyakarahuka L, de St. Maurice A, Purpura L, Ervin E, Balinandi S, Tumusiime A, et al. Prevalence and risk factors of Rift Valley fever in humans and animals from Kabale district in Southwestern Uganda, 2016. PLoS Negl Trop Dis. 2018;12(5):1–16.

10.	Affognon H, Mburu P, Hassan OA, Kingori S, Ahlm C, Sang R, et al. Ethnic groups’ knowledge, attitude and practices and Rift Valley fever exposure in Isiolo County of Kenya. PLoS Negl Trop Dis. 2017 Mar;11(3):e0005405.

11.	Bett B, Said MY, Sang R, Bukachi S, Wanyoike S, Kifugo SC, et al. Effects of flood irrigation on the risk of selected zoonotic pathogens in an arid and semi-arid area in the eastern Kenya. PLoS One. 2017;12(5):e0172626.

12.	Owange NO, Ogara WO, Kasiiti J, Gathura PB, Okuthe S, Sang R, et al. Perceived risk factors and risk pathways of Rift Valley fever in cattle in Ijara district, Kenya. Onderstepoort J Vet Res. 2014 Nov;81(1).

13.	Muga. Adaptive and Coping Mechanisms of Pastoralists in Dry Land Ecosystem and Their Implications on Rift Valley Fever Control in. Int J Nov Res Humanit Soc Sci [Internet]. 2018;5(2):21–33. Available from: https://www.noveltyjournals.com/upload/paper/Adaptive and Coping Mechanisms-1320.pdf

14.	Hassan A, Muturi M, Mwatondo A, Omolo J, Bett B, Gikundi S, et al. Epidemiological Investigation of a Rift Valley Fever Outbreak in Humans and Livestock in Kenya, 2018. Am J Trop Med Hyg. 2020 Oct;103(4):1649–55.

15.	Bett B, Jost CC, Nzietchueng S, Kihu S, Njogu G, Emmanuel S. Swai and JCM. Epidemiological Assessment of the Rift Valley Fever Outbreak in Kenya and Tanzania in 2006 and 2007. Am Soc Trop Med Hyg Epidemiol [Internet]. 2010;83(Suppl 2):65–72. Available from: doi:10.4269/ajtmh.2010.09-0290

16.	Mutua EN, Bukachi SA, Bett BK, Estambale BA, Nyamongo IK. “We do not bury dead livestock like human beings”: Community behaviors and risk of Rift Valley Fever virus infection in Baringo County, Kenya. PLoS Negl Trop Dis. 2017 May;11(5):e0005582.

17.	M. Acosta et al. What does it Mean to Make a ‘ Joint ’ Decision ? Unpacking Intra-household Decision Making in Agriculture : Implications for Policy and Practice. J Dev Stud [Internet]. 2019;56(6):1210–29. Available from: https://doi.org/10.1080/00220388.2019.1650169

18.	Njuki J, S K, A C, W C. Linking Smallholder Farmers to Markets, Gender and Intra-Household Dynamics: Does the Choice of Commodity Matter? Eur J Dev Res [Internet]. 2011;23(3 426-443):426–43. Available from: doi:10.1057/ejdr.2011.8

19.	Cheryl r. Doss and Michael L. Morris. How does gender affect the adoption of agricultural innovations ? The case of improved maize technology in Ghana. Agric Econ [Internet]. 2001;25:27–39. Available from: doi/10.1111/j.1574-0862.2001.tb00233.x

20.	Steinbach D, Kaur N, Manuel C, Saigal S, Agrawal A, Panjiyar A, et al. Building resilience to climate change MGNREGS , drought and flooding in Odisha. London; 2017.

21.	Berkes & Ross. Community Resilience : Toward an Integrated Approach Community Resilience : Toward an Integrated Approach. Soc Nat Resour. 2013;26(1):5–20.

22.	Liru and Heinecken. Building Resilience : The Gendered Effect of Climate Change on Food Security and Sovereignty in Kakamega-Kenya. 2021;

23.	Cheong & Assenova. Absorptive capacity facilitates adaptation to novel environmental disasters. PLoS One. 2021;16(11):1–11.

24.	Fiona Flintan. WOMEN ’ S EMPOWERMENT IN PASTORAL SOCIETIES Fiona Flintan [Internet]. Addis Ababa; 2008. Available from: https://www.iucn.org/sites/default/files/import/downloads/gender_format.pdf

25.	Miller AB. The Gender and Social Dimensions to Livestock Keeping in Africa : Implications for Animal Health Interventions [Internet]. England; 2011. Available from: https://assets.publishing.service.gov.uk/media/57a08ac940f0b6497400078e/GALVmed-African-Gender-Report.pdf

26.	Eba B, Wieland B, Flintan F, Nelly N, Baltenweck I. Gender and One Health context analysis for HEAL. 2020;(October). Available from: https://cgspace.cgiar.org/handle/10568/111329

27.	Alessandra G, Distefano F, Kangogo D, Mattioli RC, Baltenweck BWI. Gendered Perspectives on Smallholder Cattle Production and Health Management in Three Sites in Tanzania. J Gender, Agric Food Secur. 2017;2(3):43–65.

28.	Amenu K, Wieland B, Szonyi B, Grace D. Milk handling practices and consumption behavior among Borana pastoralists in southern Ethiopia. J Health Popul Nutr. 2019;38(6):12.

29.	Bukachi Salome, Ameso EA, Olungah CO, Haller T, Wandibba S, Nangendo and S. Pastoral resilience among the maasai pastoralists of Laikipia County, Kenya. Land. 2018;7(2):1–17.

30.	Mutua E. Community Adaptation to Malaria and Rift Valley Fever in Baringo County, Kenya [Internet]. Nairobi University; 2017. Available from: http://erepository.uonbi.ac.ke/handle/11295/101371

31.	Krueger & Casey. Focus group discussion: A practical guide for applied research [Internet]. 5th ed. Helen Salmon, Anna Villarruel TB& PS, editor. United Kingdom: SAGE Publications; 2015. 345–500 p. Available from: https://us.sagepub.com/en-us/nam/book/focus-groups-6

32.	Adger WN. Social-Ecological Resilience to Coastal Disasters. Science (80- ). 2005;309(1036).

33.	Fedele et al. Transformative adaptation to climate change for sustainable social- ecological systems. Enviromental Sci Policy. 2019;101(August):116–25.

34.	Elinor Ostrom. Special section. Science (80- ) [Internet]. 2009;325(5939):419–23. Available from: 10.1126/science.1172133

35.	Njuki J, Sanginga PC. Women, livestock ownership and markets: Bridging the gender gap in eastern and Southern Africa [Internet]. 1st ed. Jemimah Njuki and Pascal C. Sanginga, editor. Women, Livestock Ownership and Markets: Bridging the gender gap in Eastern and Southern Africa. New York: Routledge; 2013. 1–148 p. Available from: https://idl-bnc-idrc.dspacedirect.org/bitstream/handle/10625/52269/IDL-52269.pdf?sequence=1

36.	Agarwal B. ’ ’ Bargaining ’ ’ and Gender Relations : Within and Beyond the Household. Vol. 5701. 2011.

37.	Wachinger J, Reñosa MDC, Endoma V, Aligato MF, Landicho-Guevarra J, Landicho J, et al. Bargaining and gendered authority: a framework to understand household decision-making about childhood vaccines in the Philippines. BMJ Glob Heal [Internet]. 2022 Sep 1;7(9):e009781. Available from: http://gh.bmj.com/content/7/9/e009781.abstract

38.	Osbjer K, Boqvist S, Sokerya S, Kannarath C, San S, Davun H, et al. Household practices related to disease transmission between animals and humans in rural Cambodia. BMC Public Health. 2015;15(476):1–10.

39.	Posel DR. Who are the heads of household , what do they do , and is the concept of headship useful ? An analysis of headship in South Africa. Dev South Afr [Internet]. 2010;18(5):1–21. Available from: https://doi.org/10.1080/03768350120097487

40.	Wondimu H, Delelegn W, Dejene K. What do female-headed households ’ livelihood strategies in Jimma city , South west Ethiopia look like from the perspective of the sustainable livelihood approach ? What do female-headed households ’ livelihood strategies in Jimma city , South west Ethiopia look like from the perspective of the sustainable livelihood approach ? Cogent Soc Sci [Internet]. 2022;8(1). Available from: https://doi.org/10.1080/23311886.2022.2075133

41.	Miller BBA. The Gender and Social Dimensions to Livestock Keeping in Africa : Implications for Animal Health Interventions Prepared for GALVmed. 2011;(March).

42.	Mangesho PE, Neselle MO, Karimuribo ED, Mlangwa JE, Queenan K, Mboera LEG, et al. Exploring local knowledge and perceptions on zoonoses among pastoralists in northern and eastern Tanzania. PLoS Negl Trop Dis. 2017;11(2):1–22.

43.	Namusisi S, Mahero M, Travis D, Pelican K, Robertson C, Mugisha L. A descriptive study of zoonotic disease risk at the human-wildlife interface in a biodiversity hot spot in South Western Uganda. PLoS Negl Trop Dis [Internet]. 2021;51(1):1–16. Available from: http://dx.doi.org/10.1371/journal.pntd.0008633

44.	Kassie GW, Kim S, Jr FPF, Worku G, Kim S, Jr FPF, et al. Determinant factors of livelihood diversification : Evidence from Ethiopia. Cogent Soc Sci [Internet]. 2017;26. Available from: https://doi.org/10.1080/23311886.2017.1369490

45.	Gammino VM, Diaz MR, Id SWP. Health services uptake among nomadic pastoralist populations in Africa : A systematic review of the literature. 2020;1–23. Available from: http://dx.doi.org/10.1371/journal.pntd.0008474

46.	Mutua E, De Haan N, Tumusiime D, Jost C, Bett B. A qualitative study on gendered barriers to livestock vaccine uptake in kenya and uganda and their implications on rift valley fever control. Vaccines. 2019;7(3):1–22.
