# Supplementary material for: “Without a man’s decision, nothing works”: Building resilience to Rift Valley fever in pastoralist communities in Isiolo Kenya
Source: PLoS One. 2025 Jan 28;20(1):e0316015. doi: 10.1371/journal.pone.0316015 (PMC11774392; doi:10.1371/journal.pone.0316015)
Supplement: Attachment — Submitted filename: Response to Reviewers.docx [file pone.0316015.s003.docx]

**Rebuttal Letter**

Dear Editor,

Thank you for the opportunity to revise and resubmit our manuscript, "”Without a Man’s Decision, Nothing Works”: Building Resilience to Rift Valley fever in Pastoralist Communities in Isiolo Kenya" [PONE-D-24-03443]. We appreciate the time and valuable feedback provided by the reviewers and editorial team. We have carefully addressed each concern, and we believe these revisions have significantly strengthened the manuscript.

Enclosed please find the following:

- **Response to Reviewers:** A point-by-point response to each comment, outlining the specific changes made in the manuscript.

We are confident that our revised manuscript now meets the high standards of PLOS One. We are grateful for your consideration and look forward to your decision.

Sincerely,

Nabwire Irene Mutambo

Response to the reviewers’ comments

| **Section** | **Comment line** | **Comment** | **Response line** | **Page number** | **Response to the comments** |
| --- | --- | --- | --- | --- | --- |
| General comments |  | Do not use in text citations in the place of the authors name you are mentioning |  |  | We have adopted the PLOS ONE referencing style |
|  |  | All figures need a title and should be labeled Figure 1, Table 1, ect. The figure should come immediately after the paragraph it is mentioned in |  |  | All the figures/titles have been given titles and moved to the appropriate places |
| Reviewer #1 |  |  |  |  |  |
| Title | 2 | the first part of the title is a participant quote, add quotation marks. | 2 | 1 | Yes, the quote was picked from the participant’s response. The quotation has been added (though the journal system doesn’t allow quotes while submitting and that is how I submitted the title without the quotes with the advice from the PLOS ONE team) |
| **Abstract** | 11 | Begin the abstract with a brief introduction of RVF and/or a summary of what is known about gendered differences in pastoral resilience. Move “this article presents…” to the end of the abstract | 11-13 | 1 | The abstract now includes an introduction to RVF and highlights key gendered differences in resilience, offering a concise overview of our findings |
|  | 18 | “We established that pastoralists use different approaches to reduce the effects of RVF on their livelihoods and ensure continuity” Pastoralist use different approached than who? Or men and women use different approached? This sentence is vague, and it would be best to re-word. Has the study established this or was it already known? | 21-23 | 2-3 | This sentence has been revised to specify the different approaches used by men and women pastoralists in coping with RVF, enhancing clarity. This is a study finding |
| Introduction | 31 | Change can be to “is” and you may also want to note that RVF can be directly transmitted to humans from animals | 36 | 2 | The adjective “is” has been adopted as requested and the statement has been revised |
|  | 33 | Best to avoid referring to RVFV as "it". You could change “and it usually” to “impacting” | 38 | 2 | Referring has been removed from the sentence |
|  | 35 | Instead of (8), write the authors name if this is an article “Name and others.” However, this is basic RVF knowledge, and you can just write the sentence and the citation at the end | 48 | 3 | This is a referencing/citation style for PLOS ONE. The citation has been put at the end of the sentence |
|  | 39 | Remove “However” | 48 | 3 | “However,” has been removed |
|  |  |  |  |  |  |
|  | 43 | I disagree that the most recent outbreak was in 2007. See 2018, 2020, now…? | 55 | 3 | We have updated this section with details of subsequent outbreaks in 2018, 2020, and 2024, providing a more complete historical context. |
|  | 44 | I also disagree with this statement that the 2007 outbreak mainly affected the NE. | 58 | 3 | This statement has been reworded and the 2020 outbreak that affected Isiolo county has been captured |
|  | 46 | Remove headings from the introduction. | 60 | 3 | The heading has been removed |
|  | 47 | Indent new paragraph | 61 | 3 | The new paragraph has been indented after the heading has been removed |
|  | 49 | Remove “are mainly” if they are primary. This is repetitive | 63 |  | “Mainly” has been removed” |
|  | 52 | I am not an expert in women decision making, but I believe it is known that women are involved heavily in decisions around child health and nutrition. You may mention this to highlight that women make decisions beyond household chores | 66 | 3 | Children’s health and nutrition has been introduced as suggested by the reviewer |
|  | 54 | Adaptation to what? | 72 | 4 | The sentence has been revised for clarity |
|  | 56 | start new sentence with “Hence, | 71-80 | 4 | This whole sentence has been revised |
|  | 60 | Remove mentions of your study’s method from the introduction. I would start this paragraph at line 61 rather than with the definition of resilience | 84 | 4 | The method has been removed and the definition of resilience. |
|  | 61 | Add the word “framework,” “theory” or something similar after “social-ecological resilience” | 93 | 5 | The word “theory” has been introduced after “social-ecological resilience” |
|  | 64 | New sentence “For example,” | 98 | 5 | A new sentence has been introduced with “For example” |
|  | 66 | Add “of between examples and abortive and replace “might have” with “include” since you are giving an example. | 98 | 5 | This sentence has been revised to address all the grammatical errors |
|  | 73 | It becomes repetitive to read “examples” you can shorten this by giving a simple example in the preceding sentence by using “such as” | 100-106 | 5 | The sentence has been shortened and conjunctions (for instance, such as” have been used to replace “example” |
|  | 75 | Again, this is the introduction, remove all mentions of your study methods. | 107-116 | 5-6 | Methods have been removed from the introduction |
|  | 78 | This section needs to be in the same paragraph as when you first mention gender and womens’ roles. It interrupts the flow here. There is a typo here also | 74-80 | 4 | This paragraph has been moved and placed after the indented paragraph. In addition, this paragraph has been revised to enhance clarity |
|  | 85 | This paragraph also does not seem to fit here. Perhaps move these mentions of challenges above the part where you talk about building resilience | 81-89 | 4 | This paragraph has been moved to the third after the indented paragraph |
|  | 97 | Be more specific on the type of RVF studies because most RVF studies overall are not focused on KAP, they are epidemiological studies. This paragraph could be deleted entirely as it does not add to the background of your study. It is fine to state that your study is a good idea from line 101 onward but make it shorter and focus on the potential impact of having such data. Then, follow with the objective of this study in one line. | 129-136(deleted paragraph)  145-158(introduces a new paragraph) | 6-7 | The paragraph has been deleted as suggested by the reviewer |
|  | 106 | Your research questions do not belong in the introduction. End the introduction with the objective/intent of your research. | 137-144 | 7 | The research questions have been deleted from the manuscript and the study objective has been put at the end of the introduction |
| Methods | 113 | You could start the methods with an overview of your study moving everything from the introduction | 161-169 | 8 | An overview of the study has been provided |
|  | 120 | Confirm if you reviewed the consent form orally with those participants who were unable to read it and the language consent forms were delivered in | 409 | 15 | Yes, the consent forms were reviewed orally in the local language with all the participants regardless of whether an individual was able/unable to read. The sentence has been revised for clarity |
|  | 122 | If the key information here is that your study area is pastoralist. It would be good to give information about livestock ownership, and what proportion of households are classified as pastoral/semi-pastoral. I believe this is available from the 2019 census | 179-184 | 8 | This information has been provided |
|  | 124 | This is background information you have already provided in the intro. Delete | 185-189 | 9 | This information has been deleted |
|  | 127 | employed? | 189 | 9 | This section has been revised to enhance clarity |
|  | 121 | Data collection was “carried out” | 207-211 | 10 | The section on the “study area” was reworked and information was added for a good flow of information |
|  | 131 | “a vignette” You could combine this sentence with line 132 to improve flow | 207 | 10 | Line 131 and 132 have been combined to improve the structural flow of the argument |
|  | 137 | This figure is presented without a title and does not follow logically. It interrupts this description of the vignette you are trying to give. Figures should be placed immediately after the paragraph they are mentioned in. This looks like it belongs with study site. Furthermore, the scale you have presented the landcover at is not easy to interpret. It would be best to choose the most prominent landcover types. I don’t see any open water on the map, so it should not be in the legend | 191 | 9 | A title has been provided to the figure and the figure has been moved next to the study site. The map plus the scale have been revised |
|  | 140 | Were males and females questioned in the same space? Were any measures in place to ensure they were revealing their cards at the same time and that bias was indeed reduced? | 197 | 10 | We had separate groups for men and women and this has been well described in the current manuscript |
|  | 146 | Table 1 is also misplaced. It should be placed after this paragraph where you mention it in the | 256 | 13 | The table has been deleted |
|  | 154 | “clean them up” is too informal. State the modifications that were made | 251-255 | 13 | The use of formal language in the sentence has been adapted. |
|  | 161 | I have just realized that you have many results in the methods section. Remove all mentions of results in the methods, for example table 1. | 160-255 | 8-13 | All the results have been removed from the results section and Table 1 has been deleted because the information in the table is elaborated in the naration |
| Results | 163 | Did all study participants own all these animals? Include numbers and proportions if this is a finding. | 266-270 | 14 | Yes, all the study participants reported ownership of various livestock. This paragraph has been revised to specify the different livestock ownership by men and women and the proportion has been provided, enhancing clarity |
|  | 166/167 | Was this a finding from your data collection or is this cultural norm something that is known? If so, it belongs in the introduction where you talk about gendered differences. | 270-273 | 14 | Yes, the finding was from data collection. This has been reworked for clarity |
|  | 170 | It would be best to give numbers and percentages here as this seems highly relevant to your findings. | 270-270 | 14 | Percentages have been provided |
|  | 171 | The part where you said “this is attributed” is an interpretation of results. This belongs in the discussion | 290-298 | 15 | This statement has been removed and all interpretation statements have been removed from the results section |
|  | 172 | Describing how women typically acquire livestock is background information. This belongs in the introduction unless your study identified this as a theme. If it is the later, it would be best to put this under a heading that you are leading into the results of the thematic analysis. The same goes from like 175 onward. I am unsure if this is a result of your study or misplaced introduction/interpretation of your results. | 290-291 | 15 | This statement highlights the reasons why women own what they own but not an introduction. The sentence has been reworded to reflect the study findings |
|  | 185 | This is another example of introduction in the results. It makes it very difficult to follow what this study has found. | 304 | 16 | The information was deleted |
|  | 190 | Of what RVF outbreak? If you mean outbreaks in general, add an s to an outbreak. It would also be impactful to describe what outbreak they are referring to. Above you mention that your interpretation is the last outbreak was in 2007, if this is the one the participant is referring to, this is quite a long recall time. Depending on the above answer, you may consider including recall bias in your limitations section of the discussion. | 324 | 17 | The sentence was revised and “an” was added. The introduction was revised to capture the most recent RVF outbreaks of 2018, 2020, and 2024. However, for this study, the participants referred to the 2020 RVF outbreak that affected their community. Recall bais was one of the study limitations |
|  | 192 | Did the participants say this or is it your interpretation? If a participant said this, you can re-word “In X/X groups, participants noted that…” | 326-327 | 17 | The sentences have been revised to indicate which study participants (FGD/KII) reported the finding |
|  | 206 | Here is another example where it is hard to understand if this is a result. Results are generally in the past tense. In line 209, you then say the participants reported something, so it makes the reader understand that everything prior to that was misplaced background information. | 353 | 18 | The sentence has been reworded to capture the participant's responses/study findings. |
|  | 219 | Define what the soup is from slaughter | 364 | 18 | The definition of soup has been provided and the sentence has been revised |
|  | 225 | Another example of background text. I will stop pointing this out from this line onwards as I can’t interpret the findings of the study. | 380-394 | 19-20 | The paragraph has been revised to reflect the study findings |
|  | 297 | Did the participants say this or this this a general statement on RVF vaccination? | 531-539 | 25-26 | 531-539 Yes, the study participants reported vaccination as a coping strategy. The sentence has been revised |
| Discussion | 310/325 | Put the authors name instead of an in-text citation | Throughout the manuscript | All | The in-text citation is the preference of the journal |
|  | 398 | Limitations should be a paragraph in the discussion rather than a separate section. I’d suggest the second to the last paragraph. | 646-654 | 30-31 | The limitations of the study have been moved to the discussion section |
|  | 418 | All references need to have their format checked. The first one is all capital letters. | 874-1055 | 40-46 | All references have been checked, and formatted |
| Reviewer #2 |  |  |  |  |  |
|  | 99-100 | In line number 99-100 indicating "there are aslo a few studies......" need reference. | 129 | 6 | Reviewer#1 suggested that line numbers 97-100 should be removed. Therefore, the information that needed a reference was removed |
|  |  | Please mention future implication after the objective of the study. | 152-158 | 7 | The future implication of the study has been provided and placed after the study objective |
|  |  | Provide an ethical statement at the last portion of the study | 722-731 | 34 | The ethical statement has been moved from the method section to the last portion of the manuscript |
|  |  | Add more references in the discussion section | Throughout the introduction and discussion  Reference numbers: 12, 14,15,16,32,34,36,37,41,42,43,45,46,47,57, and 59 | Throughout the introduction and discussion sections | More references have been added |
|  |  | Add limitation in the discussion section | 646-654 | 30-31 | The limitations of the study were moved to the discussion section |
|  |  | Please check overall English language | All | All | A thorough review of the entire manuscript has been done to address the grammatical errors |
| Reviewer # 3 |  | Need to generate the latest data, focus on study design again, and sample size determination. A conclusion should be result-oriented with a focus one future implications. The discussion part can be short based on key findings | Conclusion-699-709  Simple size-and study design-194-206 | 33  9-10 | New latest references have been added. However, in instances where we could not find the latest references, we maintained what we had. The conclusion was revised to reflect the study results and implications, and further research has been provided. The discussion has been summarized to capture key findings |
