# Supplementary material for: “Without a man’s decision, nothing works”: Building resilience to Rift Valley fever in pastoralist communities in Isiolo Kenya
Source: PLoS One. 2025 Jan 28;20(1):e0316015. doi: 10.1371/journal.pone.0316015 (PMC11774392; doi:10.1371/journal.pone.0316015)
Supplement: Attachment — Submitted filename: Response to Reviewers-.docx [file pone.0316015.s004.docx]

**Rebuttal Letter**

Dear Editor,

Thank you for the opportunity to revise and resubmit our manuscript, "Without a Man’s Decision, Nothing Works”: Building Resilience to Rift Valley fever in Pastoralist Communities in Isiolo Kenya" [PONE-D-24-03443]. We appreciate the time and valuable feedback provided by the reviewers and editorial team. We have carefully addressed each concern, and we believe these revisions have significantly strengthened the manuscript.

Enclosed please find the following:

- **Response to Reviewers:** A point-by-point response to each comment, outlining the specific changes made in the manuscript.

We are confident that our revised manuscript now meets the high standards of PLOS One. We are grateful for your consideration and look forward to your decision.

Sincerely,

Nabwire Irene Mutambo

Response to the reviewers’ comments

| **Section** | **Comment line** | **Comment** | **Response line** | **Page number** | **Response to the comments** |
| --- | --- | --- | --- | --- | --- |
| Reviewer #1 |  |  |  |  |  |
| Title | 2 | Some inconsistency with capital letters. Would only capitalize: Without, Building, Rift Valley, Isiolo, and Kenya | 2 | 1 | The suggestion to capitalize Without, Building, Rift Valley, Isiolo, and Kenya has been adapted and the rest of the wording in the title bears small letters |
| Introduction | 32 | Remove the word “directly” | 32 | 2 | The word directly has been removed |
|  | 57 | What are do’s and don’ts? Is this a theory? You could consider starting this sentence with “Based on the theory of x, ___” or take this part out completely. It looks like you are simply trying to make the point that when women have control of resources, they can cope better and negotiate…? | 57-58 | 3 | The sentence has been revised and do’s and do not’s have been removed |
|  | 74 | End sentence after “shocks” to remove run on sentence | 74 | 4 | A full stop has been introduced to avoid run on sentence |
|  | 80 | End sentence after “occur” to remove run on sentence | 80 | 4 | A full stop has been introduced to avoid run on sentence |
|  | 92 | From “This study’s..” until line 98: This should be in the discussion rather than introduction. | 92-98 | 5 | The statement has not been moved because, in the first round of comments, Reviewer #2 suggested I should “mention future implications after the study's objective” in the original manuscript (lines 99-100). That is why is introduced the implication here |
| Materials and Methods | 101 | end sentence after Kenya. The explanation of RVF does not need to be repeated in methods | 99-105 | 6 | The following statement after Kenya has been removed to |
|  | 127 | Minor but the length of groups is a result, consider moving down | 177-178 | 10 | This section has been reworked to strengthen the whole section on research design, sampling, sample size determination as highlighted by Reviewer # 3. The length of the groups has been adjusted |
| Results | 168 | If men owned 65% of the livestock, does this mean women owned 35% of all recorded livestock? Adding numerators and denominators can help with this interpretation, but it would nonetheless be nice to clarify. If men owned 40% of cows, did women own 60% of the cows, or did 40% of the men in the study own cows? | 206-211 | 12 | Numerators and denominators have been provided to help with this interpretation and presentation |
|  | 203 | Believe we are missing the word net here…looks like men purchase mosquitos | 229 | 10 | The word net has been introduced |
|  | 229 | Change men to male | 266 | 14 | Men have been changed to male |
|  | 288 | Comma after RVF | 325 | 17 | A comma has been introduced after RVF |
| Reviewer #2 |  |  |  |  |  |
|  |  | All the points have been addressed and I think it can be published after doing some language checking by an expert. | All manuscript sections |  | A comprehensive language review was conducted throughout the manuscript to reduce the use of colloquial language and enhance the clarity and formality of the text. |
| Reviewer # 3 |  |  |  |  |  |
|  |  | No correction at all, not addressed anything at all, simply ignored. They need to be make it clear with major revision. |  |  | All the comments have been considered and addressed point by point as indicated in the table and in the manuscript |
|  | **Original manuscript line number:** |  |  |  |  |
|  | 125 & 132 | Focus on study design again, and sample size determination. (Where is the sample size?) | Study design: 116-121 | 6  7 | The whole section on “study design and data collection has been reworked for clarity and to strengthen the section |
|  |  |  | Sample size determination: 123-135 |  |  |
|  | 116 | Need to mention qualitative study | 108 | 5 | The concept of qualitative study was introduced in the study design section |
|  | 255 | Analysis transparency need to be more clear | 178-191 | 9-10 | The whole section on “data management and analysis” has been revised to capture the details of the data analysis process |
|  | 437-635 | The discussion part can be short based on key findings | 350-467 | 22 | The discussion has revised and summarized to capture key findings |
|  | 637 | A conclusion should be result-oriented with a focus one future implications. (Must be focus on key result findings & then recommendation in one or two separate sentence) | 469-485 | 22-23 | The conclusion has been revised to reflect the study results and implications, and further research |
|  | 734-744, 755,773-774,787-791,824-826,831-834 | Need to generate the latest data | 4632, 588-592, 551-552, 556-568 | 26-27,30, | References 18, 19, 20, 21, 25, 32, 34,37, 48 and 50 have been removed and more recent literature has been introduced. The current manuscript obtains from references (2014 to 2024) except 33 and 34. The reason why these two have been maintained, we could not find a suitable reference to replace them. |
