# Supplementary material for: “Without a man’s decision, nothing works”: Building resilience to Rift Valley fever in pastoralist communities in Isiolo Kenya
Source: PLoS One. 2025 Jan 28;20(1):e0316015. doi: 10.1371/journal.pone.0316015 (PMC11774392; doi:10.1371/journal.pone.0316015)
Supplement: Attachment — Submitted filename: Response to Reviewers--.docx [file pone.0316015.s005.docx]

**Rebuttal Letter**

Dear Editor,

Thank you for the opportunity to revise and resubmit our manuscript, "Without a Man’s Decision, Nothing Works”: Building Resilience to Rift Valley fever in Pastoralist Communities in Isiolo Kenya" [PONE-D-24-03443]. We appreciate the time and valuable feedback provided by the reviewers and editorial team. We have carefully addressed each concern, and we believe these revisions have significantly strengthened the manuscript.

Enclosed please find the following:

- **Response to Reviewers:** A point-by-point response to each comment, outlining the specific changes made in the manuscript.
- We have reviewed the reference list for completeness and accuracy. We can confirm the references are complete and correct, and no retracted papers have been included.
- This revision is based on the clean manuscript submitted in **Round Two** and as a result, the line numbers may differ. However, to ensure consistency, we have added “new comment” in the manuscript with track changes for easy identification of the reviewer’s comments

We are confident that our revised manuscript now meets the high standards of PLOS One. We are grateful for your consideration and look forward to your decision.

Sincerely,

Nabwire Irene Mutambo

Response to the reviewers’ comments

| **Section** | **Comment line for the manuscript round two** | **Comment** | **Response line** | **Page number** | **Response to the comments** |
| --- | --- | --- | --- | --- | --- |
| Reviewer #1 |  |  |  |  |  |
| Method section | Not provided | Spell out numbers less than ten. 4=four | 135,148 | 8 | All the number less than ten have been spelt out |
|  | Line 138 | Typo involving | 132 | 8 | The typo has been resolved |
|  | Line 176 | Typo between | 171 | 10 | The typo has been resolved |
| Results | Line 290 | These statements still do not make sense. I believe you mean, 95% of men owned cows, 70% of men owned goats, ect? Why not write it simply like this? If the proportion is of men (not cows) then the % associated belongs next to men, not cows. | 209-212 | 12 | The statement has been revised to improve result clarity |
| Discussion | Line 468 | This sentence fades away with no end? | 372-374 | 19 | The sentence has been revised to be complete |
|  | Line 480 | A citation cannot replace words. You can say, “A study in x reported___” but not just putting the citation. | 385-386, 389 | 19 | The sentence has been revised. The in-text citation has been removed and placed at the end of the sentence. |
|  | Line 552 | Resonates how? | 417-419 | 21 | How the results resonate with our finding has been provided |
|  | Line 590 | A switch to third person point of view “The researcher” Needs to be consistent throughout 1st person “We” | 448 | 22 | A switch to third persons’ view have remove and replaced with “we” as suggested by the reviewer. |
|  | Line 639 | Another example of these in-text citations that do not make sense. | 479-481 | 24 | The sentence has been revised. The in-text citation has been removed and placed at the end of the sentence. |
|  | Line 644 | Another example of in-text citation | 485-489 | 24 | The sentence has been revised. The in-text citation has been removed and placed at the end of the sentence. |
|  |  | Line numbers for the formatted references | 544, 563-566, 569, 571,573, 576, 579, 583, 587, 590, 596, 608, 609, 611-613, 614, 625-627, 629, 635, 645, 648, 654-656, 658, 661, 662-663, 665, 666-669, 673 | 26-32 | All the reference list has been reworked for completeness and accuracy |
